# The trends and associated adverse maternal and perinatal outcomes of labour neuraxial analgesia among vaginal deliveries in China between 2012 and 2019: a real-world observational evidence

**DOI:** 10.1186/s12916-021-01941-6

**Published:** 2021-03-19

**Authors:** Yi Mu, Xiaodong Wang, Yanping Wang, Zheng Liu, Mingrong Li, Xiaohong Li, Qi Li, Jun Zhu, Juan Liang, Haidong Wang

**Affiliations:** 1 National Office for Maternal and Child Health Surveillance of China, West China Second University Hospital, Sichuan University, Ren Min South Road Section 3 No.17, Chengdu, Sichuan China; 2 Department of Obstetrics, West China Second University Hospital, Sichuan University, Ren Min South Road Section 3 No.17, Chengdu, Sichuan China; 3 Key Laboratory of Birth Defects and Related Diseases of Women and Children (Sichuan University), Ministry of Education, Ren Min South Road Section 3 No.17, Chengdu, Sichuan China; 4 Institute for Health Metrics and Evaluation, University of Washington, Seattle, WA USA

**Keywords:** Labour neuraxial analgesia, Intervention, China, Bayesian multilevel model, Interrupted time-series analysis, Propensity score, Maternal near miss

## Abstract

**Background:**

There is a lack of national report of the labour neuraxial analgesia (NA) rates in China in recent years, especially after the national promotion policy. The adverse maternal and perinatal outcomes associated with NA in China are also unknown. The aim of this study is to estimate the trends of NA rates from 2012 to 2019, to evaluate the effect of national policy on promoting NA and to identify the association between NA and adverse outcomes in China.

**Methods:**

We used the individual data from China’s National Maternal Near Miss Surveillance System (NMNMSS) between 2012 and 2019, covering 438 hospitals from 326 urban districts or rural counties in 30 provinces across China. The analysis was restricted to singleton pregnant women who underwent vaginal delivery at or after 28 completed weeks of gestation. We estimate the trends of NA rates between 2012 and 2019, both at the national and provincial levels using Bayesian multilevel model. We also estimated the effect of the national pilot policy launched in 2018 using interrupted time-series analysis and identified the association between NA and adverse outcomes using modified Poisson regression combined with propensity score analysis.

**Results:**

Over the study period, 620,851 of 6,023,046 women underwent vaginal delivery with NA. The estimated national NA rates increased from 8.4% in 2012 to 16.7% in 2019. Most provinces experienced the same rapid rise during this period. The national pilot policy accelerated the rise of the rates. No differences were observed between women with NA and without any analgesia in the incidence of uterine atony, placental retention, intrapartum stillbirths and 1- and 5-min Apgar scores lower than 7. However, women with NA had higher incidences of genital tract trauma (adjusted relative risk (aRR) 1.53, 95% confidence interval (CI) 1.04–2.26) and maternal near miss (aRR 1.35, 95% CI 1.08–1.69), only in hospitals which were not covered by the national pilot policy and usually lack of sufficient equipment and personnel.

**Conclusions:**

The national policy can effectively increase the NA rate. However, as genital tract trauma and maternal near miss may increase in low-resource hospitals, but not in high-resource hospitals, further study is required to identify the reasons.

**Supplementary Information:**

The online version contains supplementary material available at 10.1186/s12916-021-01941-6.

## Background

Labour pain may be the most severe pain that many women experience in their lifetime, and every woman has the right to choose a form of pain management during labour [1]. With the development of anaesthesiology and the demand for pain relief in labour, labour neuraxial analgesia, e.g., epidural analgesia (EA) and combined spinal-epidural analgesia (CSEA), has become widely used in developed countries [2]. A national survey among anaesthesiologists between 2003 and 2004 in China estimated that the national labour neuraxial analgesia rate was less than 1% [3], much lower than the rates in developed countries. Actions by Ling-Qun Hu and his colleagues have played a positive role in popularizing labour analgesia in China [4]. The National Health Commission issued two policies in August and November 2018 to promote labour neuraxial analgesia in China, which have been officially implemented in pilot hospitals since January 2019 [5, 6]. The national policy mainly includes five main elements: improving the skills of labour analgesia, improving the scientific choice of delivery mode, enhancing the awareness of hospitals and relevant departments of labour analgesia, strengthening health education for pregnant women and their families, making full use of the demonstration and driving role of pilot hospitals. However, national reports on the trends of the labour neuraxial analgesia rate in China and the effect of the national policy are currently lacking.

The risk of adverse maternal and perinatal outcomes has played an important role in the promotion of labour neuraxial analgesia. A Cochrane systematic review reported that compared with non-epidural or no pain relief during labour, EA (including CSEA) had an increased risk of assisted vaginal birth, maternal hypotension, maternal fever and some other complications [7]. Most studies focus on anaesthesia-related complications. However, there were no trials regarding rare but potentially severe adverse maternal (such as maternal near miss) or long-term neonatal outcomes [7]. An observational study with large samples may help to explore the association. Because randomized controlled trials (RCTs) are often limited in terms of generalizability [8], the evidence provided by real-world observational studies can complement the findings from RCTs [9]. Previous studies on the association between labour neuraxial analgesia and adverse maternal and perinatal outcomes in China are mostly limited in single hospital with small sample sizes [10, 11].

In our study, data from China’s National Maternal Near Miss Surveillance System (NMNMSS) were used to estimate the trends of labour neuraxial analgesia rates between 2012 and 2019, both at the national and provincial levels. We also estimated the effect of the national pilot policy launched in 2018 and identified the association between labour neuraxial analgesia and adverse maternal and perinatal outcomes in China. The primary outcomes were maternal near miss (MNM) and intrapartum stillbirths. The secondary outcomes included three main causes of postpartum haemorrhage (genital tract trauma, uterine atony, placental retention) and 1- and 5-min Apgar scores lower than 7. We used up to six types of propensity score analysis, as well as *E-V*alue, to enhance the reliability of the results.

## Methods

### Data sources

Individual-level data covering births in hospitals from 1 January 2012 to 31 December 2019 and institutional data from each hospital in 2015 were collected through the NMNMSS, a national registry system. The NMNMSS was established by the National Health Commission at the end of 2010, using the same approach suggested in the World Health Organization’s (WHO) global survey on maternal and perinatal health [12]. The number of births per year in NMNMSS was over 1.2 million, accounting for about 8% of the total number of births in China. The sampling urban districts and rural counties of the NMNMSS, which the hospitals located in, were based on China’s National Maternal and Child Health Surveillance System and Provincial-level Maternal and Child Health Surveillance Systems. Once the urban districts or rural counties were selected, two public hospitals located in each area with more than 1000 deliveries per year and regional representation were randomly selected (or one hospital if only one was available) into the NMNMSS. For each pregnant or postpartum woman admitted to the obstetrics department, maternal sociodemographic and obstetric characteristics, medical interventions during hospitalization, and maternal and perinatal outcomes before discharge were collected by the responsible doctor through medical records. The unified survey form and definition of MNM were used in all the hospitals. There were multiple levels of quality control in the NMNMSS, including hospital-level, county-level, municipal, provincial and national quality control. The sampling strategy, data collection and reporting processes and quality control method of the NMNMSS have been described in detail elsewhere [13, 14]. The data provided to us was de-identified.

Data on the number of live births per year in each of the 30 provinces of China (except Tibet) for 7 age groups of women (< 20 years, 20–24 years old, 25–29 years old, 30–34 years old, 35–39 years old, 40–44 years old and ≥ 45 years old) from 2012 to 2019 estimated from the Global Burden of Disease (GBD) study were also used in the study [15].

### Definitions

We restricted our analysis to singleton pregnant women who underwent vaginal delivery at or after 28 completed weeks of gestation in all the hospitals in the NMNMSS between 2012 and 2019. Women who received analgesia after delivery were excluded. As we did not collect the information on labour, women who had a vaginal trial but switched to an emergency caesarean section (CS) were also excluded.

EA is provided by placing a catheter into the lumbar epidural space, through which a local anaesthetic, opioid, or both are infused [16]. CSEA involves a single injection of local anaesthetic, opioid, or both into the cerebral spinal fluid as well as the insertion of an epidural catheter [16]. Labour neuraxial analgesia is usually initiated by one of these two methods [17]. The hospital level (from level 1 to level 3) was certified by the administrative department of health. It was classified according to the number of beds, categories of clinical departments, numbers of medical personnel, type and quantity of equipment and hospital funding, where level 3 hospitals had more advanced care.

The criteria for MNM identification followed the WHO definition [18, 19], including three categories of life-threatening indicators: clinical crieria, laboratory-based criteria and management-based criteria. Since a variety of pregnancy complications were recorded in the NMNMSS, we classified all pregnancy complications into three categories: antepartum complications, postpartum complications and medical diseases. Antepartum complications included: antepartum haemorrhage (ruptured uterus, placenta previa, placental abruption and unspecified antepartum haemorrhage), hypertensive disorders in pregnancy (chronic hypertension, gestational hypertension, preeclampsia, eclampsia and HELLP syndrome), any foetal malpresentation (breech, shoulder, or other), premature rupture of membranes and polyhydramnios or oligohydramnios. Postpartum complications included: postpartum haemorrhage (genital tract trauma (only II-degree and above were reported), uterine atony, placental retention, placenta accrete, unspecified postpartum haemorrhage) and puerperal infection. Medical diseases were heart disease, embolism or thrombophlebitis, hepatic disease, severe anaemia (haemoglobin concentration of < 70 g/L), renal disease (including urinary tract infection), lung disease (including upper respiratory tract infection), HIV/AIDS, connective tissue disorders, gestational diabetes mellitus, intrahepatic cholestasis of pregnancy, hypothyroidism and cancer.

January 1, 2019, was set as the time point to distinguish between before and after the implementation of the national policy [6]. The Hospital Administration Bureau of the National Health Commission established the basic standards for the identification of pilot hospitals (for example, hospitals should have professional and technical personnel to provide labour analgesia, the previous labour analgesia rate in the hospital was no less than 10%, etc.) and assessment requirements. The provincial health administrative departments were responsible for organizing and implementing the local pilot work, including pilot hospital selection, identification (based on the standards), training, guidance and assessment. Neither the pilot hospitals nor the non-pilot hospitals had specific financial support. Whether the hospital in the NMNMSS was a pilot hospital in the national project was confirmed through the list of 913 hospitals published on the website of the National Health Commission [20]. Only a part of hospitals in the national project was also the hospitals in the NMNMSS.

### Statistical analysis

First, we summarized the individual data from the NMNMSS to obtain the observed province-year EA rates. To better represent the provincial level, we obtained the provincial age-specific weights by comparing the number of provincial age-specific live births from the GBD study (assuming that it represents the actual distribution of the population) and the NMNMSS, and then adjusted the EA rates with these weights. We modelled logit transform of the weighted EA rates using a Bayesian multilevel linear mixed regression model, with correlated random province-specific intercept and time slope. Uncertainty intervals (UIs) were constructed from the 2.5th and 97.5th percentiles of the posterior samples. We performed the same procedure to estimate the CSEA rates. The labour neuraxial analgesia rates were generated by adding the EA and CSEA rates of each province and year. National estimates for each year were generated by weighting provincial estimates according to the number of live births in each province from the GBD study. The estimating of EA and CSEA rates was further detailed in Additional file 1: Method S1.

Second, we used interrupted time-series analysis (ITSA) [21] with ordinary least-squares (OLS) regression models adjusted for autocorrelation [22] to quantify changes in the national labour neuraxial analgesia rates after the national pilot policy. Two types of monthly data aggregated from the individual data from the NMNMSS were used in the analysis: one is that all hospitals are aggregated into one data set, and the other is that pilot and non-pilot hospitals are aggregated separately and recombined into one data set. The ITSA is further detailed in Additional file 1: Method S2.

Finally, we examined strength of the association between labour neuraxial analgesia and the adverse maternal and perinatal outcome. To ensure the reliability of the assessment for the association, we restricted the analysis to women without any antepartum complications or medical diseases. We restricted the sample to births without antepartum stillbirths for the analysis of intrapartum stillbirths and to live births for the analysis of 1- or 5-min Apgar scores lower than 7. We reported crude relative risks (cRRs) and adjusted relative risks (aRRs) with 95% confidence intervals (CIs) of labour neuraxial analgesia for maternal outcomes and perinatal outcomes from eight models, using modified Poisson regression with a robust variance estimator [23, 24] and clustering of births within the same hospitals. The adjusted confounding factors included year, region, hospital level, the number of anaesthesiologists per 1000 births, the number of antenatal care visits, maternal education, marital status, maternal age, gestational age and parity. For MNM, we further adjusted postpartum complications. We used six types of propensity score (PS) analysis to reduce the observed selection bias in estimating labour neuraxial analgesia risk and to reduce the likelihood of confounding when analysing observational data [25–27]. All the models were further detailed in Additional file 1: Method S3. We repeated the above analysis in all pregnant women (including those with antepartum complications and medical diseases) for sensitivity analysis. Since PS analysis cannot balance the unobserved confounding factors in observational studies [26], we estimated the E-Value for all the statistically significant results, to assess the potential effect of unobserved confounding [28, 29]. We also carried out marginal effect analysis [30] to observe the time trends of rate differences (RDs) of adverse maternal and perinatal outcomes with statistically significant results between women with labour neuraxial analgesia or women without any analgesia in pilot and non-pilot hospitals, respectively.

If outcome variables or covariates had missing data, the cases were excluded from the model. All statistical analyses were conducted using Stata version 16.1 (StataCorp) with 2-tailed tests and a significance level of *P* < 0.05.

### Ethics approval

This study was approved by the ethics committee of the West China Second University Hospital (protocol ID, 2012008).

## Results

### Trends over time in labour neuraxial analgesia rates

**Fig. 1:**
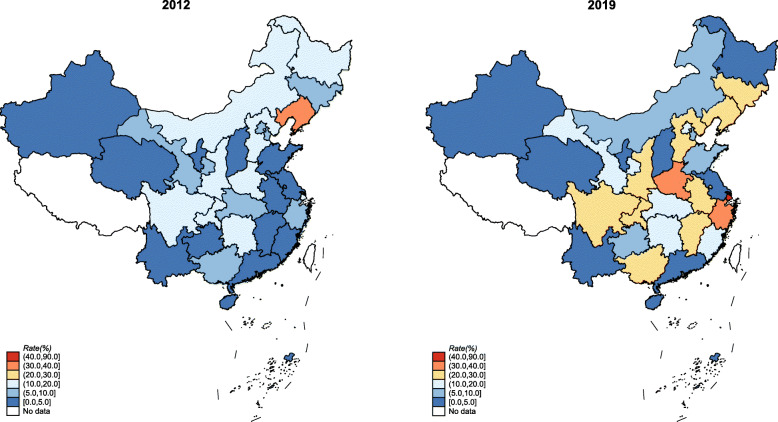
The change of Bayesian estimated provincial rates of labour neuraxial analgesia among vaginal deliveries in China from 2012 to 2019

Between 2012 and 2019, the individual information of 10,835,501 singleton women whose delivery at or after 28 completed weeks of gestation were collected in the NMNMSS. 4,812,455 cases were excluded as they underwent caesarean section or vaginal delivery but with unknown analgesia method. Finally, 6,023,046 singleton pregnant women who underwent vaginal delivery at or after 28 completed weeks of gestation were included in the study (Additional file 1: Fig. S1). A total of 620,851 women delivered with labour neuraxial analgesia. Diagnostic plots showed that the multilevel Bayesian models performed well to predict the rates (Additional file 1: Fig. S2-S3). The national labour neuraxial analgesia rates increased from 8.4% (95% UI 6.6–10.5) in 2012 to 16.7% (95% UI 13.5–20.6) in 2019 (Table 1). Among 14 of the 30 provinces, the labour neuraxial analgesia rates in 2019 are more than twice those of 2012 (Fig. 1). The trends of EA and CSEA were similar to labour neuraxial analgesia (Additional file 1: Table S1-S3 and Fig. S4).

**Table 1:** The national rates and 95% uncertainty intervals (UIs) of labour neuraxial analgesia among vaginal deliveries in China (%)

Year	Labour neuraxial analgesia		Epidural analgesia		Combined spinal-epidural analgesia	
	Observed	Bayesian estimate (95% UI)	Observed	Bayesian estimate (95% UI)	Observed	Bayesian estimate (95% UI)
2012	7.7	8.4 (6.6–10.5)	4.2	4.7 (3.6–6.0)	3.5	3.6 (2.8–4.6)
2013	8.7	8.7 (6.9–10.9)	4.7	4.7 (3.6–6.1)	4.0	4.0 (3.1–5.1)
2014	8.9	8.4 (6.6–10.5)	4.6	4.3 (3.3–5.6)	4.3	4.1 (3.2–5.2)
2015	9.4	9.1 (7.2–11.4)	4.6	4.5 (3.5–5.8)	4.8	4.6 (3.5–5.9)
2016	9.7	9.4 (7.4–11.8)	4.6	4.6 (3.5–5.8)	5.0	4.9 (3.8–6.2)
2017	8.8	7.0 (5.5–8.9)	4.6	3.8 (2.9–4.9)	4.1	3.2 (2.5–4.2)
2018	12.0	10.9 (8.6–13.6)	6.6	5.9 (4.6–7.5)	5.4	5.0 (3.8–6.4)
2019	16.8	16.7 (13.5–20.6)	9.6	9.7 (7.7–12.0)	7.1	7.0 (5.5–9.0)

### The effect of the national pilot policy on promoting labour neuraxial analgesia

**Fig. 2:**
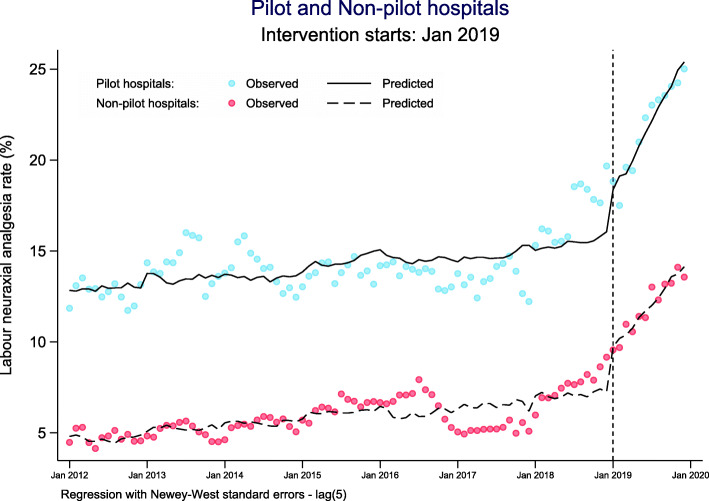
Multiple-group ITSA for comparisons on national monthly changes of labour neuraxial analgesia rates after the national policy. Notes: *ITSA* interrupted time-series analysis; adjustment for time-varying covariates: proportion of maternal age ≥ 35 years old, women with college education or above, number of antenatal visits ≥ 7 and women with antepartum complications or medical diseases

A total of 125 hospitals from the NMNMSS have been selected as pilot hospitals, accounting for 13.7% (125/913) of all pilot hospitals in the national policy and 28.5% (125/438) of all hospitals in the NMNMSS. The pilot hospitals in the NMNMSS are mainly Level 3 hospitals, while non-pilot hospitals are mainly Level 2 hospitals (Additional file 1: Table S4). The autoregressive test results are detailed in Additional file 1: Table S5. Both single- and multiple-group ITSA results show that the national pilot policy has significantly increased the labour neuraxial analgesia rate (Table 2, Fig. 2 and Additional file 1: Fig. S5). The results of multiple-group ITSA shows that, in the baseline period (from Jan 2012 to Dec 2018), although the average labour neuraxial analgesia rate of pilot hospitals was higher than that of non-pilot hospitals, there was no significant difference in the trend between the two groups (*p* = 0.578). After the implementation of the national policy, there was no difference in the level of increase in labour neuraxial analgesia rates immediately following the policy (the first month after the implementation of the policy, that is, January 2019) between the pilot and non-pilot hospitals (*p* = 0.808). However, the long-term trend (all months after the implementation of the policy, that is, from January to December 2019) was faster in pilot hospitals than in non-pilot hospitals (*p* = 0.001). Sensitivity analysis shows that the long-term trend of pilot hospitals was faster than that of non-pilot hospitals since September 2018 (Additional file 1: Table S6). Before that, the trend difference between the two groups was not statistically significant.

**Table 2:** The estimate changes of national labour neuraxial analgesia rates (%) after the national policy

Variables	Crude model ^a^				Adjusted model ^a, b^			
	*β* (coefficient)	*P* value	95% CI		*β* (coefficient)	*P* value	95% CI	
			Lower	Upper			Lower	Upper
**Single-group (all hospitals)**	Maximum lag: 4				Maximum lag: 4			
Baseline intercept (*β*_0_)	7.61	< 0.001	7.02	8.19	23.85	0.001	10.02	37.68
Baseline slope (*β*_1_)	0.04	< 0.001	0.02	0.06	0.15	0.010	0.03	0.26
Intercept change (post-intervention) (*β*_2_)	2.77	< 0.001	1.39	4.15	1.35	0.101	− 0.27	2.96
Slope change (post-intervention) (*β*_3_)	0.48	< 0.001	0.41	0.56	0.46	< 0.001	0.33	0.59
**Single-group (pilot hospitals)**	Maximum lag: 5				Maximum lag: 4			
Baseline intercept (*β*_0_)	12.82	< 0.001	11.85	13.80	24.73	0.011	5.72	43.75
Baseline slope (*β*_1_)	0.03	0.029	0.00	0.06	0.14	0.055	0.00	0.27
Intercept change (post-intervention) (*β*_2_)	2.65	0.005	0.83	4.46	1.54	0.129	− 0.46	3.54
Slope change (post-intervention) (*β*_3_)	0.63	< 0.001	0.53	0.72	0.67	< 0.001	0.50	0.84
**Multiple-group (Pilot and non-pilot hospitals)**	Maximum lag: 6				Maximum lag: 5			
Baseline intercept of non-pilot hospitals (*β*_0_)	4.64	< 0.001	4.24	5.05	9.36	0.039	0.48	18.23
Baseline slope of non-pilot hospitals (*β*_1_)	0.03	< 0.001	0.01	0.05	0.10	0.056	0.00	0.21
Intercept change of non-pilot hospitals (post-intervention) (*β*_2_)	2.47	< 0.001	1.36	3.58	2.16	< 0.001	1.02	3.30
Slope change of non-pilot hospitals (post-intervention) (*β*_3_)	0.38	< 0.001	0.34	0.42	0.40	< 0.001	0.30	0.49
Difference of the intercept between pilot and non-pilot hospitals (prior-intervention) (*β*_4_)	8.18	< 0.001	7.1	9.26	13.01	< 0.001	6.58	19.44
Difference of the slope between pilot and non-pilot hospitals (prior-intervention) (*β*_5_)	0.00	0.899	− 0.03	0.03	−0.01	0.578	− 0.04	0.02
Difference of the change of intercept immediately following intervention between pilot and non-pilot hospitals (*β*_6_)	0.18	0.874	− 2.02	2.38	−0.28	0.808	− 2.53	1.98
Difference of the slope between pilot and non-pilot hospitals (post-intervention versus prior-intervention) (*β*_7_)	0.25	< 0.001	0.15	0.35	0.23	0.001	0.09	0.37

*CI* confidence interval

^a^The model is estimated using Newey-West standard errors to handle autocorrelation

^b^The model is adjusted for the proportion of maternal age ≥ 35 years old, women with college education or above, number of antenatal visits ≥ 7 and women with antepartum complications or medical diseases

### The association between labour neuraxial analgesia and adverse maternal and perinatal outcomes in China

**Fig. 3:**
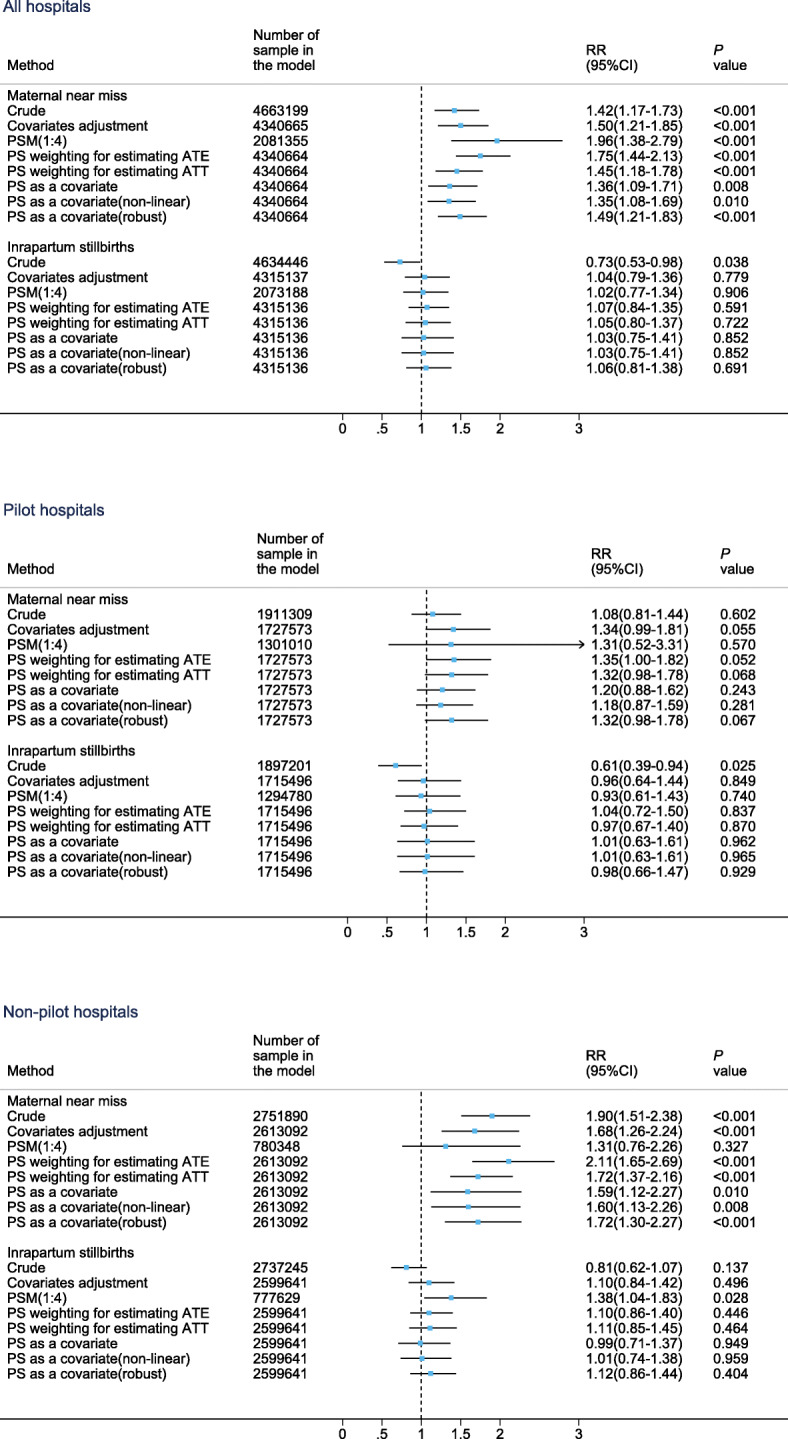
Comparison of primary maternal and perinatal outcomes between women with labour neuraxial analgesia and without any analgesia. Notes: *PSM* propensity score matching, *PS* propensity score, *ATE* average treatment effect where weight is 1/*PS* for a treated case and 1/(1-*PS*) for a comparison case, *ATT* average treatment effect for the treated cases where weight is 1 for a treated case and *PS*/(1-*PS*) for a comparison case. The left side of the reference line (short dash line in the figure) means lower risk, and the right side means higher risk

The distribution of potential confounding factors between women with labour neuraxial analgesia and without any analgesia is shown in Additional file 1: Table S7. When assessing the risk of adverse maternal and perinatal outcomes for labour neuraxial analgesia, 1,359,847 pregnant women with antepartum complications or medical diseases were excluded. In both the traditional covariates adjustment model and the propensity score analysis, we observed no differences in the incidence of uterine atony, placental retention, intrapartum stillbirths and 1- and 5-min Apgar scores lower than 7 (Fig. 3 and Additional file 1: Fig. S6-S7). However, we found that women with labour neuraxial analgesia were associated with increased risk for genital tract trauma and MNM. The sensitivity analysis in all pregnant women also obtained similar results (Additional file 1: Fig. S8). The results of the traditional covariates adjustment model were very close to those of PS analysis, except for the propensity score matching (PSM) approach in some outcomes. Although PSM reduced the bias of covariates between women with labour neuraxial analgesia and without any analgesia (Additional file 1: Fig. S9), it dropped many observations, and the extrapolation of the results was limited; therefore, we mainly focused on the results of other PS analysis approaches. Only a very small number of observations in the treatment group were excluded in the PS analysis, as they were not in the common support region (Additional file 1: Fig. S10). For genital tract trauma, the PS weighting for estimating treatment effect for the treated cases had the smallest RR with statistical significance (aRR 1.53, 95% CI 1.04–2.26). For MNM, the model using PS as the only one covariate (non-linear relationship between PS and MNM) had the smallest RR with statistical significance (aRR 1.35, 95% CI 1.08–1.69). Further subgroup analysis found that labour neuraxial analgesia was associated with genital tract trauma and MNM only in non-pilot hospitals. The E-values (relative risks) for genital tract trauma and MNM in all hospitals were 2.43 and 2.04 (Additional file 1: Fig. S11). Marginal effect analysis showed that there were no significant RDs of genital tract trauma and MNM between women with labour neuraxial analgesia and women without any analgesia in pilot hospitals, but there were significant RDs in non-pilot hospitals, with an upward trend from 2012 to 2019 (Additional file 1: Fig. S12). The top three indicators of MNM cases with labour neuraxial analgesia are massive blood transfusion (56.1%), shock (32.4%) and platelet count less than 50,000/ul (17.8%).

## Discussion

Our study, for the first time, showed that the national labour neuraxial analgesia rates among women with vaginal delivery increased rapidly from 2012 to 2019 in China. Most provinces experienced the same rapid rise during this period. The national pilot policy played an important role in the promotion of the labour neuraxial analgesia in China. Compared to women without any analgesia, women with labour neuraxial analgesia were associated with more genital tract trauma and MNM in non-pilot hospitals, but not pilot hospitals.

Labour neuraxial analgesia has been practised and popularized worldwide since the twentieth century. However, wide variations in the use of EA have been reported, even in developed countries [2]. The standardized promotion of labour neuraxial analgesia among a large number of women in a single hospital had only been available in China since 2001 [31]. To slow the increase in CS rates and improve women’s health, Chinese obstetric experts have proposed popularizing labour neuraxial analgesia among vaginal deliveries in China [32]. Several relevant actions have been taken [4, 33]. Demand from society and actions from academia eventually led the government to launch a national policy to promote labour neuraxial analgesia in 913 pilot hospitals at the end of 2018 [6]. Although the rate is obviously higher than in the past, there is still a large gap in labour neuraxial analgesia rates between China and developed countries.

The factors that influence the popularization of labour neuraxial analgesia are complex, such as education and parity [32, 34–37]. Reimbursement for neuraxial analgesia may increase hospital income and thus serve as an incentive, but could lead to the overuse of labour analgesia in some hospitals [2]. Nevertheless, there are some more important contributing factors to the low labour neuraxial analgesia rate in China: preference to have a CS during the “One-Child” period, unreasonable charging standards for labour analgesia among vaginal deliveries [3], and shortage of anaesthesiologists (0.5, 2.5 and 2.8 anaesthesiologists per 10,000 population in China, the USA and the UK, respectively) [33]. Although the number of anaesthesiologists at level 3 hospitals (especially general hospitals) is relatively large, the anaesthesiologists are more willing to participate in surgery than labour neuraxial analgesia [3, 33].

However, the demand from society for vaginal delivery has changed since the implementation of the “Universal Two-Child” policy [38]. The proportion of women who choose vaginal delivery during their first birth may increase to ensure that it is safe to give birth to their second child. Labour analgesia can relieve labour pain and further promote women’s willingness to choose vaginal delivery. After the national pilot policy, the labour neuraxial analgesia rate has been increasing more rapidly. Our research also shows that the effect of this policy began to appear even as early as September 2018. This time node is consistent with the government’s notice of strengthening and improving anaesthesia medical service, including popularizing labour analgesia [5]. In addition, the national policy has clear spill over effects, as the labour neuraxial analgesia rate after the policy is also significantly faster than that before the policy in non-pilot hospitals. The National Health Commission’s purpose of setting up the pilot hospitals in the policy is to popularize the technology of labour analgesia on the basis of ensuring the safety of mothers and infants. These pilot hospitals are often those with sufficient anaesthesiologists, higher capability of obstetrical services and support from experienced multidisciplinary rescue teams [6]. In the NMNMSS, there are great differences in hospital-level distribution between non-pilot and pilot hospitals. The proportion of level 3 hospitals in pilot hospitals is much higher than that in non-pilot hospitals. The labour neuraxial analgesia rate in non-pilot hospitals has also risen rapidly, which may be due to the demand of economic interests and blindly following the national policy. It may bring challenges to the safety of mothers and infants.

Systematic reviews and clinical guidelines for labour neuraxial analgesia have identified some advantages and disadvantages [7, 34, 39]. However, some areas, such as the risk of postpartum obstetric complications, MNM, and even maternal death, remain uncertain [7, 39]. Our study suggests that labour neuraxial analgesia may be associated with increased risk for genital tract trauma and MNM. The consistency of the results from traditional covariates models and several approaches of PS analysis prove the robustness and reliability of the findings. In China, pregnant women usually talk to their anaesthesiologist and obstetrician when they reach 36–38 weeks gestation to see whether vaginal trial of labour and labour analgesia is needed. Very few women decide on the need for labour analgesia until close to the onset of labour. Women who receive labour neuraxial analgesia are subject to strict screening for indications and contraindications (such as abnormal coagulation) [40]. Our analysis on the association between labour neuraxial analgesia and adverse maternal and perinatal outcomes has excluded women with any antepartum complications or medical diseases to eliminate the interference of diseases that existed before labour neuraxial analgesia. In addition, analysis of E-Value enhances the robustness of these findings, as it is almost impossible to have such a strong potential unobserved confounding factor in addition to the confounding factors that have been adjusted by the model. Therefore, our findings are highly meaningful for clinical practice.

Previous studies reported that no significant relationship between epidural usage and genital tract trauma [41–43]. Timothy et al. even reported that epidural was negatively associated with laceration [44]. Compared with our study (the sample size is more than 4 million), the sample size of these previous studies was very small, the lowest is only about 200 [43] and the highest is less than 6000 [42]. In addition, Timothy et al.’s study included operative vaginal delivery as a mediator in the regression model [44], possibly resulting in over-adjustment [45], and failing to correctly estimate the total effect of epidural analgesia. Changes in obstetric management could mediate the associations found in the current study.

A secondary analysis of the WHO Multicountry Survey, from another perspective, reported that women with severe maternal morbidity (SMM) were associated with a higher use of labour analgesia than those who did not experience SMM [46]. We also confirm our previous concerns about adverse effects caused by policy spill over: labour neuraxial analgesia increased the risk of genital tract trauma and MNM only in non-pilot hospitals. All these findings suggest that promoting labour neuraxial analgesia requires care and caution.

To ensure the safety of the mothers who will choose labour neuraxial analgesia in the future, we have the following suggestions. First, labour neuraxial analgesia should be used upon request rather than routinely [47–49]. Labour neuraxial analgesia may increase some risks, after all, it is a kind of human medical intervention. Reducing over-intervention in childbirth and providing maternity-centred services to ensure maternal safety during childbirth is the consensus of experts [2]. Second, hospitals without enough anaesthesiologists or comprehensive rescue capacity should not be allowed to provide labour neuraxial analgesia services [31, 50]. We do not warn against the use of labour neuraxial analgesia during vaginal delivery. We believe the health risk from labour neuraxial analgesia is controllable. However, it is difficult for level 1 hospitals and even some level 2 hospitals to provide adequate medical resources (anaesthesiologists, midwives and blood sources) to ensure safety. There are hidden risks in technique details of labour neuraxial analgesia. That is why we do not recommend blindly following the promotion of labour neuraxial analgesia in hospitals without adequate safety guarantees. Thus, most level 1 and level 2 hospitals that lack resources should focus more on non-pharmacological interventions (such as doula care or immersion in water) in the future. Third, there is an urgent need for formal clinical guidelines on labour analgesia. The Chinese Anaesthesiology Association published the “Expert Consensus on Labour Analgesia” in 2016 to guide clinical practice but did not provide details on the pros and cons of labour analgesia [40]. Referring to guidelines from other countries, such as American College of Obstetricians and Gynecologists [34], the formal guidelines in China should provide detailed specifications on how anaesthesiologists monitor maternal vital signs for labour neuraxial analgesia, including how to collaborate with other health care team members. Considering that severe postpartum haemorrhage is the main manifestation of MNM with labour neuraxial analgesia, the interventions for postpartum haemorrhage need to be included in the guidelines. Trainings at all levels for labour analgesia, especially for emergency handling of sudden problems, are also very important.

There are also several limitations in this study. First, due to the lack of labour information, women who laboured, intending vaginal delivery, but ultimately required delivery by emergency CS were excluded. Therefore, the findings of this study are only applicable to women whose final delivery method is vaginal delivery. Second, since the women who had intrapartum caesarean section were excluded, this study cannot assess the benefit of labour neuraxial analgesia from avoiding general anaesthesia when rapid conversion from epidural analgesia to anaesthesia is needed in an emergency. Third, as this study was an observational study, it can only suggested the association between labour neuraxial analgesia and adverse outcomes. In addition, due to the limited information that can be collected in the NMNMSS, our study is unable to further explore the biologic plausibility of labour neuraxial analgesia being directly related to postpartum haemorrhage. However, the findings of this study will provide direction for the next step in the design of more powerful causal arguments research.

## Conclusions

Our study shows that national policy can effectively increase the labour neuraxial analgesia rate. However, as genital tract trauma and MNM may increase in low-resource hospitals (such as most of non-pilot hospitals), but not in high-resource hospitals (such as pilot hospitals), further study is required to identify the reasons. If countries that have medical service capacity similar to China intend to promote labour neuraxial analgesia, they should be cautious and establish strategies in accordance with their actual conditions.

## Supplementary Information

**Additional file 1.** Supplementary description of methods and results sections.

## Data Availability

The datasets generated and/or analysed during the current study are not publicly available due to the terms of our contract with the Chinese National Health Commission, but are available from the corresponding author on reasonable request.

## Abbreviations

ATT: Treatment effect for the treated cases
CI: Confidence interval
CS: Caesarean section
CSEA: Combined spinal-epidural analgesia
EA: Epidural analgesia
GBD: Global Burden of Disease
ITSA: Interrupted time-series analysis
MNM: Maternal near miss
NMNMSS: National Maternal Near Miss Surveillance System
OLS: ordinary least-squares
PS: Propensity score
PSM: Propensity score matching
RCTs: Randomized controlled trials
RD: Rate difference
RR: Relative risk
SMM: Severe maternal morbidity
UI: Uncertainty interval
WHO: World Health Organization
