# Supplementary material for: The trends and associated adverse maternal and perinatal outcomes of labour neuraxial analgesia among vaginal deliveries in China between 2012 and 2019: a real-world observational evidence
Source: BMC Med. 2021 Mar 19;19:74. doi: 10.1186/s12916-021-01941-6 (PMC7977606; doi:10.1186/s12916-021-01941-6)
Supplement: Supplementary file 1 — Additional file 1. Supplementary description of methods and results sections. [file 12916_2021_1941_MOESM1_ESM.docx]

**Supplementary Online Content**

**Method S1** Estimations of epidural analgesia (EA), combined spinal-epidural analgesia (CSEA) and labour neuraxial analgesia rates at the provincial and national levels

**Method S2** Using interrupted time-series analysis (ITSA) to quantify changes of national labour neuraxial analgesia rates after the national policy on promoting labour analgesia

**Method S3** The details of eight modified Poisson regression models used to describe the association between labour neuraxial analgesia and maternal and perinatal outcomes

**Table S1** The rates and 95% uncertainty intervals (UIs) of provincial labour neuraxial analgesia among vaginal deliveries in China from 2012 to 2019 (%)

**Table S2** The rates and 95% uncertainty intervals (UIs) of provincial epidural analgesia among vaginal deliveries in China from 2012 to 2019 (%)

**Table S3** The rates and 95% uncertainty intervals (UIs) of provincial combined spinal-epidural analgesia among vaginal deliveries in China from 2012 to 2019 (%)

**Table S4** Hospital level distribution of pilot and non-pilot hospitals of the national policy on promoting labour analgesia

**Table S5** Cumby-Huizinga test for autocorrelation in Interrupted time-series analysis

**Table S6** Sensitivity analysis on the difference between pilot and non-pilot hospitals in the slope (post-intervention versus prior-intervention)

**Table S7** Characteristics distribution of women with labour neuraxial analgesia and without any analgesia among vaginal deliveries in 438 hospitals in China

**Fig. S1** Flowchart for the selection of the mothers included in the study from China’s National Maternal Near Miss Surveillance System (2012-2109)

**Fig. S2** Diagnostic plots for the epidural analgesia rate multilevel Bayesian prediction model

**Fig. S3** Diagnostic plots for the combined spinal-epidural analgesia rate multilevel Bayesian prediction model

**Fig. S4** National and provincial observed and Bayesian estimated rates with 95% uncertainty intervals (UIs) on labour neuraxial analgesia, epidural analgesia and combined spinal-epidural analgesia among vaginal deliveries in China from 2012 to 2019

**Fig. S5** Interrupted time-series analysis for single- and multiple-group comparisons on national monthly changes of labor neuraxial analgesia rates after the national policy

**Fig. S6** Comparison of secondary maternal outcomes between women with labour neuraxial analgesia and without any analgesia

**Fig. S7** Comparison of secondary perinatal outcomes between women with labour neuraxial analgesia and without any analgesia

**Fig. S8** The sensitivity analysis in all pregnant women for comparison of maternal and perinatal outcomes between women with labour neuraxial analgesia and without any analgesia

**Fig. S9** Change of bias across covariates after propensity score matching

**Fig. S10** The common support region of propensity scores

**Fig. S11** The E-Values for sensitivity analyses on unobserved confounding factors

**Fig. S12** Conditional Marginal Effects of labour neuraxial analgesia with 95% CIs

**Method S1** Estimations of epidural analgesia (EA), combined spinal-epidural analgesia (CSEA) and labour neuraxial analgesia rates at the provincial and national levels

First, we summarized the individual data from NMNMSS to obtain the observed province-year EA rates. To better represent the provincial level, we obtained the provincial age-specific weights by comparing the number of provincial age-specific live births from the GBD study (assuming that it represents the actual distribution of the population) and NMNMSS and then adjusted the EA rates with these weights. We modelled logit transform of the weighted EA rates using a Bayesian multilevel linear mixed regression model, with correlated random province-specific intercept and time slope. Due to the lack of appropriate published covariates at the provincial level to estimate the EA rates, and because including the time covariate could capture secular trends that might be caused by a range of unmeasured factors, such as the number of anaesthesiologists per 1000 live births at the provincial level, there are no other covariates except time in the model:

Logit(Rate_ij_) = α_j_ + β_j_×Time_i_ + ε_ij_

for the i:th year and the j:th province. The random component α_j_ is the correlated province intercepts, and the random component β_j_ is the time slopes, while ε_ij_ is a normally distributed residual. We used Markov Chain Monte Carlo to simulate the posterior distribution, with a sample size of 10 000 draws, following a burn-in of 10 000 draws. From this procedure, we had 10 000 sets of model parameter values for each province-year observation. To check the prediction accuracy of the model, we divided the data into two groups and randomly selected the data of one year from each province to form a testing data set and the rest as a training data set. The model parameters obtained from the training set were used to predict the data in the testing set. The scatter plots of the predicted mean values and the observed values, and the distribution of the residual were used to confirm that the model had a good prediction ability. Finally, the complete dataset was used for modelling. To estimate EA rates at the provincial level, we generated predictions for all province-years from the estimated model parameters. As a result, we obtained 10 000 sets of EA rates for each province and year. Uncertainty intervals (UIs) were constructed from the 2.5th and 97.5th percentiles of the posterior samples. We performed the same procedure to estimate the CSEA rates. The labour neuraxial analgesia rates were generated by adding the EA and CSEA rates of each province and year. National estimates for each year were generated by weighting provincial estimates according to the number of live births in each province from the GBD study.

**Method S2** Using interrupted time-series analysis (ITSA) to quantify changes of national labour neuraxial analgesia rates after the national policy on promoting labour analgesia

We used interrupted time-series analysis[^21^](#_ENREF_21) with ordinary least-squares (OLS) regression models adjusted for autocorrelation to quantify changes of national labour neuraxial analgesia rates after the national policy on promoting labour analgesia. Monthly data (including labour neuraxial analgesia rates, proportion of maternal age ≥35 years old, women with college education or above, number of antenatal visits ≥7, and women with antepartum complications or medical diseases) were aggregated from the individual data from NMNMSS, weighting for the distribution of provincial age-specific live births, similar to the description above. There were two types of monthly data: one is that all hospitals are aggregated into one data set, and the other is that pilot and non-pilot hospitals are aggregated respectively and recombined into one data set. Both single- and multiple-group comparisons were used to perform interrupted time-series analysis:

| Y_t_ = β_0_ + β_1_T_t_ + β_2_X_t_ + β_3_X_t_T_t_ + ε_t_ | (i) |
| --- | --- |
| Y_t_ = β_0_ + β_1_T_t_ + β_2_X_t_ + β_3_X_t_T_t_ + β_4_Z + β_5_ZT_t_ + β_6_ZX_t_ + β_7_ZX_t_T_t_ + ε_t_ | (ii) |

Yt is the labour neuraxial analgesia rate at each month t, T_t_ is the time since the start of the study (coded as 0-95, January 2012 to December 2019), X_t_ is a dummy variable representing the intervention (coded 0 before January 2019, otherwise 1), and X_t_T_t_ is an interaction term (coded 0 before February 2019 and 1-11 for months from February 2019 to December 2019, respectively). Z is a dummy variable to denote the pilot (treatment group) or non-pilot hospitals (control group), and ZT_t_, ZX_t_, and ZX_t_T_t_ are all interaction terms among previously described variables. In model (i), we looked for significant *p*-values in β_2_ to indicate an immediate policy effect, or in β_3_ to indicate a policy effect over time. In model (ii), we looked for significant *p*-values in β_6_ to indicate the difference between pilot and non-pilot hospitals in the level of the labour neuraxial analgesia rate immediately following introduction of the intervention, or in β_7_ to indicate the difference between pilot and non-pilot groups in the slope (trend) of the labour neuraxial analgesia rate after initiation of the intervention compared with prior-intervention. We estimated the coefficients by OLS regression but produced Newey–West standard errors to handle autocorrelations in addition to possible heteroskedasticity. To ensure that we fitted the model that accounted for the correct autocorrelation structure, we used the Cumby-Huizinga test to identify autocorrelation. We checked the robustness of the policy effect by further adjusting the potential time-varying confounders: proportion of maternal age ≥35 years old, women with college education or above, number of antenatal visits ≥7, and women with antepartum complications or medical diseases.

**Method S3** The details of eight modified Poisson regression models used to describe the association between labour neuraxial analgesia and maternal and perinatal outcomes

We reported crude relative risks (cRRs) and adjusted relative risks (aRRs) with 95% confidence intervals (CIs) of labour neuraxial analgesia for maternal outcomes and perinatal outcomes from eight models using modified Poisson regression with a robust variance estimator and clustering of births within same hospitals. Model A describes the cRRs and 95% CIs of labour neuraxial analgesia for different outcomes. Model B adjusted the effect estimates for potential confounding factors: year, region, hospital level, the number of anaesthesiologists per 1000 births, the number of antenatal care visits, maternal education, marital status, maternal age, gestational age, and parity. For maternal near miss, we further adjusted postpartum complications. From Model C to Model H, we used propensity score (PS) analysis to reduce the observed selection bias in estimating labour neuraxial analgesia risk and to reduce the likelihood of confounding when analysing observational data. We first calculated PS, which estimated the probability of receiving labour neuraxial analgesia using logistic regression with predictors: year, region, hospital level, the number of anaesthesiologists per 1000 births, the number of antenatal care visits, maternal education, marital status, maternal age, gestational age, and parity. All of the PS analyses are restricted to samples that are in the common support region. Model C used a propensity score matching (PSM) approach (4-nearest neighbours matching with a calliper of 0.01). Models D and E used a PS weighting approach. The former used inverse probability of treatment weights (IPTW) for estimating average treatment effect (ATE), and the latter weighed by odds for estimating the average treatment effect for the treated cases (ATT). Models F, G and H used the PS covariate adjustment approach. Model F used PS as the only one covariate and assumed that there was a linear relationship between PS and outcome. Model G was the same as model F, but assumed that there was a non-linear relationship between PS and outcome, selecting a multivariable fractional polynomial model that best predicted the outcome. Model H added all the covariates in model B on the basis of model F to check the robustness of the results. We repeated all of the above models separately in pilot and non-pilot hospitals to determine whether the results were the same in the two groups.

**Table S1** The rates and 95% uncertainty intervals (UIs) of provincial labour neuraxial analgesia among vaginal deliveries in China from 2012 to 2019 (%)

|  | 2012 | | 2013 | | 2014 | | 2015 | | 2016 | | 2017 | | 2018 | | 2019 | |
| --- | --- | --- | --- | --- | --- | --- | --- | --- | --- | --- | --- | --- | --- | --- | --- | --- |
|  | Observed | Bayesian estimate  (95%UI) | Observed | Bayesian estimate  (95%UI) | Observed | Bayesian estimate  (95%UI) | Observed | Bayesian estimate  (95%UI) | Observed | Bayesian estimate  (95%UI) | Observed | Bayesian estimate  (95%UI) | Observed | Bayesian estimate  (95%UI) | Observed | Bayesian estimate  (95%UI) |
| Beijing | 6.1 | 8.6(3.4-17.7) | 10.6 | 9.7(4.0-19.7) | 10.9 | 10.2(4.1-20.2) | 9.6 | 11.6(4.9-22.5) | 11.5 | 12.3(5.1-23.9) | 10.5 | 8.6(3.5-17.4) | 11.3 | 12.9(5.3-25.0) | 18.4 | 18.3(7.9-35.1) |
| Tianjin | 5.4 | 6.7(2.7-13.8) | 6.3 | 6.2(2.5-12.9) | 6.7 | 5.1(2.0-10.8) | 5.8 | 5.0(2.0-10.3) | 4.4 | 4.5(1.8-9.3) | 1.4 | 3.0(1.2-6.3) | 2.4 | 4.1(1.6-8.5) | 9.3 | 5.8(2.3-12.1) |
| Hebei | 7.7 | 10.3(4.1-21.3) | 12.5 | 11.4(4.6-23.4) | 11.7 | 11.4(4.6-23.2) | 11.6 | 12.8(5.2-26.0) | 10.9 | 13.3(5.5-26.8) | 11.1 | 9.6(3.9-19.6) | 12.5 | 14.6(6.0-29.0) | 20.0 | 21.7(9.1-41.5) |
| Shanxi | 3.0 | 4.4(1.7-9.1) | 5.1 | 4.0(1.6-8.4) | 3.8 | 3.4(1.3-7.0) | 3.2 | 3.2(1.2-6.8) | 2.7 | 2.8(1.1-5.9) | 1.1 | 1.6(0.6-3.4) | 1.7 | 2.1(0.8-4.4) | 2.5 | 2.6(1.0-5.6) |
| Inner Mongolia | 7.2 | 15.4(6.4-30.0) | 14.4 | 13.6(5.6-27.3) | 10.0 | 10.9(4.4-21.9) | 18.9 | 10.0(4.0-20.6) | 12.3 | 8.4(3.4-17.1) | 5.9 | 5.4(2.1-11.5) | 4.8 | 7.1(2.9-14.4) | 5.8 | 9.7(3.9-19.5) |
| Liaoning | 26.8 | 32.6(14.7-60.2) | 27.6 | 29.9(13.5-54.4) | 26.5 | 25.0(10.9-47.0) | 27.8 | 23.4(9.9-44.1) | 22.4 | 20.5(8.6-39.7) | 13.9 | 13.4(5.5-26.7) | 12.1 | 17.3(7.3-33.6) | 18.8 | 22.5(9.9-42.6) |
| Jilin | 6.8 | 6.3(2.5-13.0) | 7.4 | 7.9(3.2-16.3) | 7.8 | 9.0(3.6-18.4) | 10.0 | 11.3(4.7-22.4) | 8.2 | 13.3(5.5-26.2) | 11.2 | 10.5(4.2-21.3) | 18.0 | 17.8(7.4-34.4) | 31.0 | 28.5(12.6-53.1) |
| Heilongjiang | 7.9 | 14.8(6.0-29.5) | 9.3 | 11.6(4.6-23.4) | 10.4 | 8.4(3.5-17.2) | 10.9 | 7.1(2.8-14.6) | 8.1 | 5.6(2.2-11.7) | 3.6 | 2.9(1.1-6.1) | 2.1 | 3.4(1.3-7.0) | 2.4 | 3.8(1.5-8.0) |
| Shanghai | 15.6 | 12.4(5.0-25.5) | 19.6 | 14.3(5.9-28.3) | 8.1 | 15.2(6.3-30.2) | 9.0 | 17.8(7.4-34.9) | 13.2 | 19.8(8.4-38.8) | 31.4 | 16.3(6.8-32.5) | 32.6 | 26.3(11.8-48.6) | 36.6 | 41.4(20.1-70.5) |
| Jiangsu | 4.4 | 4.8(1.9-10.2) | 3.7 | 4.6(1.8-9.7) | 3.3 | 3.9(1.5-8.2) | 3.3 | 3.8(1.5-8.0) | 3.0 | 3.4(1.3-7.2) | 2.6 | 2.1(0.8-4.6) | 3.6 | 2.9(1.2-6.1) | 3.2 | 4.1(1.6-8.5) |
| Zhejiang | 7.7 | 9.0(3.7-18.2) | 9.0 | 10.3(4.2-20.0) | 11.3 | 10.6(4.4-21.0) | 12.5 | 12.4(5.1-24.3) | 14.5 | 13.6(5.7-26.3) | 12.3 | 12.0(5.0-23.5) | 19.5 | 19.5(8.5-36.7) | 26.1 | 31.8(15.1-54.6) |
| Anhui | 3.7 | 2.9(1.1-6.3) | 3.7 | 3.8(1.5-7.9) | 3.5 | 4.3(1.7-9.1) | 3.1 | 5.7(2.2-11.9) | 6.4 | 7.0(2.8-14.3) | 6.1 | 6.5(2.6-13.6) | 13.5 | 12.2(5.0-24.3) | 23.2 | 23.1(10.0-43.3) |
| Fujian | 3.2 | 2.1(0.8-4.4) | 2.2 | 2.6(1.0-5.5) | 2.5 | 2.9(1.1-6.1) | 2.8 | 3.7(1.4-7.9) | 2.2 | 4.4(1.7-9.1) | 1.1 | 3.5(1.4-7.4) | 10.4 | 6.1(2.4-12.7) | 21.1 | 10.4(4.2-21.2) |
| Jiangxi | 2.3 | 3.8(1.5-7.9) | 3.8 | 4.8(1.9-10.0) | 6.4 | 5.6(2.3-11.8) | 8.7 | 7.5(3.1-15.3) | 11.8 | 9.5(3.9-19.6) | 10.1 | 8.5(3.4-17.3) | 14.4 | 16.0(6.6-31.6) | 20.8 | 28.6(12.6-53.4) |
| Shandong | 2.2 | 2.6(1.0-5.5) | 4.6 | 2.9(1.1-6.0) | 2.4 | 2.8(1.1-6.1) | 2.0 | 3.1(1.2-6.4) | 2.7 | 3.2(1.2-6.7) | 2.5 | 2.2(0.9-4.7) | 3.9 | 3.4(1.3-7.2) | 4.4 | 5.3(2.1-11.3) |
| Henan | 12.9 | 16.8(7.0-33.1) | 16.0 | 18.0(7.5-34.8) | 19.2 | 17.8(7.5-35.3) | 18.7 | 19.4(8.2-38.1) | 20.0 | 20.2(8.5-39.7) | 17.1 | 15.2(6.2-30.5) | 21.1 | 23.1(9.7-44.3) | 27.6 | 34.3(15.2-62.7) |
| Hubei | 8.9 | 5.9(2.4-12.1) | 6.7 | 6.8(2.7-14.2) | 5.4 | 7.0(2.8-14.2) | 4.0 | 8.3(3.3-17.2) | 4.7 | 9.1(3.7-18.8) | 12.9 | 7.1(2.8-14.7) | 17.5 | 11.6(4.7-23.6) | 12.8 | 19.0(8.1-36.7) |
| Hunan | 16.5 | 15.2(6.1-30.5) | 11.7 | 14.7(6.0-29.5) | 13.0 | 12.8(5.2-25.8) | 13.1 | 12.7(5.2-25.1) | 8.9 | 11.8(4.8-23.5) | 7.3 | 7.9(3.1-16.2) | 10.7 | 10.9(4.4-22.0) | 18.2 | 15.4(6.3-30.6) |
| Guangdong | 2.0 | 2.3(0.9-4.8) | 2.2 | 2.4(0.9-5.1) | 2.3 | 2.2(0.9-4.7) | 2.9 | 2.4(0.9-5.1) | 2.0 | 2.4(0.9-5.0) | 1.4 | 1.6(0.6-3.4) | 2.4 | 2.5(1.0-5.3) | 2.5 | 3.6(1.4-7.6) |
| Guangxi | 5.5 | 6.2(2.5-12.7) | 7.2 | 8.0(3.2-16.1) | 8.4 | 9.3(3.8-18.9) | 11.8 | 12.0(5.0-23.7) | 12.9 | 14.5(6.1-28.0) | 11.8 | 11.3(4.7-22.6) | 19.3 | 18.9(8.3-35.5) | 27.2 | 28.8(13.2-50.7) |
| Hainan | 3.1 | 2.5(0.9-5.2) | 3.0 | 2.7(1.1-5.7) | 2.2 | 2.7(1.0-5.6) | 3.1 | 3.0(1.2-6.4) | 2.5 | 3.2(1.2-6.6) | 0.8 | 2.1(0.8-4.4) | 2.9 | 3.2(1.3-6.6) | 8.1 | 4.6(1.8-9.4) |
| Chongqing | 17.8 | 18.3(7.8-34.9) | 14.3 | 18.5(7.7-35.7) | 15.8 | 17.0(7.3-33.3) | 17.5 | 18.1(7.6-35.5) | 17.6 | 17.8(7.5-34.8) | 12.0 | 13.2(5.4-26.3) | 18.7 | 19.4(8.2-37.6) | 28.2 | 28.2(12.5-52.5) |
| Sichuan | 15.6 | 13.4(5.4-27.1) | 10.7 | 14.1(5.7-28.4) | 11.2 | 13.3(5.4-26.9) | 13.3 | 14.2(5.9-28.6) | 20.1 | 14.3(6.0-28.5) | 9.1 | 10.3(4.3-21.0) | 13.3 | 15.6(6.5-30.7) | 18.2 | 23.5(10.4-44.9) |
| Guizhou | 2.2 | 2.4(0.9-5.1) | 2.5 | 2.8(1.1-5.9) | 2.6 | 2.9(1.1-6.3) | 3.0 | 3.5(1.4-7.4) | 3.7 | 4.0(1.5-8.3) | 2.8 | 3.3(1.3-6.8) | 5.7 | 5.7(2.2-11.9) | 11.7 | 9.9(3.9-20.3) |
| Yunnan | 5.3 | 4.8(1.9-9.9) | 2.7 | 4.5(1.8-9.4) | 2.7 | 3.7(1.5-7.8) | 3.8 | 3.5(1.4-7.3) | 3.0 | 3.1(1.2-6.5) | 2.3 | 1.9(0.7-4.0) | 1.8 | 2.6(1.0-5.5) | 3.6 | 3.4(1.4-7.2) |
| Shaanxi | 14.2 | 19.8(8.2-39.1) | 22.4 | 20.0(8.5-39.6) | 22.0 | 18.2(7.7-35.8) | 16.9 | 18.7(7.8-36.1) | 15.6 | 17.9(7.4-35.2) | 14.1 | 12.4(5.1-25.1) | 18.8 | 17.7(7.5-34.6) | 20.0 | 25.2(11.0-47.6) |
| Gansu | 3.9 | 5.1(2.1-10.8) | 3.7 | 5.7(2.3-11.8) | 9.0 | 5.7(2.3-11.8) | 6.2 | 6.5(2.6-13.5) | 8.3 | 6.7(2.7-13.8) | 7.7 | 4.9(1.9-10.2) | 7.6 | 7.8(3.1-16.1) | 7.6 | 12.5(5.1-25.7) |
| Qinghai | 2.6 | 2.8(1.1-6.0) | 2.3 | 2.6(1.0-5.7) | 2.0 | 2.2(0.9-4.8) | 2.4 | 2.1(0.8-4.4) | 1.8 | 1.8(0.7-3.8) | 0.8 | 1.1(0.4-2.3) | 0.8 | 1.4(0.5-2.9) | 2.8 | 1.8(0.7-3.9) |
| Ningxia | 4.8 | 4.5(1.8-9.5) | 4.1 | 4.3(1.7-9.0) | 2.1 | 3.7(1.5-7.7) | 2.4 | 3.6(1.4-7.4) | 5.1 | 3.2(1.3-6.8) | 3.0 | 1.9(0.7-3.9) | 1.9 | 2.5(1.0-5.2) | 2.2 | 3.1(1.2-6.6) |
| Xinjiang | 3.7 | 4.2(1.7-9.0) | 2.6 | 4.1(1.6-8.6) | 3.5 | 3.6(1.4-7.7) | 5.1 | 3.6(1.4-7.6) | 4.6 | 3.3(1.3-6.9) | 1.1 | 2.1(0.8-4.4) | 4.7 | 2.9(1.1-6.1) | 2.5 | 4.0(1.6-8.3) |

**Table S2** The rates and 95% uncertainty intervals (UIs) of provincial epidural analgesia among vaginal deliveries in China from 2012 to 2019 (%)

|  | 2012 | | 2013 | | 2014 | | 2015 | | 2016 | | 2017 | | 2018 | | 2019 | |
| --- | --- | --- | --- | --- | --- | --- | --- | --- | --- | --- | --- | --- | --- | --- | --- | --- |
|  | Observed | Bayesian estimate  (95%UI) | Observed | Bayesian estimate  (95%UI) | Observed | Bayesian estimate  (95%UI) | Observed | Bayesian estimate  (95%UI) | Observed | Bayesian estimate  (95%UI) | Observed | Bayesian estimate  (95%UI) | Observed | Bayesian estimate  (95%UI) | Observed | Bayesian estimate  (95%UI) |
| Beijing | 0.7 | 1.3(0.5-2.7) | 1.2 | 1.3(0.5-2.8) | 0.9 | 1.2(0.5-2.6) | 1.8 | 1.3(0.5-2.7) | 4.0 | 1.3(0.5-2.7) | 0.5 | 1.0(0.4-2.2) | 0.7 | 1.6(0.6-3.4) | 3.2 | 2.7(1.1-5.7) |
| Tianjin | 3.6 | 5.4(2.2-11.2) | 5.0 | 4.8(1.9-10.0) | 5.5 | 3.7(1.4-7.9) | 4.9 | 3.5(1.4-7.2) | 2.9 | 2.9(1.1-6.1) | 0.7 | 2.0(0.8-4.2) | 1.8 | 2.7(1.1-5.5) | 5.5 | 3.8(1.5-8.1) |
| Hebei | 3.0 | 4.2(1.6-8.8) | 5.0 | 4.4(1.7-9.1) | 4.3 | 4.0(1.6-8.4) | 3.7 | 4.3(1.7-9.0) | 3.0 | 4.3(1.7-9.0) | 4.6 | 3.5(1.4-7.4) | 4.5 | 5.5(2.2-11.2) | 8.6 | 9.1(3.7-17.9) |
| Shanxi | 1.3 | 1.7(0.6-3.6) | 2.0 | 1.4(0.5-2.9) | 0.8 | 1.0(0.4-2.1) | 0.9 | 0.9(0.3-1.9) | 0.5 | 0.7(0.3-1.5) | 0.3 | 0.4(0.2-1.0) | 0.4 | 0.6(0.2-1.2) | 1.0 | 0.8(0.3-1.7) |
| Inner Mongolia | 4.6 | 10.3(4.3-19.9) | 8.8 | 9.3(3.8-18.5) | 6.8 | 7.5(3-15.0) | 13.4 | 7.0(2.8-14.3) | 9.6 | 6.1(2.5-12.3) | 5.1 | 4.3(1.7-9.1) | 4.1 | 5.8(2.3-11.8) | 4.5 | 8.3(3.4-16.6) |
| Liaoning | 17.7 | 20.5(9.3-37.0) | 18.6 | 18.7(8.6-33.4) | 15.2 | 15.5(6.7-29.0) | 16.6 | 14.5(6.2-27.2) | 13.1 | 12.8(5.5-24.6) | 11.0 | 9.3(3.9-18.6) | 8.7 | 12.3(5.2-23.6) | 13.0 | 17.0(7.5-31.6) |
| Jilin | 1.9 | 2.1(0.8-4.4) | 2.3 | 2.4(0.9-5.1) | 1.7 | 2.5(1.0-5.3) | 2.7 | 3.0(1.2-6.2) | 3.0 | 3.3(1.3-6.9) | 4.8 | 3.0(1.2-6.3) | 4.4 | 5.3(2.1-10.8) | 8.3 | 9.7(4.0-19.1) |
| Heilongjiang | 4.9 | 8.0(3.2-16.0) | 4.6 | 5.3(2.1-11.0) | 3.7 | 3.2(1.3-6.7) | 2.8 | 2.2(0.8-4.6) | 2.9 | 1.4(0.5-3.0) | 0.6 | 0.7(0.3-1.5) | 0.3 | 0.7(0.3-1.5) | 0.6 | 0.8(0.3-1.6) |
| Shanghai | 7.6 | 6.8(2.7-13.9) | 10.0 | 8.0(3.3-15.7) | 4.7 | 8.5(3.5-16.8) | 5.3 | 10.2(4.2-19.9) | 7.6 | 11.7(4.9-22.8) | 26.9 | 10.9(4.6-21.4) | 24.0 | 18.2(8.3-33.1) | 20.0 | 30.3(15.2-49.9) |
| Jiangsu | 2.9 | 2.6(1.0-5.6) | 1.8 | 2.4(0.9-5.1) | 1.5 | 2.0(0.8-4.2) | 1.3 | 1.9(0.7-3.9) | 1.3 | 1.6(0.6-3.5) | 1.3 | 1.2(0.4-2.5) | 2.5 | 1.6(0.6-3.5) | 2.0 | 2.5(1.0-5.2) |
| Zhejiang | 6.9 | 8.4(3.5-17.0) | 7.1 | 9.4(3.9-18.3) | 10.7 | 9.6(4.0-18.9) | 11.7 | 11.0(4.5-21.4) | 13.6 | 11.8(5-22.7) | 11.6 | 10.6(4.5-20.7) | 14.7 | 17.0(7.5-31.6) | 21.5 | 27.6(13.3-46.2) |
| Anhui | 1.8 | 1.4(0.5-3.0) | 1.9 | 1.9(0.7-4.0) | 1.9 | 2.3(0.9-4.9) | 2.0 | 3.3(1.3-6.9) | 3.4 | 4.3(1.7-9.0) | 3.8 | 4.7(1.8-9.8) | 10.6 | 9.4(3.8-18.7) | 20.0 | 19.0(8.3-35.1) |
| Fujian | 1.3 | 0.8(0.3-1.8) | 0.7 | 0.9(0.4-2.0) | 0.9 | 1.0(0.4-2.1) | 1.0 | 1.2(0.5-2.6) | 0.5 | 1.3(0.5-2.9) | 0.4 | 1.2(0.5-2.6) | 4.7 | 2.2(0.8-4.7) | 7.2 | 4.2(1.6-8.7) |
| Jiangxi | 1.5 | 2.7(1.1-5.8) | 2.6 | 3.1(1.2-6.5) | 4.2 | 3.3(1.3-6.9) | 4.6 | 3.9(1.6-8.0) | 7.6 | 4.4(1.8-9.2) | 3.1 | 4.0(1.6-8.4) | 5.5 | 7.0(2.8-14.2) | 9.5 | 12.8(5.4-24.4) |
| Shandong | 0.9 | 0.6(0.2-1.3) | 0.6 | 0.7(0.3-1.5) | 0.7 | 0.7(0.3-1.6) | 0.6 | 0.9(0.3-1.8) | 0.6 | 1.0(0.4-2.0) | 0.8 | 0.8(0.3-1.8) | 1.2 | 1.5(0.6-3.1) | 3.1 | 2.7(1.1-5.9) |
| Henan | 7.9 | 11.5(4.8-22.2) | 10.9 | 11.5(4.8-22.1) | 11.4 | 10.6(4.4-20.9) | 11.5 | 10.9(4.6-21.3) | 10.9 | 10.7(4.4-21.1) | 9.0 | 8.6(3.5-17.2) | 12.2 | 12.6(5.3-24.3) | 15.2 | 19.4(8.6-35.4) |
| Hubei | 5.1 | 3.2(1.2-6.6) | 3.3 | 3.6(1.4-7.6) | 2.2 | 3.7(1.5-7.5) | 2.2 | 4.4(1.7-9.2) | 3.0 | 4.9(1.9-10.2) | 7.8 | 4.3(1.7-9.1) | 12.8 | 7.4(3.0-15.1) | 7.8 | 13.2(5.6-25.1) |
| Hunan | 10.3 | 9.2(3.7-18.4) | 8.5 | 8.7(3.6-17.5) | 7.4 | 7.4(3.0-14.9) | 7.1 | 7.3(2.9-14.6) | 3.5 | 6.7(2.7-13.6) | 3.0 | 5.1(2.0-10.4) | 8.1 | 7.1(2.9-14.4) | 15.0 | 10.8(4.4-21.1) |
| Guangdong | 1.0 | 1.3(0.5-2.7) | 1.1 | 1.2(0.4-2.5) | 0.8 | 0.9(0.4-2.0) | 1.3 | 0.9(0.3-1.9) | 0.8 | 0.8(0.3-1.7) | 0.6 | 0.6(0.2-1.2) | 0.5 | 0.8(0.3-1.7) | 1.0 | 1.2(0.5-2.6) |
| Guangxi | 1.2 | 1.1(0.4-2.3) | 1.2 | 1.3(0.5-2.7) | 1.3 | 1.3(0.5-2.7) | 1.3 | 1.5(0.6-3.2) | 1.2 | 1.7(0.6-3.6) | 0.7 | 1.5(0.6-3.2) | 3.4 | 2.6(1.0-5.4) | 5.8 | 4.8(1.9-9.7) |
| Hainan | 0.8 | 0.5(0.2-1.1) | 0.6 | 0.5(0.2-1.1) | 0.3 | 0.5(0.2-1.0) | 0.3 | 0.5(0.2-1.0) | 0.3 | 0.5(0.2-1.0) | 0.1 | 0.4(0.1-0.8) | 1.0 | 0.5(0.2-1.2) | 1.9 | 0.9(0.3-2.0) |
| Chongqing | 13.5 | 14.7(6.3-27.6) | 11.3 | 14.2(5.9-26.9) | 12.6 | 12.4(5.3-23.9) | 12.3 | 12.5(5.3-24.3) | 11.1 | 11.8(5-22.7) | 8.2 | 9.1(3.7-18.0) | 12.7 | 13(5.5-25.0) | 19.0 | 19.1(8.6-34.9) |
| Sichuan | 8.9 | 6.8(2.7-13.8) | 5.2 | 7.3(2.9-14.6) | 5.4 | 6.9(2.7-13.9) | 5.8 | 7.6(3.1-15.2) | 12.0 | 7.8(3.2-15.8) | 6.2 | 6.5(2.7-13.2) | 9.7 | 10.3(4.3-20.2) | 12.3 | 16.9(7.5-31.5) |
| Guizhou | 1.2 | 1.4(0.5-3.0) | 1.6 | 1.7(0.6-3.6) | 1.2 | 1.8(0.7-3.8) | 1.6 | 2.2(0.9-4.6) | 2.9 | 2.5(1.0-5.4) | 2.4 | 2.4(0.9-5.0) | 4.1 | 4.3(1.7-9.0) | 7.7 | 8.0(3.2-16.4) |
| Yunnan | 3.7 | 3.2(1.3-6.6) | 1.7 | 2.8(1.1-5.9) | 1.5 | 2.2(0.9-4.6) | 2.1 | 2.0(0.8-4.1) | 1.5 | 1.6(0.6-3.5) | 1.4 | 1.1(0.4-2.3) | 1.1 | 1.5(0.6-3.1) | 2.0 | 2.1(0.8-4.3) |
| Shaanxi | 7.5 | 10.1(4.2-20.0) | 12.5 | 10.1(4.2-20.2) | 12.5 | 9.1(3.8-18.1) | 6.8 | 9.3(3.9-18.2) | 7.4 | 9.1(3.8-18.1) | 6.4 | 7.2(2.9-14.6) | 8.8 | 10.7(4.5-20.7) | 16.9 | 16.5(7.2-30.8) |
| Gansu | 1.7 | 1.8(0.7-3.9) | 2.0 | 2.1(0.8-4.4) | 2.4 | 2.1(0.8-4.4) | 2.0 | 2.5(1.0-5.1) | 1.0 | 2.7(1.0-5.6) | 4.7 | 2.4(0.9-5.0) | 4.8 | 4.0(1.6-8.5) | 5.0 | 7.4(3.0-15.2) |
| Qinghai | 1.0 | 1.3(0.5-2.7) | 1.0 | 1.1(0.4-2.4) | 1.0 | 0.8(0.3-1.9) | 0.7 | 0.8(0.3-1.6) | 0.5 | 0.6(0.2-1.4) | 0.4 | 0.4(0.2-0.9) | 0.3 | 0.6(0.2-1.2) | 1.4 | 0.8(0.3-1.8) |
| Ningxia | 1.5 | 1.7(0.7-3.5) | 1.0 | 1.5(0.6-3.1) | 0.9 | 1.1(0.4-2.4) | 1.0 | 1.0(0.4-2.1) | 1.3 | 0.8(0.3-1.8) | 0.7 | 0.6(0.2-1.2) | 0.7 | 0.7(0.3-1.6) | 0.5 | 1.0(0.4-2.3) |
| Xinjiang | 1.6 | 1.7(0.7-3.6) | 1.2 | 1.6(0.6-3.4) | 1.4 | 1.4(0.5-2.9) | 1.2 | 1.3(0.5-2.8) | 1.2 | 1.2(0.5-2.6) | 0.6 | 0.9(0.3-1.9) | 1.7 | 1.3(0.5-2.8) | 1.5 | 2.0(0.8-4.2) |

**Table S3** The rates and 95% uncertainty intervals (UIs) of provincial combined spinal-epidural analgesia among vaginal deliveries in China from 2012 to 2019 (%)

|  | 2012 | | 2013 | | 2014 | | 2015 | | 2016 | | 2017 | | 2018 | | 2019 | |
| --- | --- | --- | --- | --- | --- | --- | --- | --- | --- | --- | --- | --- | --- | --- | --- | --- |
|  | Observed | Bayesian estimate  (95%UI) | Observed | Bayesian estimate  (95%UI) | Observed | Bayesian estimate  (95%UI) | Observed | Bayesian estimate  (95%UI) | Observed | Bayesian estimate  (95%UI) | Observed | Bayesian estimate  (95%UI) | Observed | Bayesian estimate  (95%UI) | Observed | Bayesian estimate  (95%UI) |
| Beijing | 5.4 | 7.4(2.9-15.1) | 9.4 | 8.4(3.5-17) | 10.0 | 9.0(3.6-17.7) | 7.8 | 10.3(4.3-20) | 7.5 | 11.0(4.6-21.3) | 9.9 | 7.6(3.1-15.2) | 10.6 | 11.3(4.7-21.9) | 15.2 | 15.6(6.7-29.5) |
| Tianjin | 1.7 | 1.2(0.5-2.6) | 1.3 | 1.4(0.5-2.9) | 1.2 | 1.4(0.5-2.9) | 0.9 | 1.5(0.6-3.3) | 1.5 | 1.6(0.6-3.3) | 0.7 | 1.0(0.4-2.1) | 0.6 | 1.4(0.5-3.1) | 3.8 | 1.9(0.7-4.1) |
| Hebei | 4.7 | 6.1(2.4-12.7) | 7.5 | 7.1(2.8-14.4) | 7.4 | 7.4(3.0-14.9) | 7.9 | 8.5(3.5-17.2) | 7.9 | 9.0(3.7-18.0) | 6.5 | 6.0(2.4-12.4) | 8.0 | 9.1(3.7-18.2) | 11.4 | 12.6(5.3-24.2) |
| Shanxi | 1.8 | 2.7(1.0-5.6) | 3.2 | 2.7(1.0-5.5) | 3.0 | 2.4(0.9-4.9) | 2.3 | 2.3(0.9-4.9) | 2.2 | 2.1(0.8-4.5) | 0.8 | 1.2(0.5-2.5) | 1.3 | 1.5(0.6-3.3) | 1.4 | 1.9(0.7-4.0) |
| Inner Mongolia | 2.6 | 5.1(2.0-10.4) | 5.6 | 4.4(1.7-9.0) | 3.2 | 3.4(1.3-7.1) | 5.5 | 3(1.1-6.3) | 2.7 | 2.4(0.9-4.9) | 0.8 | 1.1(0.4-2.5) | 0.7 | 1.3(0.5-2.8) | 1.3 | 1.4(0.5-3.0) |
| Liaoning | 9.1 | 12.1(5.2-23.4) | 9.0 | 11.2(4.8-21.4) | 11.3 | 9.5(4.0-18.6) | 11.2 | 8.9(3.6-17.3) | 9.2 | 7.6(3.1-15.4) | 2.9 | 4.1(1.6-8.4) | 3.4 | 5.0(2.0-10.2) | 5.7 | 5.6(2.2-11.6) |
| Jilin | 4.9 | 4.2(1.6-8.8) | 5.2 | 5.5(2.2-11.3) | 6.1 | 6.5(2.6-13.4) | 7.4 | 8.3(3.4-16.4) | 5.2 | 9.9(4.1-19.5) | 6.5 | 7.5(3.0-15.2) | 13.7 | 12.5(5.3-24.1) | 22.6 | 18.8(8.5-34.2) |
| Heilongjiang | 3.1 | 6.8(2.7-13.9) | 4.7 | 6.2(2.5-12.7) | 6.7 | 5.2(2.2-10.7) | 8.1 | 4.9(1.9-10.1) | 5.1 | 4.2(1.6-8.7) | 3.0 | 2.2(0.8-4.6) | 1.8 | 2.7(1.0-5.6) | 1.8 | 3.0(1.2-6.4) |
| Shanghai | 8.0 | 5.6(2.2-11.5) | 9.6 | 6.4(2.6-12.9) | 3.5 | 6.7(2.7-13.5) | 3.7 | 7.6(3-15.3) | 5.6 | 8.1(3.4-16.5) | 4.5 | 5.4(2.2-11.1) | 8.6 | 8.1(3.3-16.0) | 16.6 | 11.1(4.7-21.6) |
| Jiangsu | 1.5 | 2.2(0.9-4.7) | 2.0 | 2.2(0.8-4.7) | 1.7 | 1.9(0.7-4.1) | 2.0 | 1.9(0.7-4.1) | 1.7 | 1.8(0.7-3.8) | 1.3 | 1.0(0.4-2.1) | 1.1 | 1.3(0.5-2.8) | 1.3 | 1.6(0.6-3.4) |
| Zhejiang | 0.8 | 0.6(0.2-1.4) | 1.9 | 0.8(0.3-1.8) | 0.6 | 1.1(0.4-2.2) | 0.7 | 1.4(0.5-3.1) | 0.9 | 1.8(0.7-3.8) | 0.7 | 1.4(0.5-2.9) | 4.8 | 2.5(1.0-5.3) | 4.6 | 4.1(1.6-8.7) |
| Anhui | 1.9 | 1.6(0.6-3.3) | 1.8 | 1.9(0.7-4.0) | 1.5 | 2.0(0.8-4.3) | 1.2 | 2.4(0.9-5.2) | 3.0 | 2.7(1.0-5.5) | 2.3 | 1.8(0.7-3.9) | 2.9 | 2.8(1.1-5.9) | 3.2 | 4.1(1.6-8.7) |
| Fujian | 1.9 | 1.2(0.5-2.7) | 1.5 | 1.6(0.6-3.5) | 1.5 | 1.9(0.7-4.1) | 1.8 | 2.5(1-5.4) | 1.7 | 3.0(1.2-6.3) | 0.7 | 2.3(0.9-4.8) | 5.7 | 3.9(1.6-8.1) | 13.8 | 6.2(2.5-12.7) |
| Jiangxi | 0.8 | 1.1(0.4-2.3) | 1.2 | 1.7(0.6-3.5) | 2.2 | 2.3(0.9-5.0) | 4.1 | 3.6(1.4-7.4) | 4.2 | 5.1(2.0-10.6) | 7.0 | 4.5(1.8-9.2) | 8.9 | 8.9(3.7-17.7) | 11.4 | 15.8(7.0-29.5) |
| Shandong | 1.3 | 2.0(0.8-4.3) | 3.9 | 2.1(0.8-4.5) | 1.7 | 2.1(0.8-4.5) | 1.4 | 2.2(0.9-4.7) | 2.1 | 2.2(0.9-4.7) | 1.7 | 1.4(0.5-2.9) | 2.7 | 1.9(0.7-4.1) | 1.3 | 2.5(1.0-5.5) |
| Henan | 5.0 | 5.4(2.1-11.1) | 5.1 | 6.5(2.6-13.0) | 7.8 | 7.2(2.9-14.6) | 7.3 | 8.5(3.5-17) | 9.2 | 9.5(3.9-18.8) | 8.1 | 6.7(2.7-13.6) | 8.9 | 10.5(4.3-20.4) | 12.3 | 14.9(6.4-28.0) |
| Hubei | 3.8 | 2.7(1.1-5.7) | 3.3 | 3.2(1.3-6.7) | 3.2 | 3.3(1.3-6.8) | 1.9 | 3.9(1.5-8.1) | 1.7 | 4.2(1.7-8.8) | 5.2 | 2.7(1.1-5.8) | 4.7 | 4.2(1.6-8.7) | 5.0 | 5.9(2.4-12.0) |
| Hunan | 6.2 | 6.0(2.3-12.3) | 3.2 | 6.0(2.4-12.3) | 5.6 | 5.4(2.1-11.0) | 6.0 | 5.4(2.1-10.9) | 5.4 | 5.0(2.0-10.2) | 4.3 | 2.9(1.1-6.0) | 2.6 | 3.8(1.5-7.8) | 3.2 | 4.6(1.8-9.6) |
| Guangdong | 1.0 | 1.0(0.4-2.2) | 1.1 | 1.2(0.5-2.7) | 1.6 | 1.3(0.5-2.8) | 1.6 | 1.5(0.6-3.2) | 1.3 | 1.6(0.6-3.4) | 0.7 | 1.0(0.4-2.2) | 1.9 | 1.7(0.7-3.5) | 1.5 | 2.4(0.9-5.0) |
| Guangxi | 4.3 | 5.1(2.0-10.5) | 6.0 | 6.7(2.7-13.5) | 7.1 | 8.0(3.2-16.3) | 10.5 | 10.5(4.4-20.6) | 11.7 | 12.8(5.4-24.7) | 11.0 | 9.8(4.1-19.5) | 15.9 | 16.3(7.2-30.2) | 21.3 | 24.0(11.2-41.5) |
| Hainan | 2.3 | 1.9(0.7-4.2) | 2.4 | 2.2(0.8-4.6) | 1.9 | 2.3(0.9-4.7) | 2.8 | 2.6(1-5.4) | 2.2 | 2.7(1.0-5.7) | 0.7 | 1.7(0.7-3.7) | 1.9 | 2.6(1.0-5.4) | 6.2 | 3.6(1.4-7.6) |
| Chongqing | 4.3 | 3.7(1.4-7.7) | 3.0 | 4.3(1.7-9.1) | 3.2 | 4.6(1.8-9.6) | 5.2 | 5.5(2.2-11.3) | 6.5 | 6.0(2.4-12.3) | 3.7 | 4.1(1.6-8.5) | 6.1 | 6.4(2.5-13.0) | 9.2 | 9.1(3.7-18.2) |
| Sichuan | 6.8 | 6.6(2.6-13.5) | 5.5 | 6.9(2.7-13.9) | 5.9 | 6.4(2.6-13.1) | 7.5 | 6.7(2.7-13.5) | 8.2 | 6.5(2.6-13.1) | 2.9 | 3.8(1.5-7.9) | 3.6 | 5.3(2.1-10.8) | 6.0 | 6.6(2.7-13.6) |
| Guizhou | 1.0 | 1.0(0.4-2.1) | 0.9 | 1.1(0.4-2.4) | 1.3 | 1.2(0.4-2.5) | 1.4 | 1.3(0.5-2.8) | 0.8 | 1.4(0.5-3.0) | 0.3 | 0.9(0.3-1.9) | 1.6 | 1.4(0.5-3.0) | 4.0 | 1.9(0.7-4.1) |
| Yunnan | 1.6 | 1.6(0.6-3.4) | 1.0 | 1.6(0.6-3.5) | 1.1 | 1.5(0.6-3.3) | 1.7 | 1.5(0.6-3.2) | 1.6 | 1.4(0.6-3.1) | 1.0 | 0.8(0.3-1.8) | 0.7 | 1.1(0.4-2.4) | 1.6 | 1.4(0.5-2.9) |
| Shaanxi | 6.7 | 9.7(3.9-19.5) | 9.8 | 9.9(4.1-20.0) | 9.5 | 9.2(3.8-18.1) | 10.1 | 9.3(3.8-18.1) | 8.1 | 8.8(3.6-17.3) | 7.7 | 5.2(2.1-10.8) | 10.1 | 7.0(2.9-14.2) | 3.1 | 8.7(3.6-17.4) |
| Gansu | 2.2 | 3.3(1.3-7.0) | 1.7 | 3.6(1.4-7.5) | 6.6 | 3.6(1.4-7.6) | 4.3 | 4(1.6-8.4) | 7.3 | 4.1(1.6-8.4) | 3.0 | 2.5(1.0-5.3) | 2.8 | 3.8(1.5-7.8) | 2.6 | 5.1(2.0-10.8) |
| Qinghai | 1.6 | 1.6(0.6-3.4) | 1.2 | 1.5(0.6-3.4) | 1.0 | 1.4(0.5-2.9) | 1.7 | 1.3(0.5-2.8) | 1.2 | 1.2(0.5-2.5) | 0.4 | 0.6(0.2-1.4) | 0.5 | 0.8(0.3-1.8) | 1.4 | 1.0(0.4-2.1) |
| Ningxia | 3.2 | 2.9(1.1-6.1) | 3.1 | 2.9(1.1-6.0) | 1.2 | 2.6(1.0-5.4) | 1.4 | 2.6(1-5.3) | 3.8 | 2.4(0.9-5.0) | 2.3 | 1.3(0.5-2.8) | 1.2 | 1.7(0.7-3.7) | 1.7 | 2.1(0.8-4.4) |
| Xinjiang | 2.0 | 2.5(1.0-5.4) | 1.4 | 2.5(1.0-5.3) | 2.1 | 2.3(0.9-4.9) | 3.9 | 2.3(0.9-4.8) | 3.3 | 2.1(0.8-4.5) | 0.5 | 1.2(0.4-2.5) | 3.0 | 1.6(0.6-3.4) | 1.0 | 2.0(0.8-4.2) |

**Table S4** Hospital level distribution of pilot and non-pilot hospitals of the national policy on promoting labour analgesia

|  | Level 1 or unknown level hospitals | | Level 2 hospitals | | Level 3 hospitals | | Total | |
| --- | --- | --- | --- | --- | --- | --- | --- | --- |
|  | Number | % | Number | % | Number | % | Number | % |
| Pilot hospitals | 12 | 9.6 | 38 | 30.4 | 75 | 60.0 | 125 | 100.0 |
| Non-pilot hospitals | 50 | 16.0 | 196 | 62.6 | 67 | 21.4 | 313 | 100.0 |
| Total | 62 | 14.2 | 234 | 53.4 | 142 | 32.4 | 438 | 100.0 |

**Table S5** Cumby-Huizinga test for autocorrelation in Interrupted time-series analysis

|  | | lag | chi2 | df | *P*-value |
| --- | --- | --- | --- | --- | --- |
| Single-group(All hospitals) | Crude model | 1 | 61.369 | 1 | <0.0001 |
|  |  | 2 | 16.767 | 1 | <0.0001 |
|  |  | 3 | 8.595 | 1 | 0.0034 |
|  |  | 4 | 5.262 | 1 | 0.0218 |
|  |  | 5 | 2.945 | 1 | 0.0861 |
|  |  | 6 | 1.537 | 1 | 0.2151 |
|  |  | 7 | 0.386 | 1 | 0.5345 |
|  |  | 8 | 0.047 | 1 | 0.8278 |
|  |  | 9 | 0.055 | 1 | 0.8146 |
|  |  | 10 | 0.023 | 1 | 0.8802 |
|  |  | 11 | 0.291 | 1 | 0.5896 |
|  |  | 12 | 1.318 | 1 | 0.2510 |
|  | Adjusted model | 1 | 51.951 | 1 | <0.0001 |
|  |  | 2 | 15.303 | 1 | 0.0001 |
|  |  | 3 | 6.072 | 1 | 0.0137 |
|  |  | 4 | 3.855 | 1 | 0.0496 |
|  |  | 5 | 1.500 | 1 | 0.2207 |
|  |  | 6 | 0.995 | 1 | 0.3185 |
|  |  | 7 | 0.513 | 1 | 0.4740 |
|  |  | 8 | 1.267 | 1 | 0.2603 |
|  |  | 9 | 0.254 | 1 | 0.6146 |
|  |  | 10 | 1.366 | 1 | 0.2425 |
|  |  | 11 | 1.701 | 1 | 0.1921 |
|  |  | 12 | 1.220 | 1 | 0.2694 |
| Single-group(pilot hospitals) | Crude model | 1 | 54.403 | 1 | <0.0001 |
|  |  | 2 | 12.806 | 1 | 0.0003 |
|  |  | 3 | 6.164 | 1 | 0.0130 |
|  |  | 4 | 4.822 | 1 | 0.0281 |
|  |  | 5 | 4.650 | 1 | 0.0310 |
|  |  | 6 | 3.639 | 1 | 0.0565 |
|  |  | 7 | 1.620 | 1 | 0.2031 |
|  |  | 8 | 0.786 | 1 | 0.3753 |
|  |  | 9 | 0.164 | 1 | 0.6860 |
|  |  | 10 | 0.338 | 1 | 0.5611 |
|  |  | 11 | 0.158 | 1 | 0.6908 |
|  |  | 12 | 0.001 | 1 | 0.9781 |
|  | Adjusted model | 1 | 49.432 | 1 | <0.0001 |
|  |  | 2 | 12.154 | 1 | 0.0005 |
|  |  | 3 | 7.632 | 1 | 0.0057 |
|  |  | 4 | 5.021 | 1 | 0.0250 |
|  |  | 5 | 3.154 | 1 | 0.0758 |
|  |  | 6 | 2.346 | 1 | 0.1256 |
|  |  | 7 | 1.351 | 1 | 0.2452 |
|  |  | 8 | 1.070 | 1 | 0.3010 |
|  |  | 9 | 0.391 | 1 | 0.5316 |
|  |  | 10 | 0.332 | 1 | 0.5643 |
|  |  | 11 | 0.148 | 1 | 0.7002 |
|  |  | 12 | 0.155 | 1 | 0.6938 |
| Multiple-group(Pilot and non-pilot hospitals) | Crude model | 1 | 112.530 | 1 | <0.0001 |
|  |  | 2 | 29.996 | 1 | <0.0001 |
|  |  | 3 | 14.855 | 1 | 0.0001 |
|  |  | 4 | 10.643 | 1 | 0.0011 |
|  |  | 5 | 7.691 | 1 | 0.0055 |
|  |  | 6 | 5.276 | 1 | 0.0216 |
|  |  | 7 | 2.178 | 1 | 0.1400 |
|  |  | 8 | 0.858 | 1 | 0.3543 |
|  |  | 9 | 0.077 | 1 | 0.7808 |
|  |  | 10 | 0.093 | 1 | 0.7599 |
|  |  | 11 | 0.009 | 1 | 0.9251 |
|  |  | 12 | 0.461 | 1 | 0.4972 |
|  | Adjusted model | 1 | 105.387 | 1 | <0.0001 |
|  |  | 2 | 29.216 | 1 | <0.0001 |
|  |  | 3 | 14.767 | 1 | 0.0001 |
|  |  | 4 | 9.312 | 1 | 0.0023 |
|  |  | 5 | 5.224 | 1 | 0.0223 |
|  |  | 6 | 3.287 | 1 | 0.0698 |
|  |  | 7 | 1.526 | 1 | 0.2167 |
|  |  | 8 | 0.911 | 1 | 0.3399 |
|  |  | 9 | 0.112 | 1 | 0.7380 |
|  |  | 10 | 0.124 | 1 | 0.7252 |
|  |  | 11 | 0.046 | 1 | 0.8293 |
|  |  | 12 | 0.000 | 1 | 0.9955 |

**Table S6** Sensitivity analysis on the difference between pilot and non-pilot hospitals in the slope (post-intervention versus prior-intervention)

| Change the starting time of intervention | *β*(Coefficient) | *P*-value | 95% CI | |
| --- | --- | --- | --- | --- |
|  |  |  | Lower | Upper |
| Dec 2018 | 0.18 | .019 | 0.03 | 0.33 |
| Nov 2018 | 0.18 | .008 | 0.05 | 0.31 |
| Oct 2018 | 0.15 | .019 | 0.03 | 0.28 |
| Sep 2018 | 0.13 | .048 | 0.00 | 0.27 |
| Aug 2018 | 0.11 | .149 | -0.04 | 0.25 |
| Jul 2018 | 0.09 | .236 | -0.06 | 0.23 |

**Table S7** Characteristics distribution of women with labour neuraxial analgesia and without any analgesia among vaginal deliveries in 438 hospitals in China

|  | Labour neuraxial analgesia | | | | Total | |
| --- | --- | --- | --- | --- | --- | --- |
|  | No | | Yes | |  |  |
|  | Number | % | Number | % | Number | % |
| Year | | | | | | |
| 2012 | 624829 | 11.6 | 53571 | 8.6 | 678400 | 11.3 |
| 2013 | 599061 | 11.1 | 58373 | 9.4 | 657434 | 10.9 |
| 2014 | 715900 | 13.3 | 71560 | 11.5 | 787460 | 13.1 |
| 2015 | 652671 | 12.1 | 67958 | 10.9 | 720629 | 12.0 |
| 2016 | 824840 | 15.3 | 88582 | 14.3 | 913422 | 15.2 |
| 2017 | 693395 | 12.8 | 66479 | 10.7 | 759874 | 12.6 |
| 2018 | 658021 | 12.2 | 88537 | 14.3 | 746558 | 12.4 |
| 2019 | 633478 | 11.7 | 125791 | 20.3 | 759269 | 12.6 |
| Region | | | | | | |
| East-rural | 643943 | 11.9 | 31599 | 5.1 | 675542 | 11.2 |
| East-urban | 927821 | 17.2 | 135025 | 21.7 | 1062846 | 17.6 |
| Central-rural | 1021861 | 18.9 | 90382 | 14.6 | 1112243 | 18.5 |
| Central-urban | 983410 | 18.2 | 168471 | 27.1 | 1151881 | 19.1 |
| West-rural | 785776 | 14.5 | 38479 | 6.2 | 824255 | 13.7 |
| West-urban | 1039384 | 19.2 | 156895 | 25.3 | 1196279 | 19.9 |
| Hospital level | | | | | | |
| Unknown | 303134 | 5.6 | 31814 | 5.1 | 334948 | 5.6 |
| Level 1 | 332240 | 6.2 | 60256 | 9.7 | 392496 | 6.5 |
| Level 2 | 2618760 | 48.5 | 248235 | 40.0 | 2866995 | 47.6 |
| Level 3 | 2148061 | 39.8 | 280546 | 45.2 | 2428607 | 40.3 |
| Number of anaesthesiologists per 1000 births, median (interquartile range) | 2(1-5) | | | | | |
| Antenatal care (number of visits) | | | | | | |
| None | 66919 | 1.2 | 8810 | 1.4 | 75729 | 1.3 |
| 1-3 | 398290 | 7.4 | 25566 | 4.1 | 423856 | 7.0 |
| 4-6 | 1624966 | 30.1 | 122231 | 19.7 | 1747197 | 29.0 |
| 7-9 | 1574266 | 29.1 | 180003 | 29.0 | 1754269 | 29.1 |
| >=10 | 1587346 | 29.4 | 267605 | 43.1 | 1854951 | 30.8 |
| Missing | 150408 | 2.8 | 16636 | 2.7 | 167044 | 2.8 |
| Maternal education | | | | | | |
| Illiterate | 30805 | 0.6 | 1204 | 0.2 | 32009 | 0.5 |
| Primary school | 179167 | 3.3 | 7407 | 1.2 | 186574 | 3.1 |
| Middle school | 1835408 | 34.0 | 125386 | 20.2 | 1960794 | 32.6 |
| High school | 1400458 | 25.9 | 148360 | 23.9 | 1548818 | 25.7 |
| College or higher | 1850376 | 34.3 | 314110 | 50.6 | 2164486 | 35.9 |
| Missing | 105981 | 2.0 | 24384 | 3.9 | 130365 | 2.2 |
| Marital status | | | | | | |
| Single, widowed or divorced | 93854 | 1.7 | 9232 | 1.5 | 103086 | 1.7 |
| Married | 5307431 | 98.2 | 611488 | 98.5 | 5918919 | 98.3 |
| Missing | 910 | 0.0 | 131 | 0.0 | 1041 | 0.0 |
| Maternal age (years) | | | | | | |
| <20 | 142103 | 2.6 | 9267 | 1.5 | 151370 | 2.5 |
| 20-24 | 1071307 | 19.8 | 101839 | 16.4 | 1173146 | 19.5 |
| 25-29 | 2298447 | 42.5 | 291629 | 47.0 | 2590076 | 43.0 |
| 30-34 | 1257475 | 23.3 | 155598 | 25.1 | 1413073 | 23.5 |
| 35-39 | 397627 | 7.4 | 40782 | 6.6 | 438409 | 7.3 |
| >=40 | 86323 | 1.6 | 8087 | 1.3 | 94410 | 1.6 |
| Missing | 148913 | 2.8 | 13649 | 2.2 | 162562 | 2.7 |
| Gestational age(weeks) | | | | | | |
| 28-33 | 101866 | 1.9 | 7060 | 1.1 | 108926 | 1.8 |
| 34-36 | 218782 | 4.0 | 20217 | 3.3 | 238999 | 4.0 |
| 37-41 | 5043039 | 93.4 | 591177 | 95.2 | 5634216 | 93.5 |
| >=42 | 38508 | 0.7 | 2397 | 0.4 | 40905 | 0.7 |
| Parity | | | | | | |
| Nulliparous | 2986615 | 55.3 | 440580 | 71.0 | 3427195 | 56.9 |
| Parous without prior caesarean section | 2279223 | 42.2 | 158054 | 25.5 | 2437277 | 40.5 |
| Parous with prior caesarean section | 127789 | 2.4 | 21891 | 3.5 | 149680 | 2.5 |
| Missing | 8568 | 0.2 | 326 | 0.1 | 8894 | 0.1 |
| Total | 5402195 | 100.0 | 620851 | 100.0 | 6023046 | 100.0 |

**Fig. S1** Flowchart for the selection of the mothers included in the study from China’s National Maternal Near Miss Surveillance System (2012-2109)

**Fig. S2** Diagnostic plots for the epidural analgesia rate multilevel Bayesian prediction model


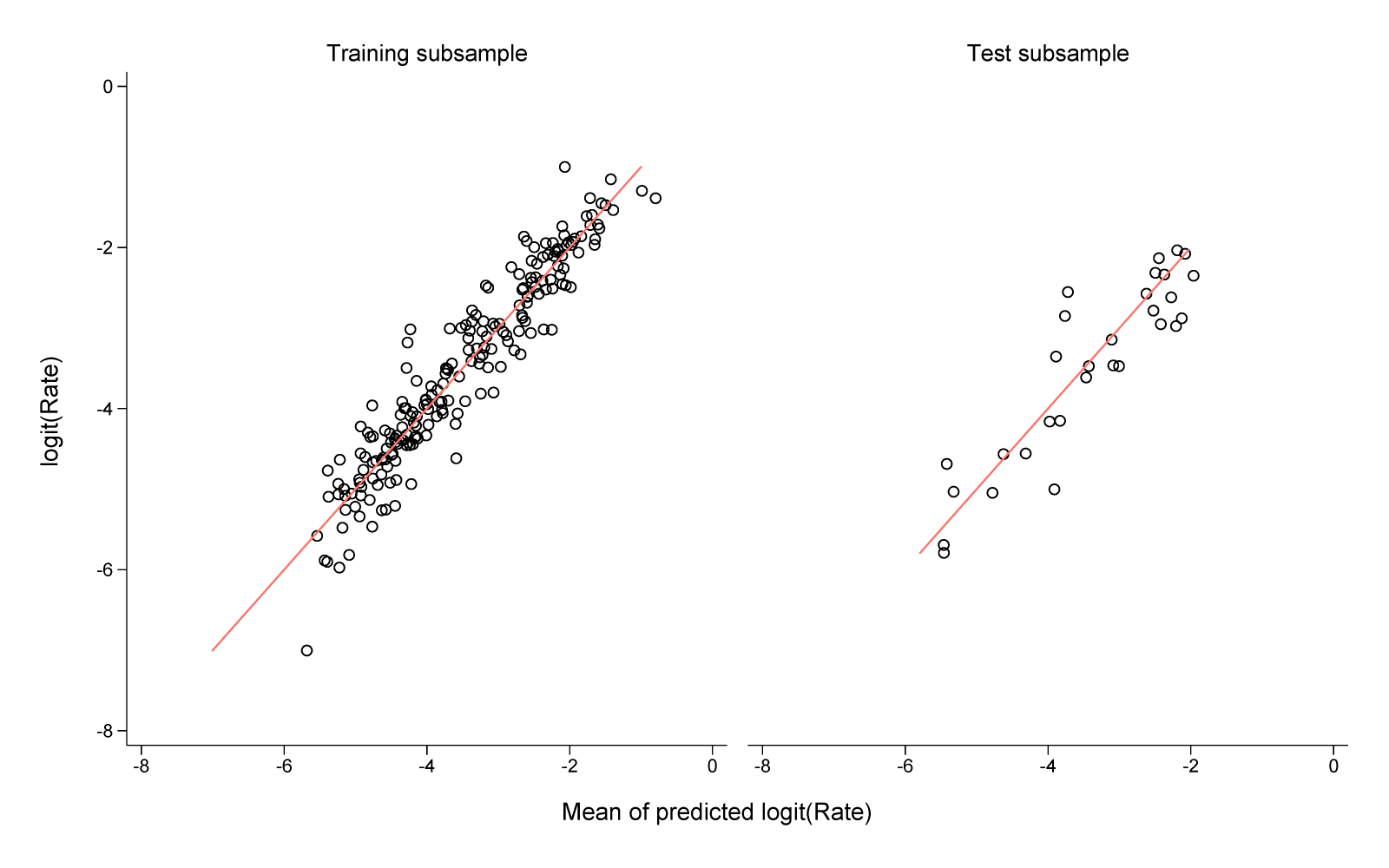

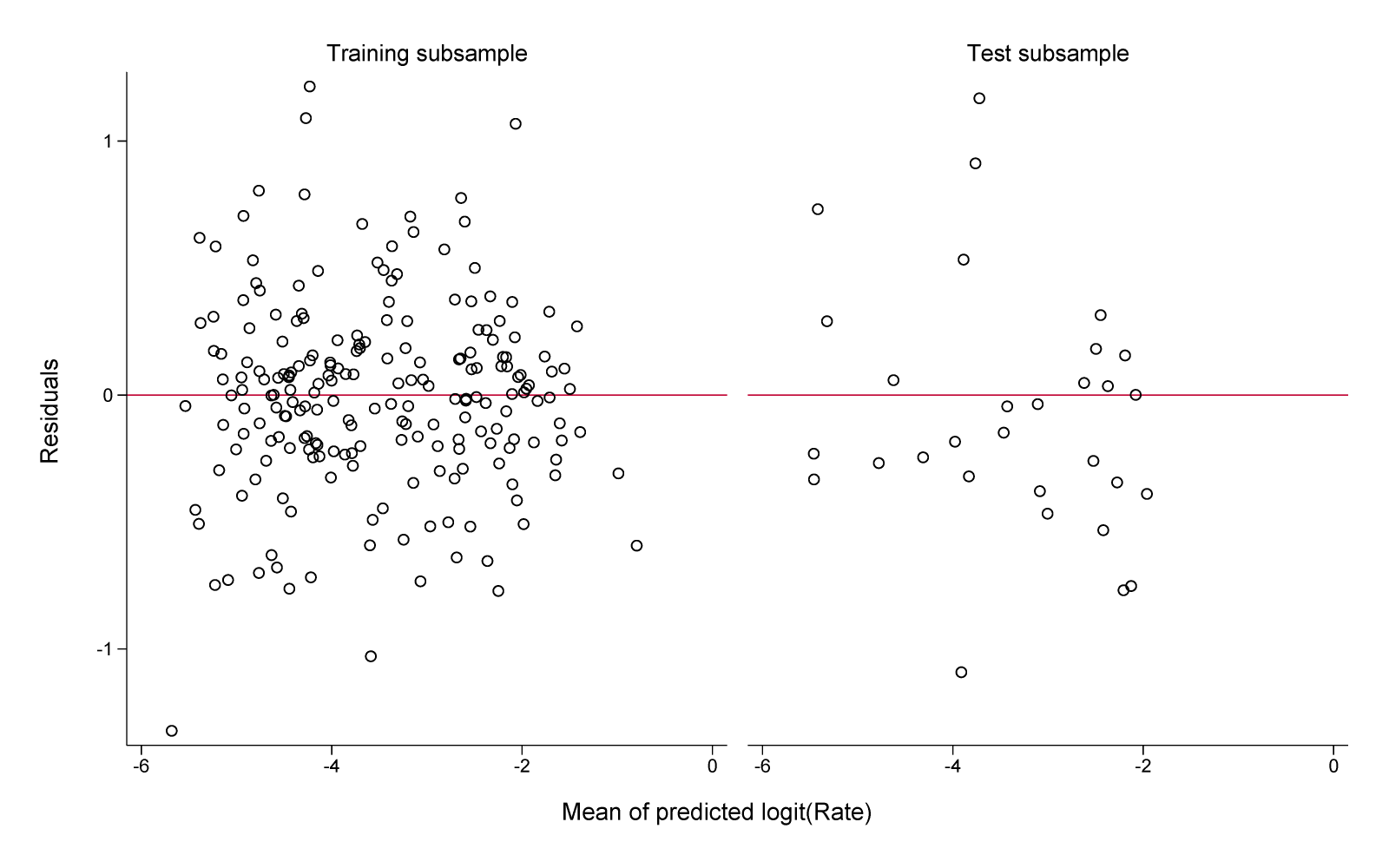


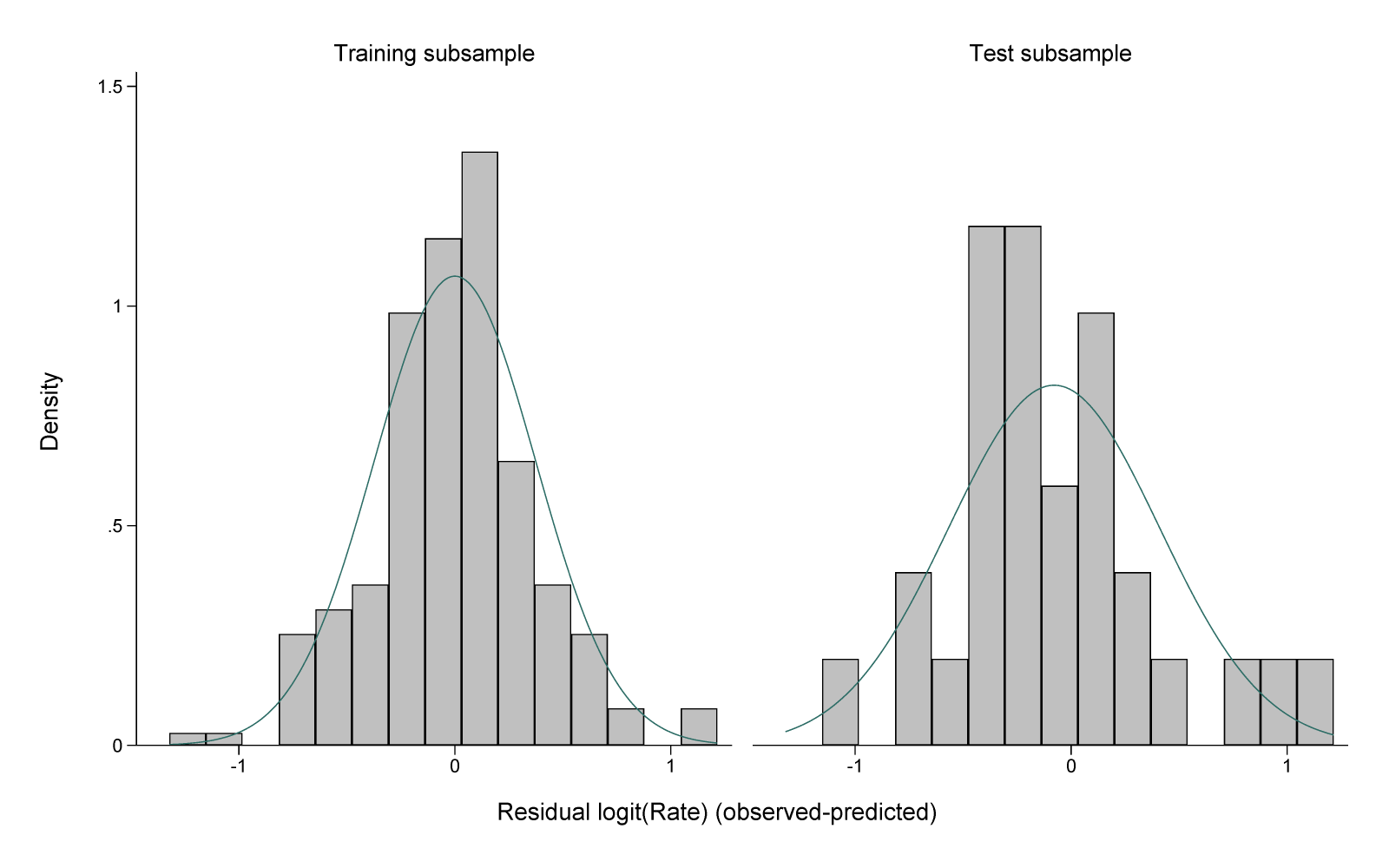


**Fig. S3** Diagnostic plots for the combined spinal-epidural analgesia rate multilevel Bayesian prediction model


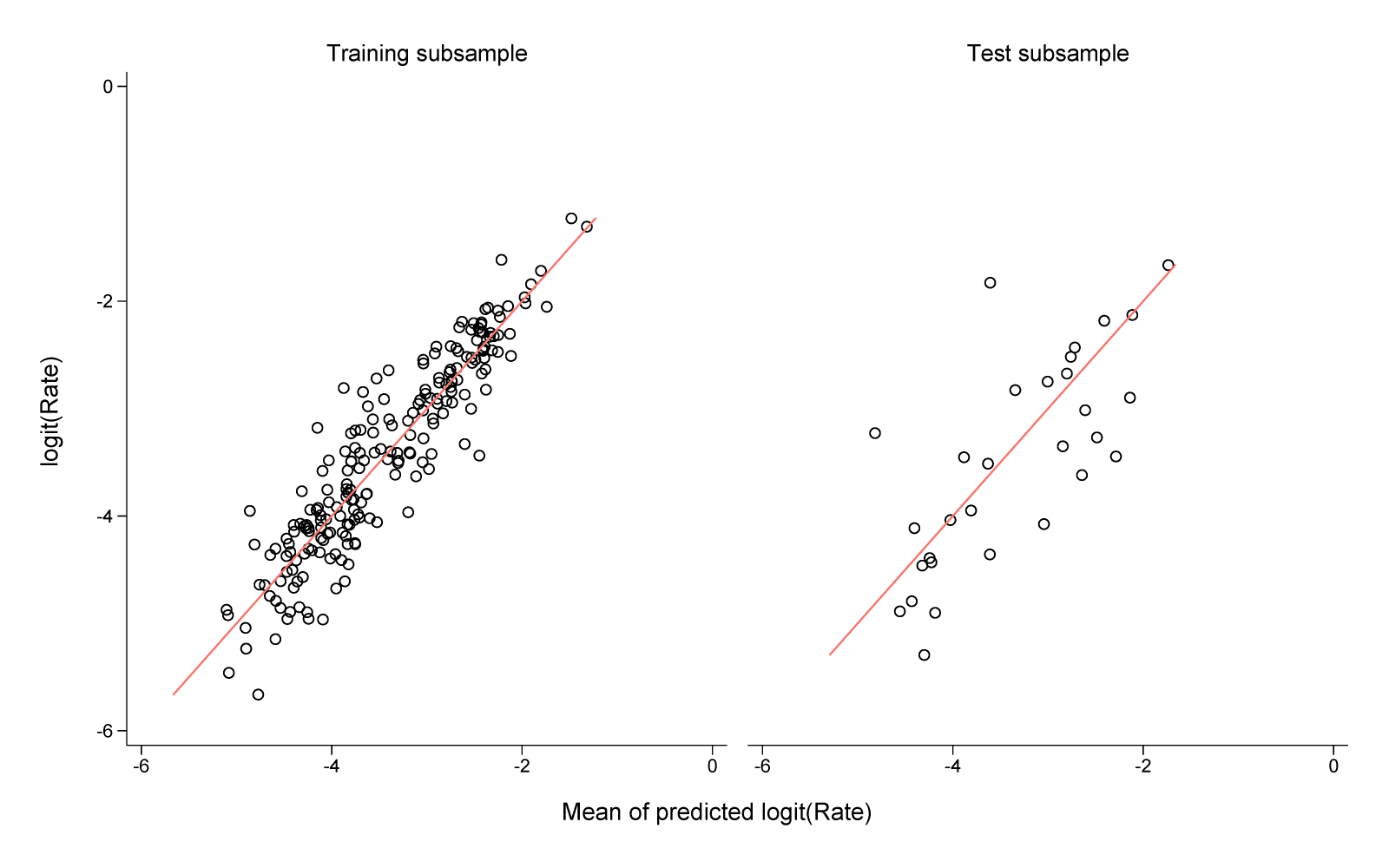

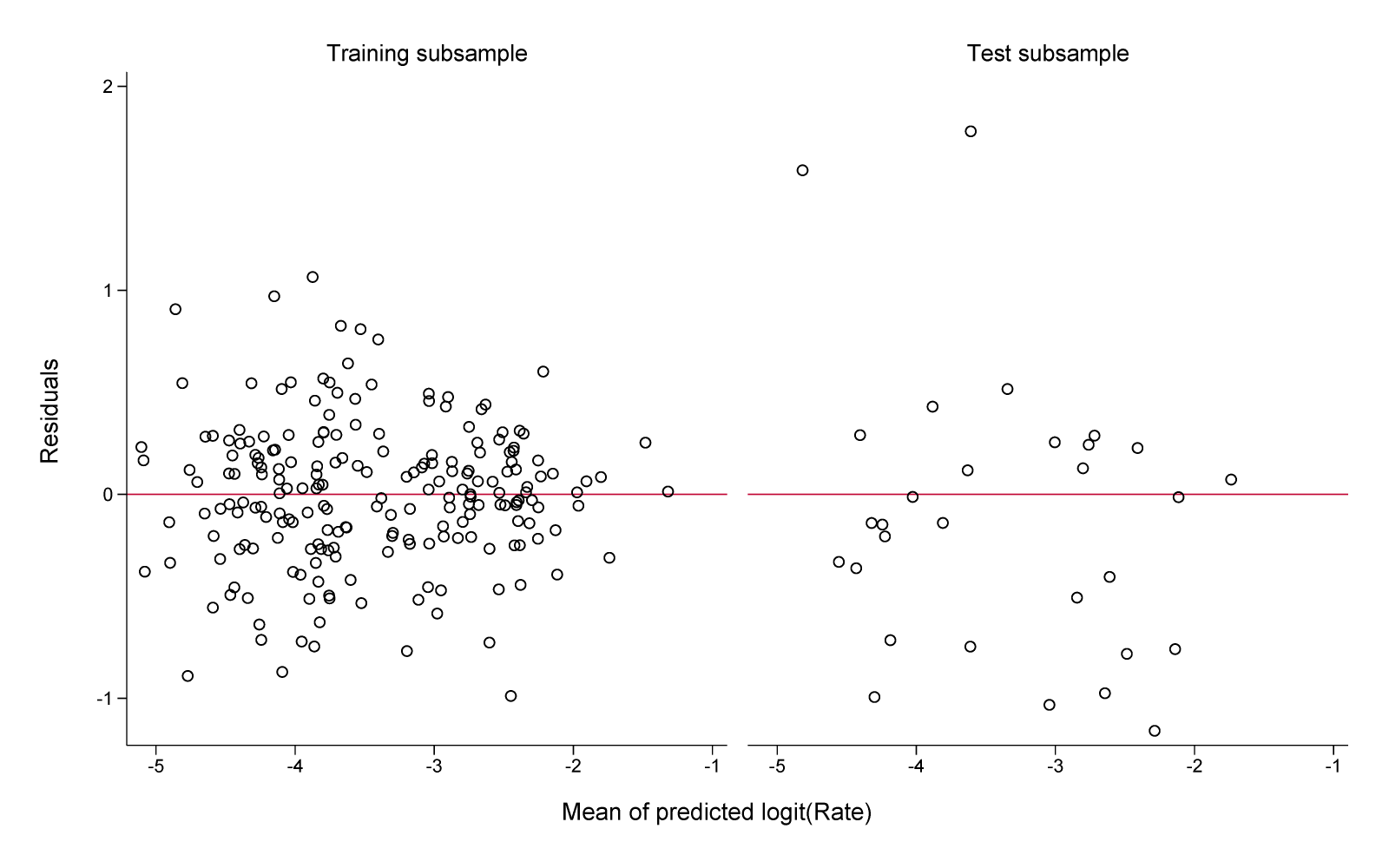

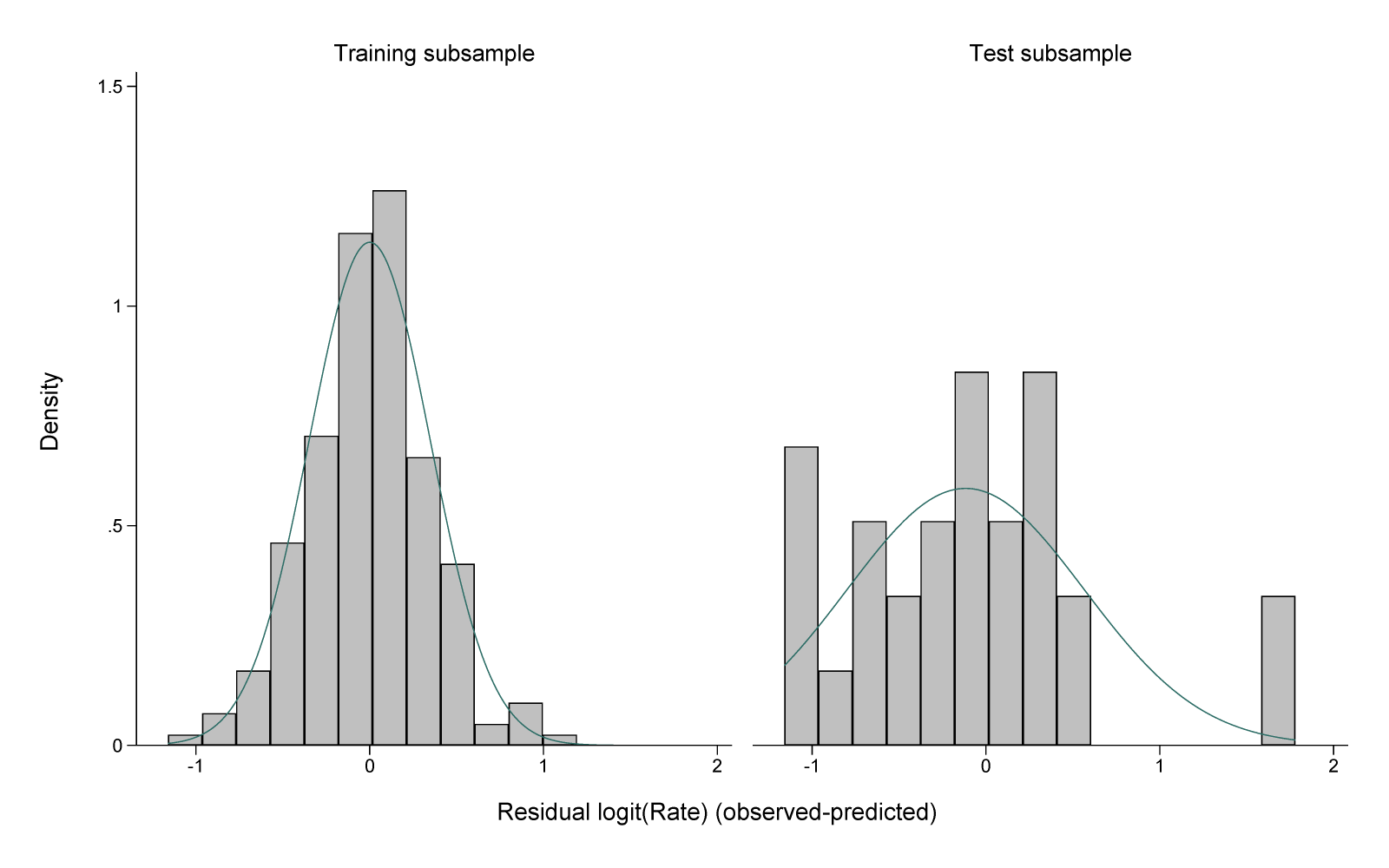


**Fig. S4** National and provincial observed and Bayesian estimated rates (with 95% UI) on labour neuraxial analgesia, epidural analgesia and combined spinal-epidural analgesia among vaginal deliveries in China from 2012 to 2019


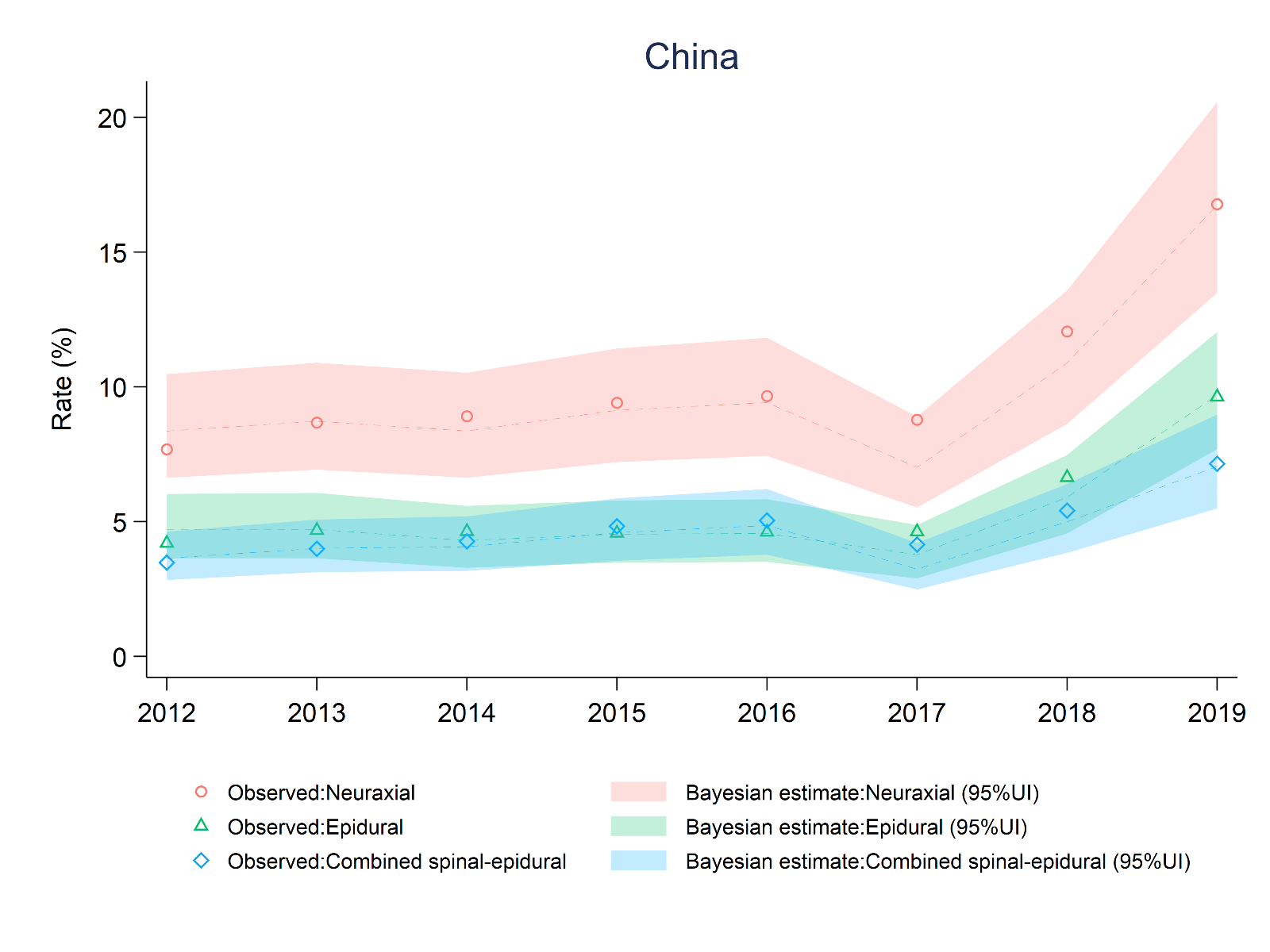


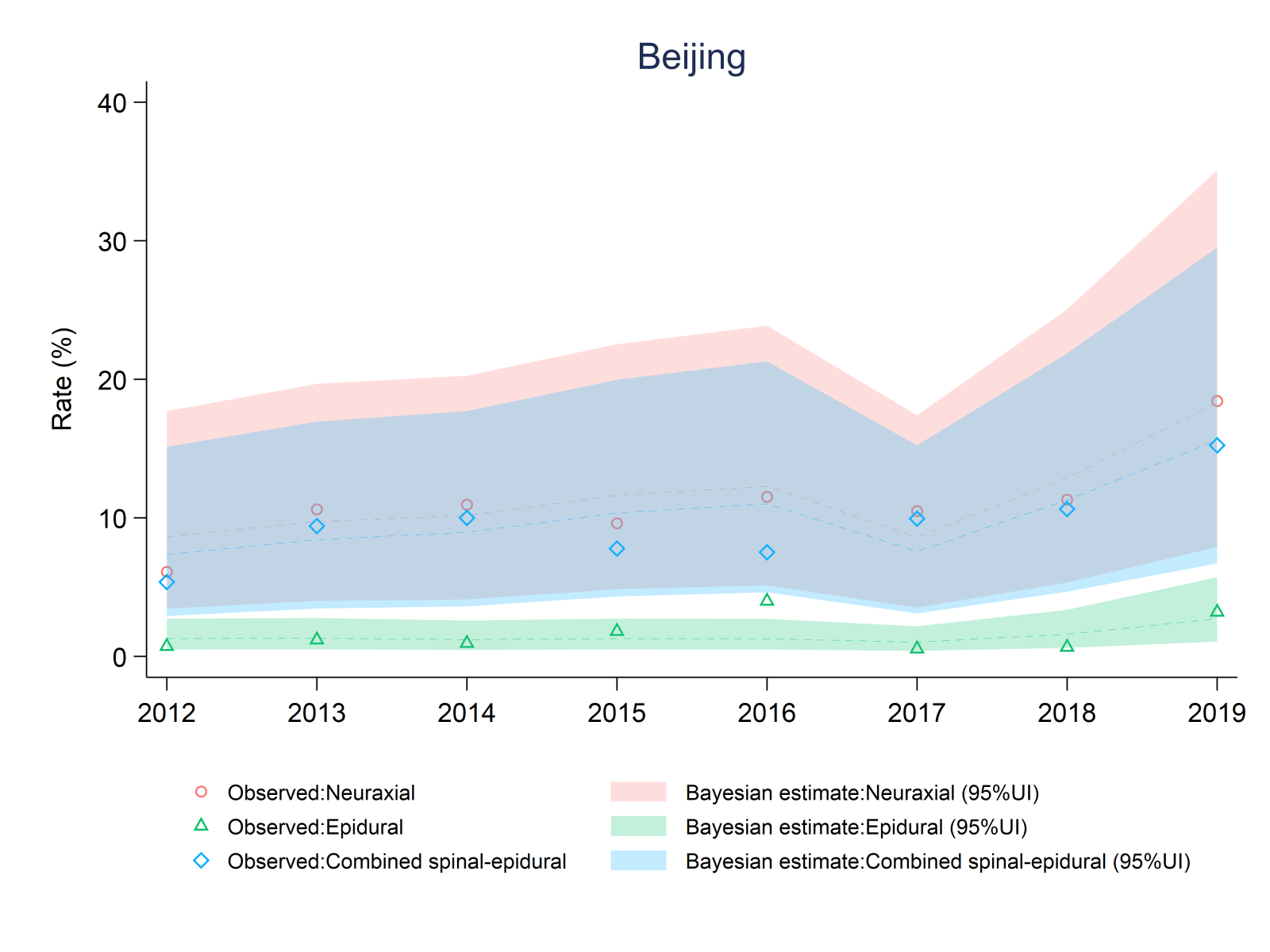


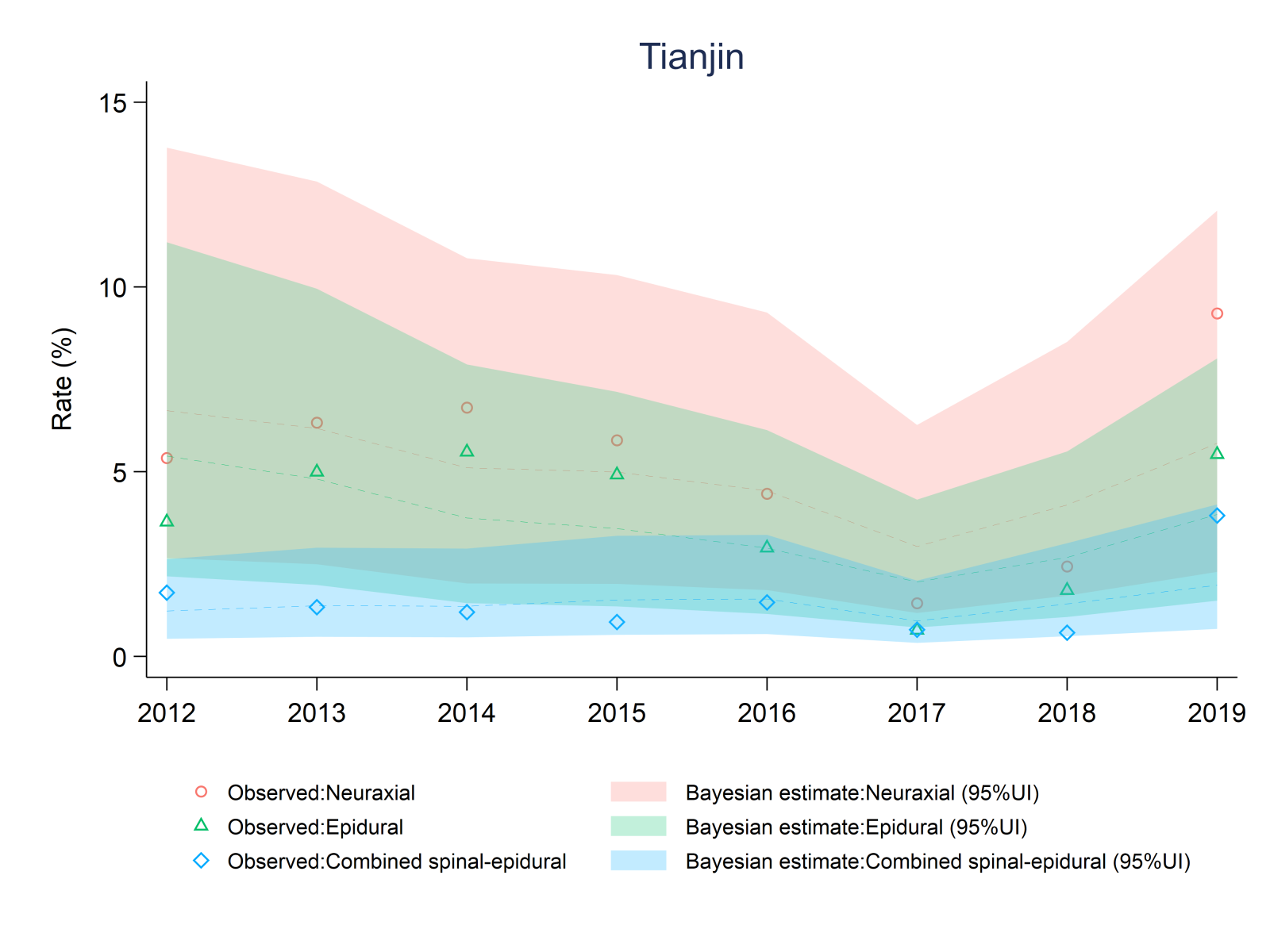


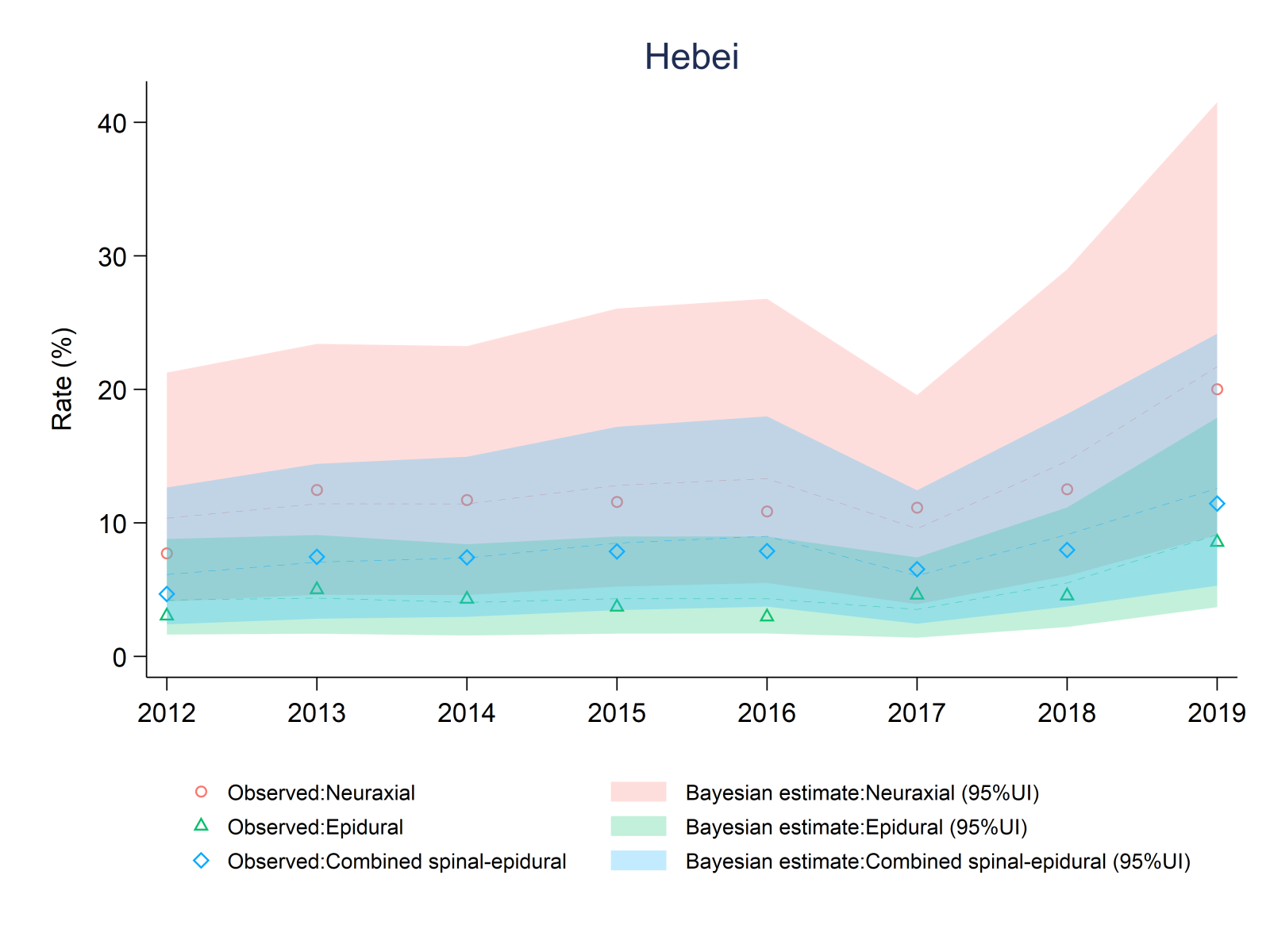


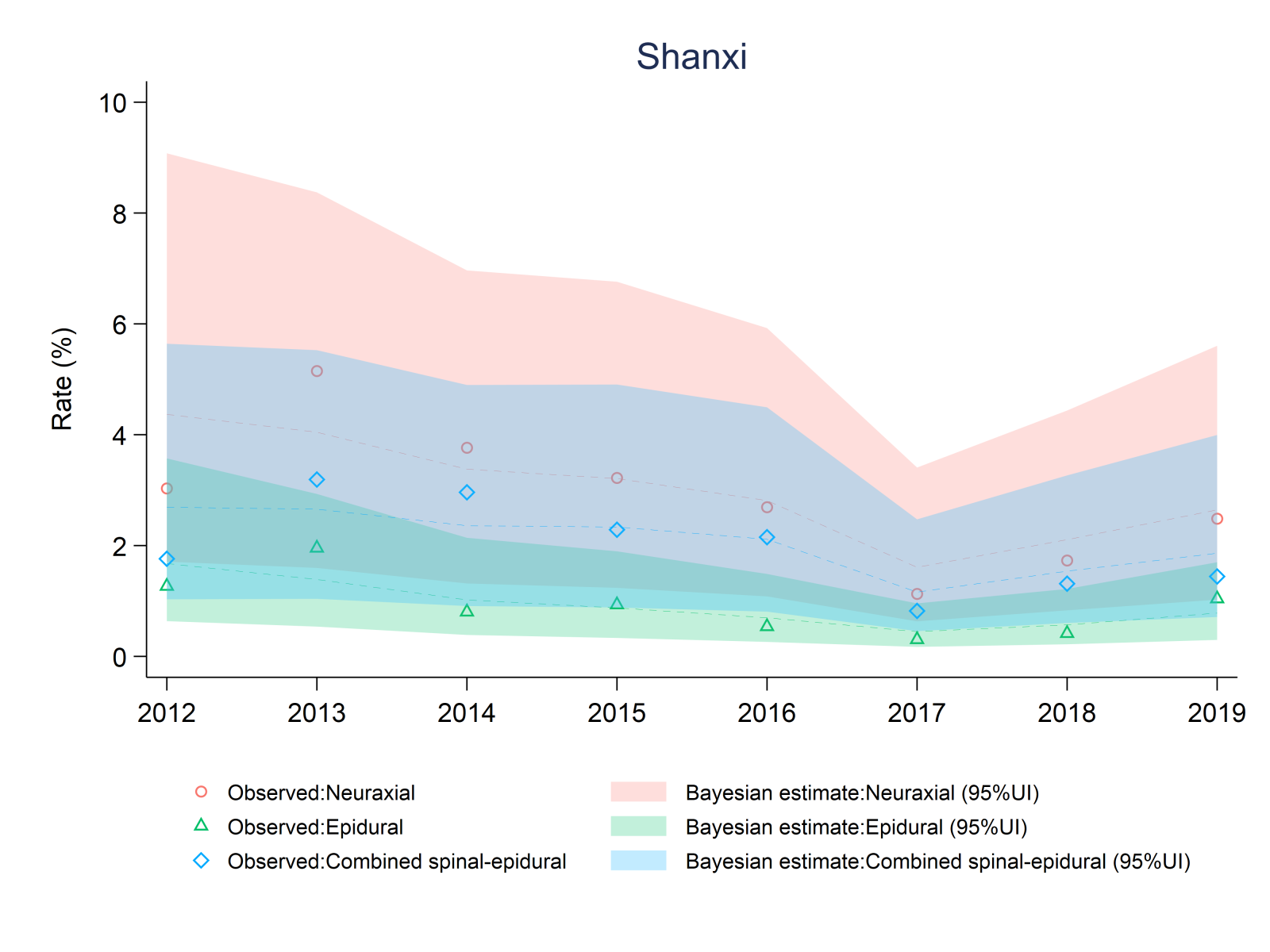


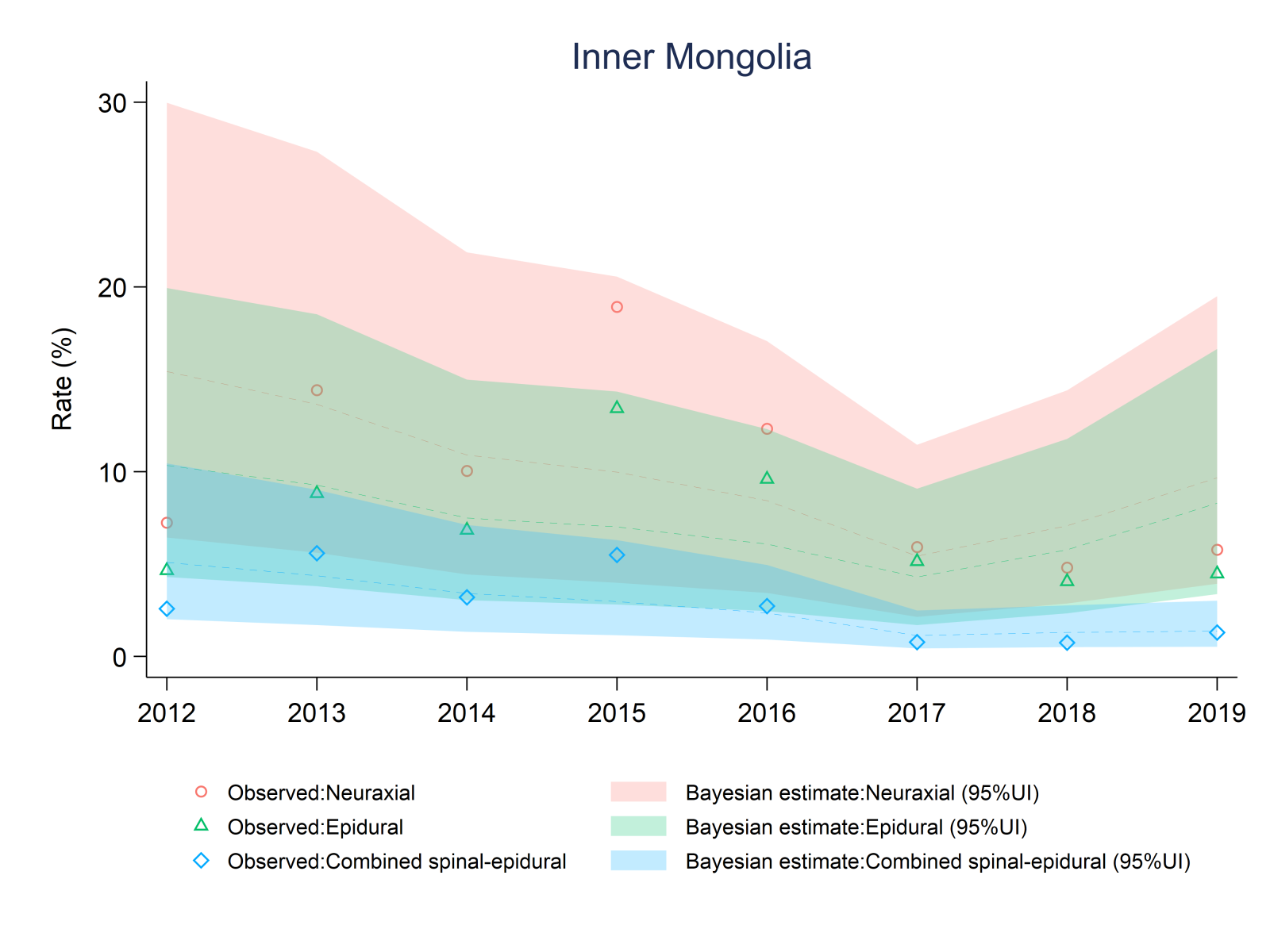


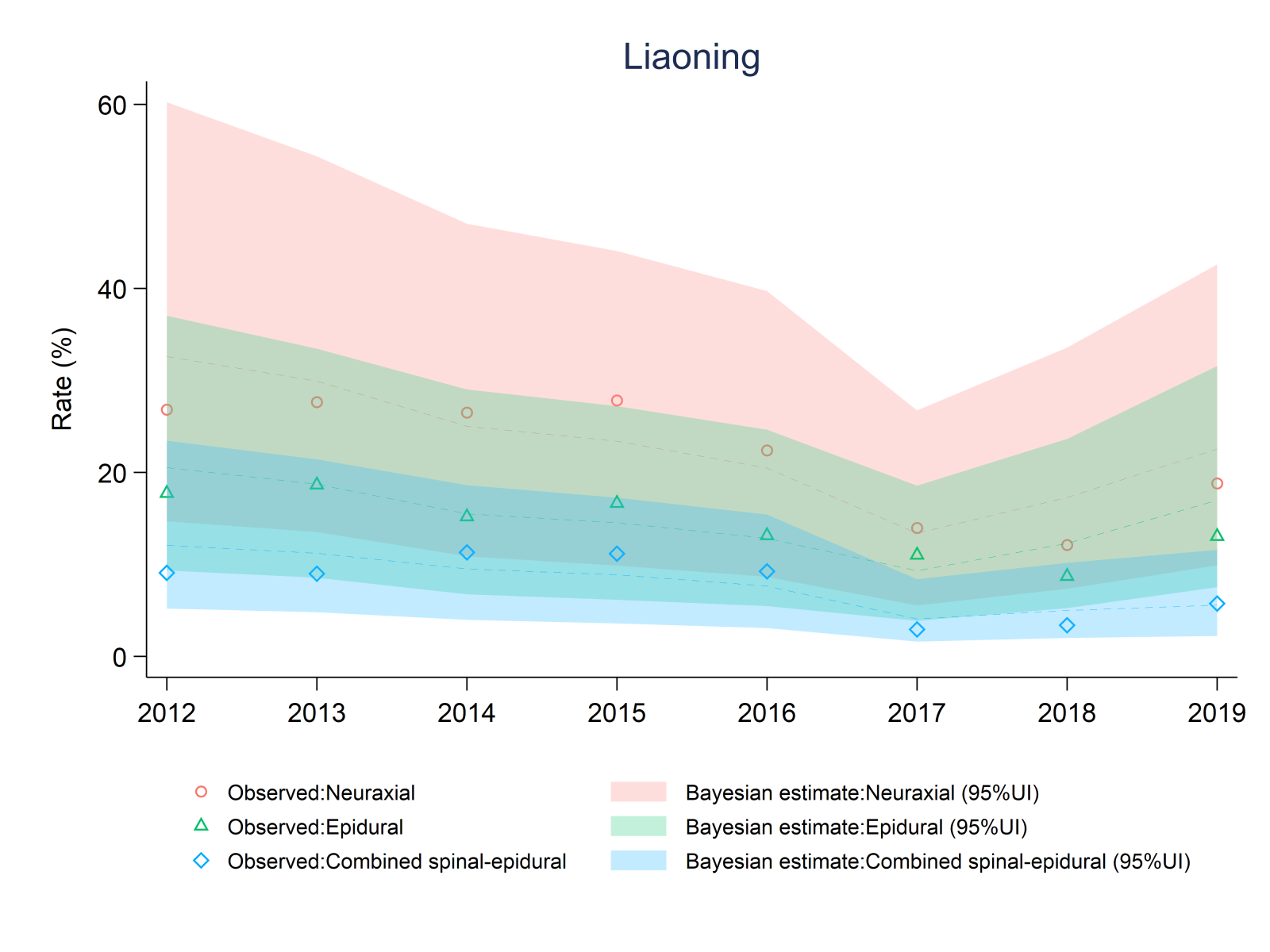


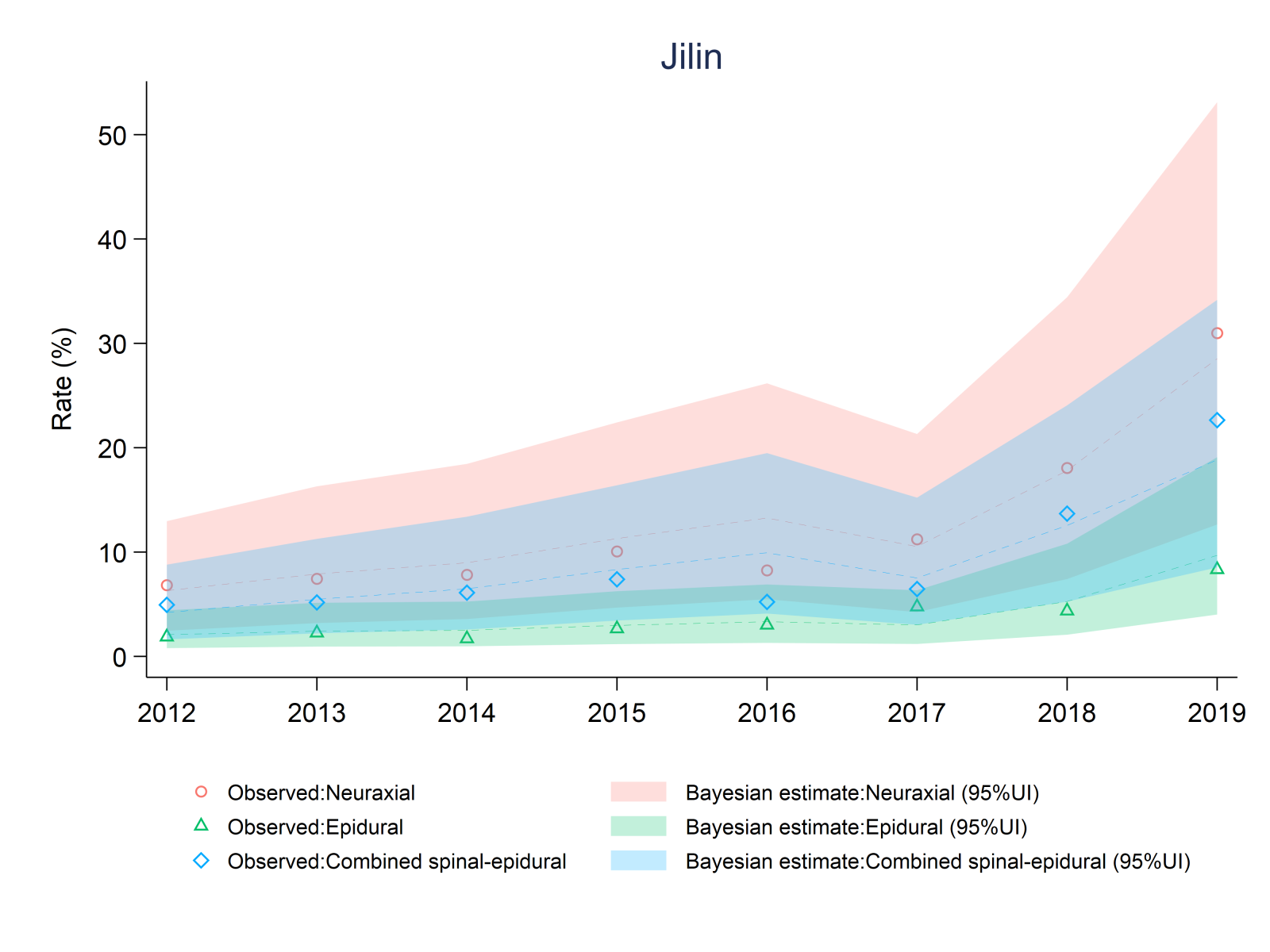


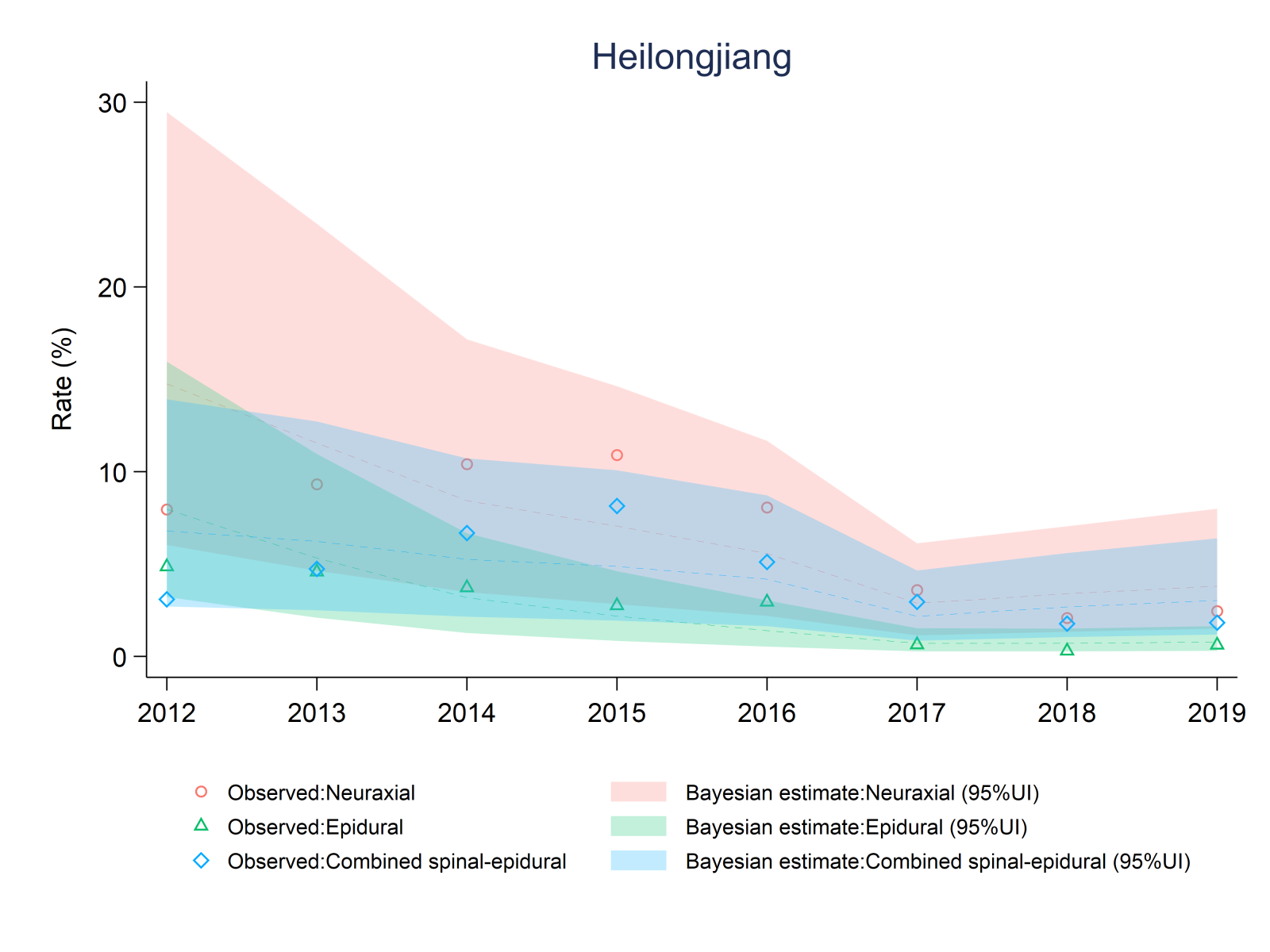


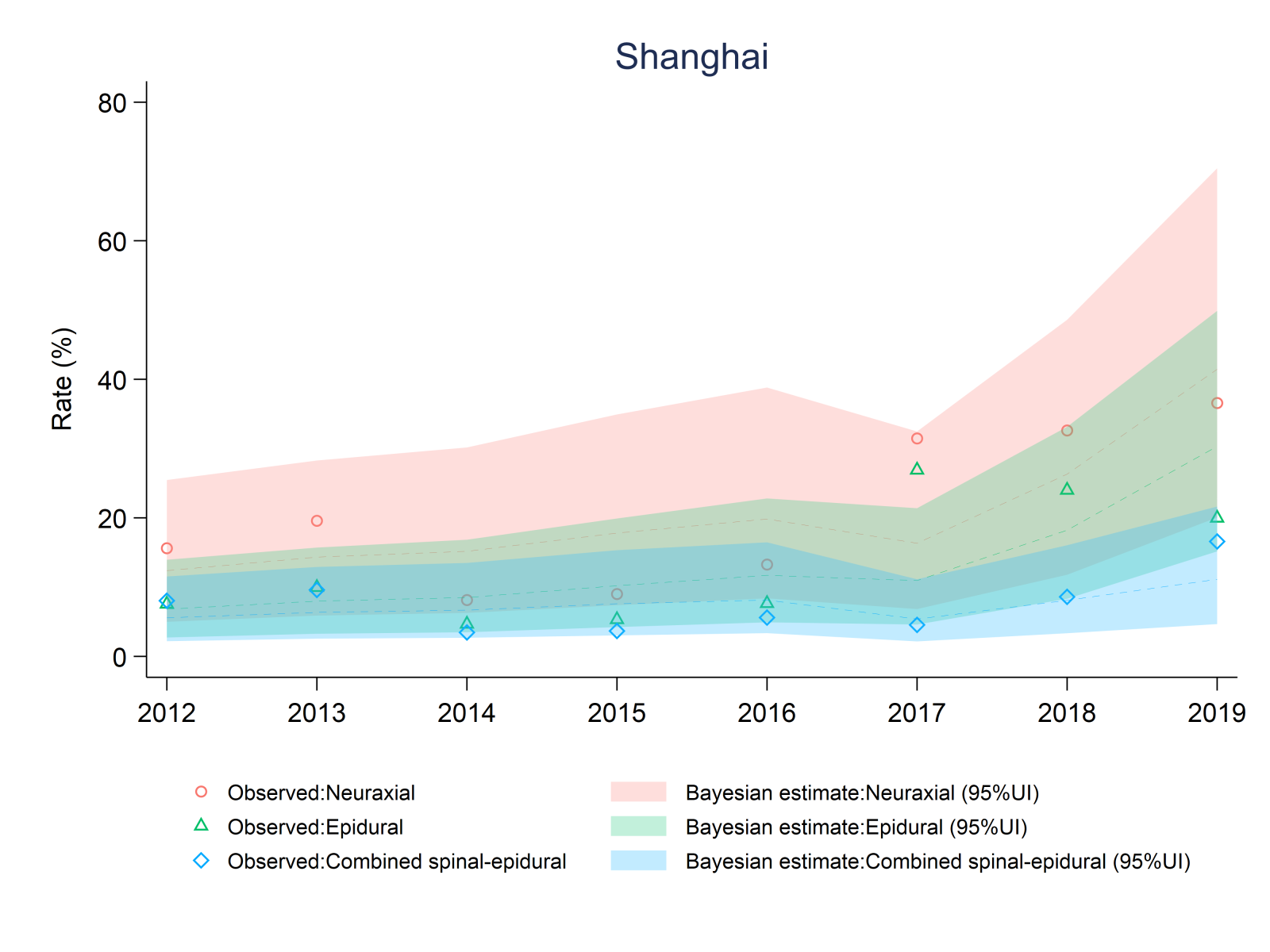


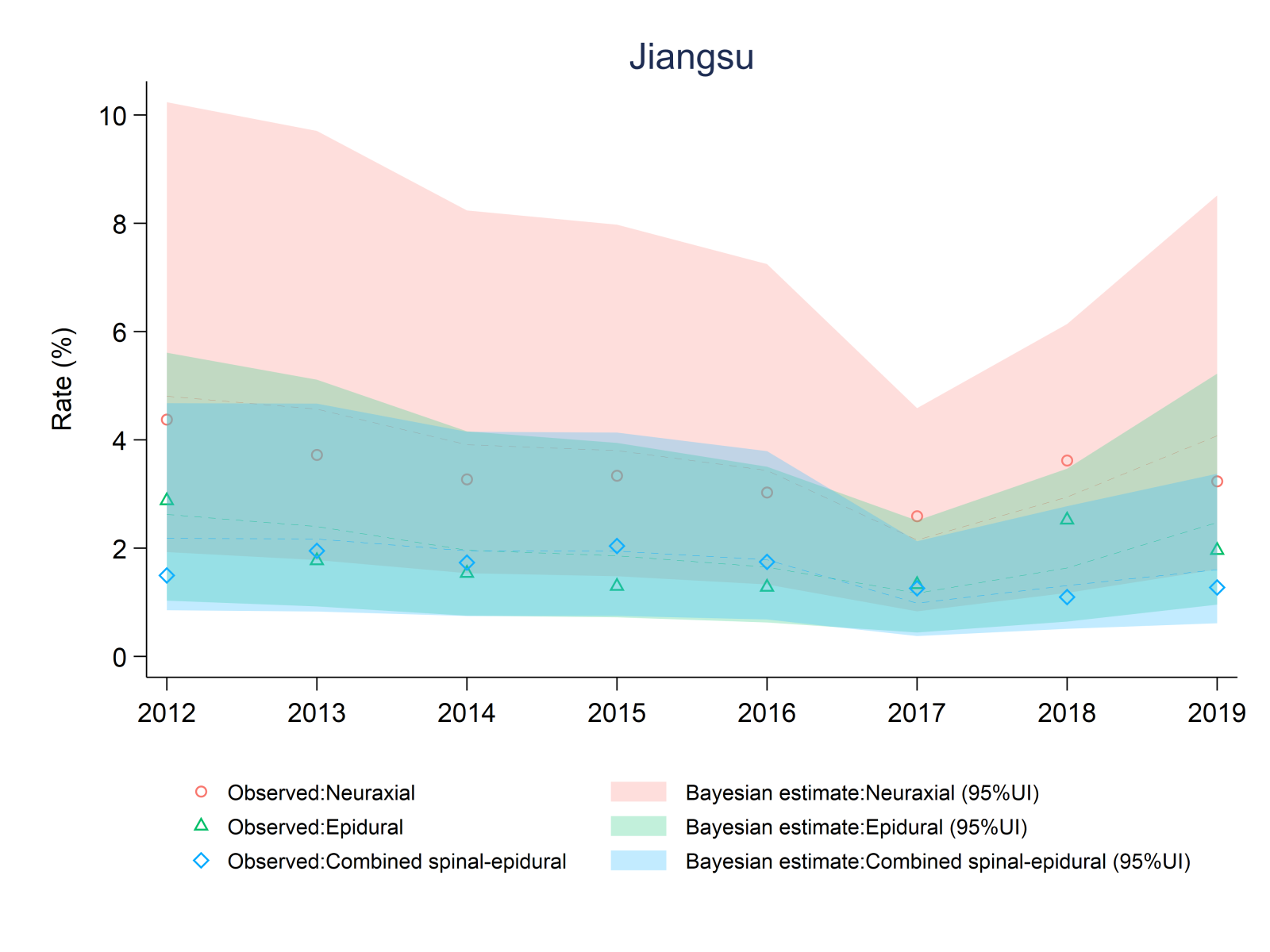


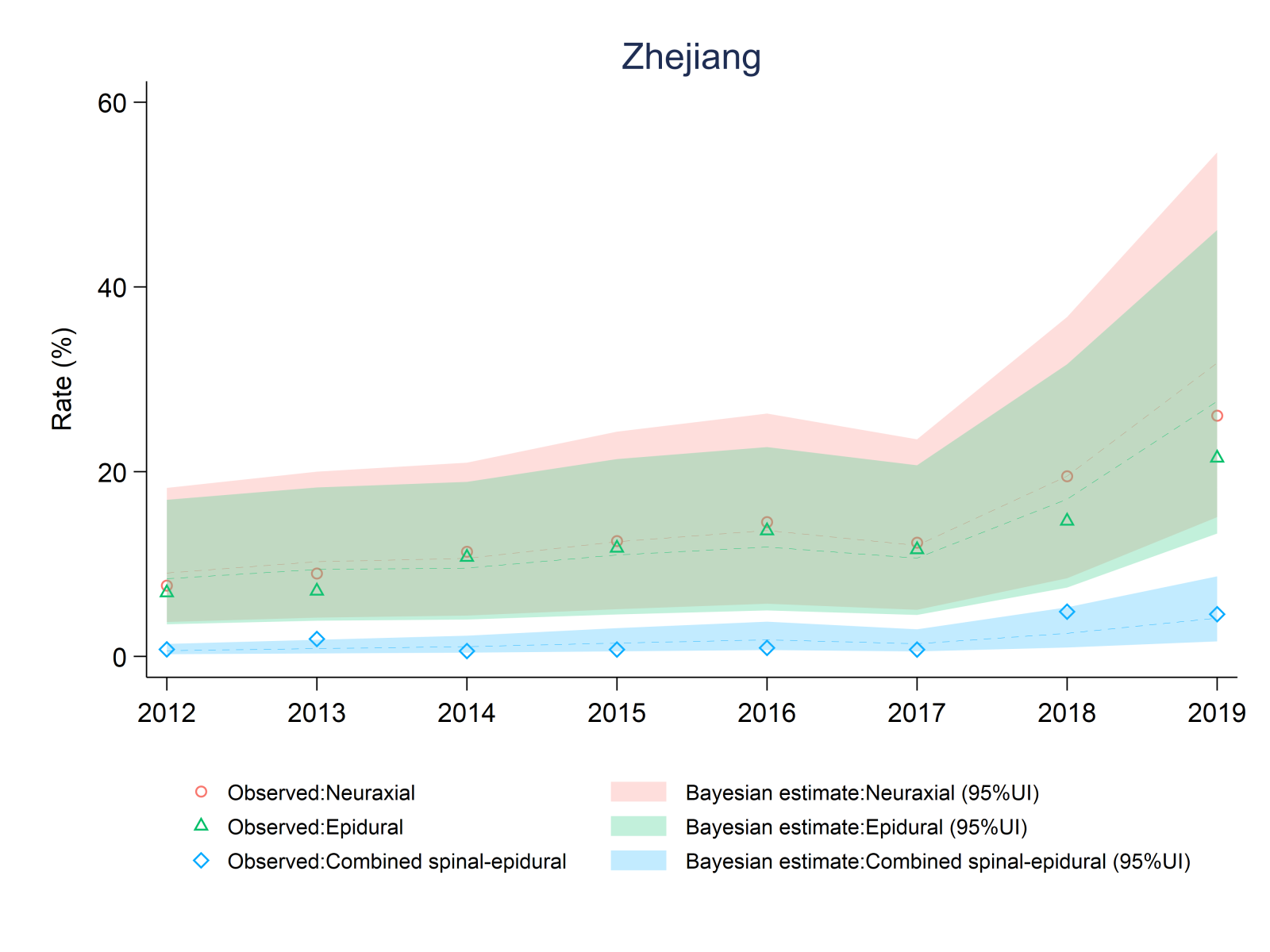


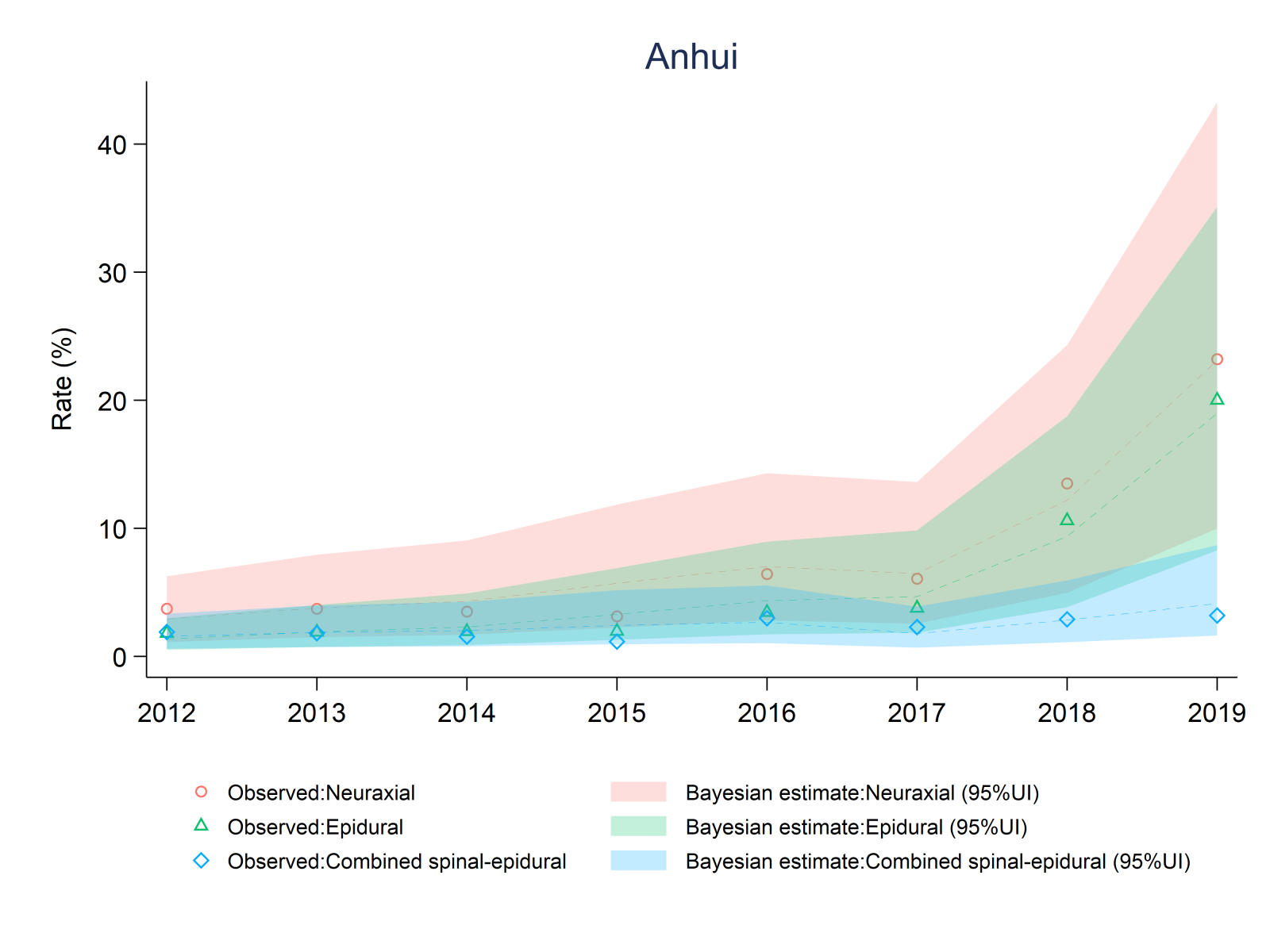


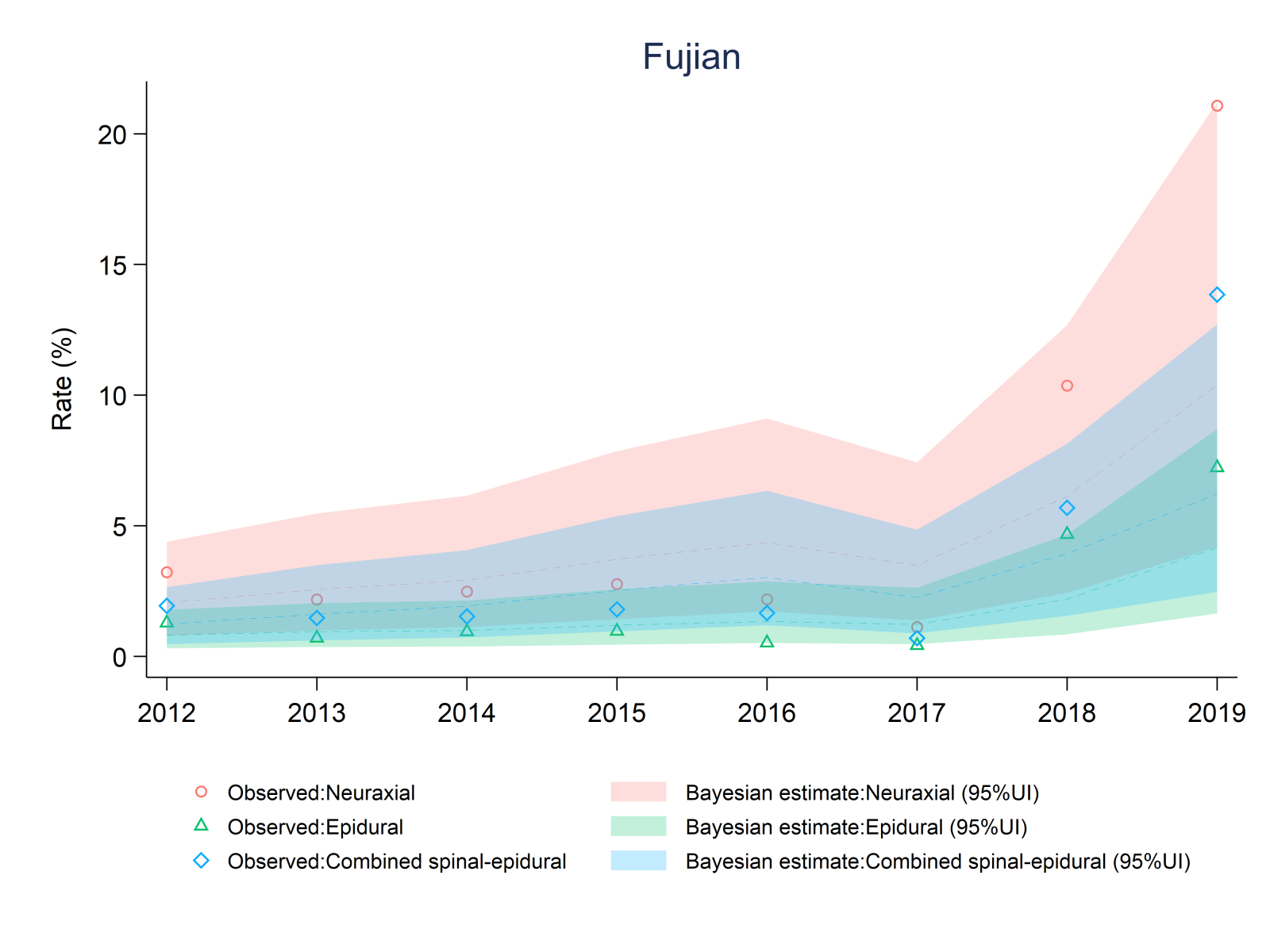


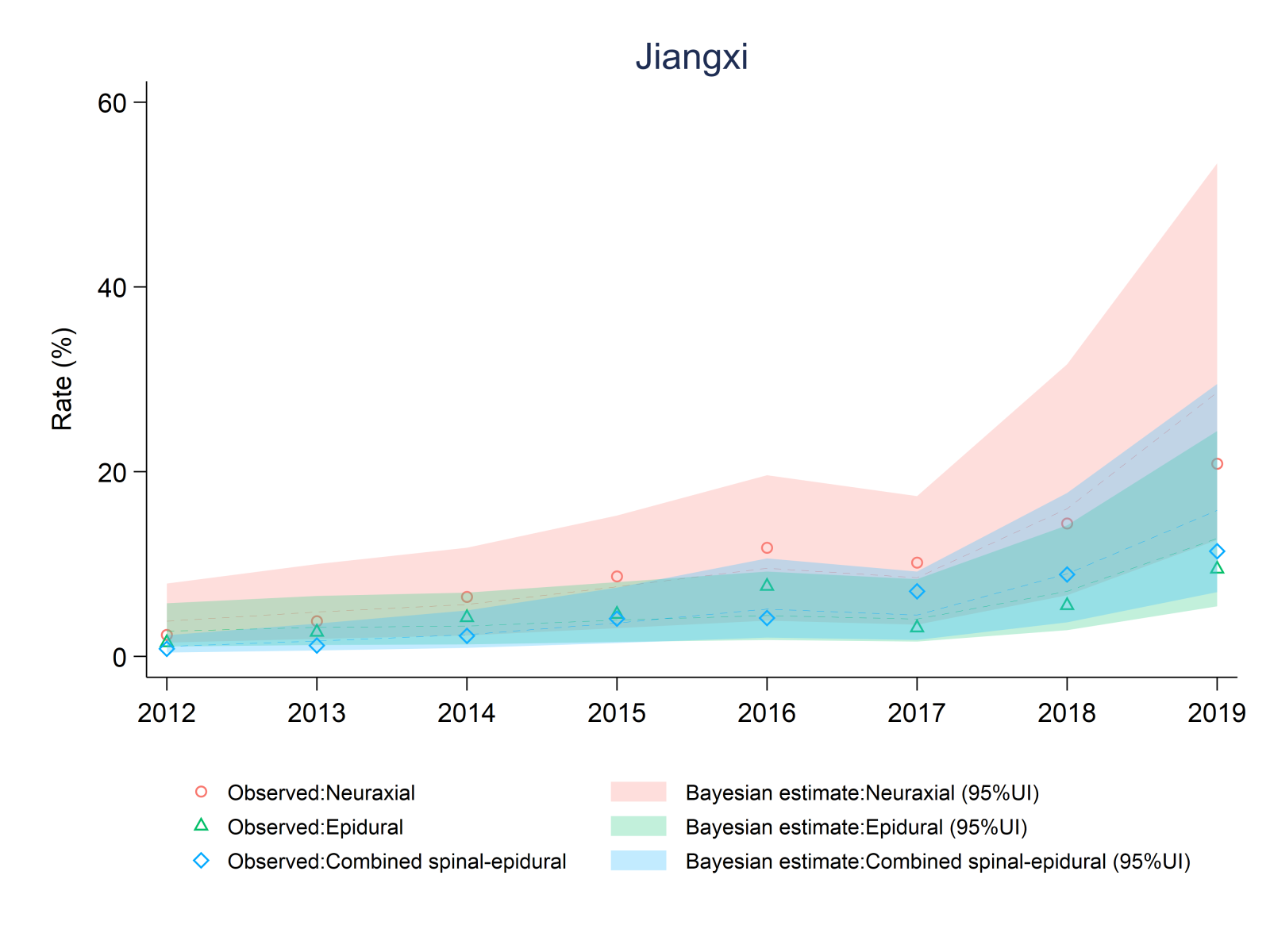


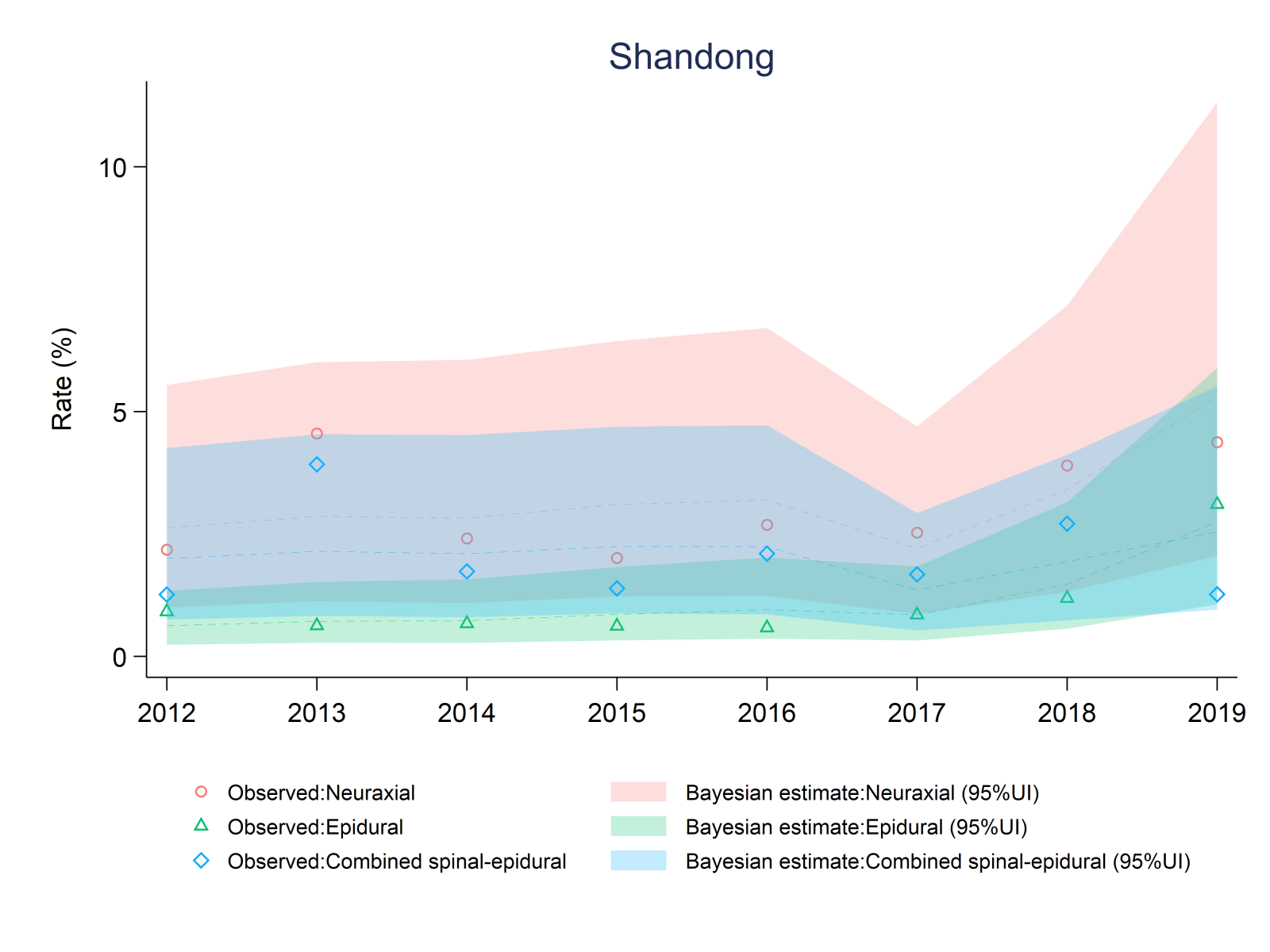


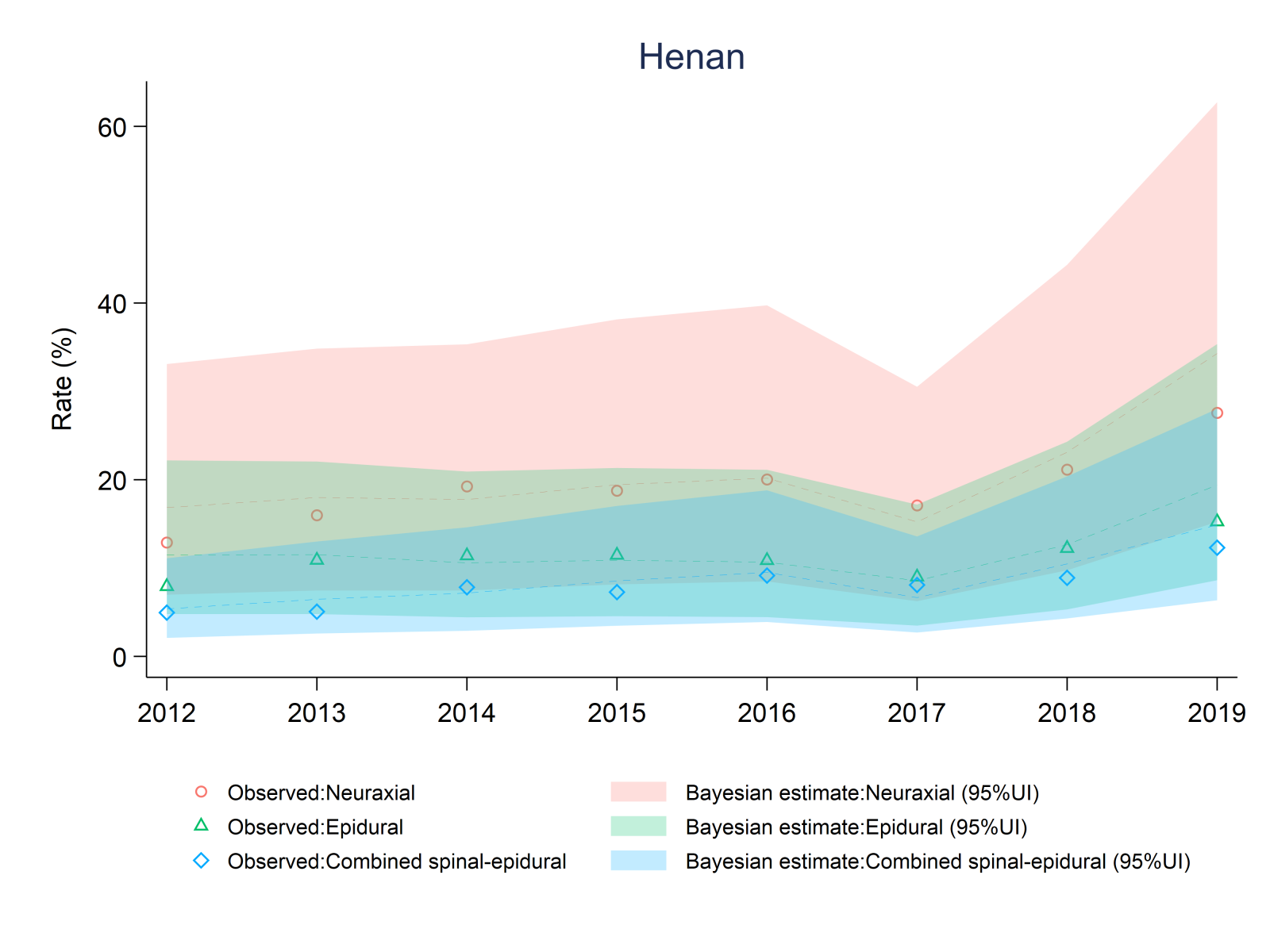


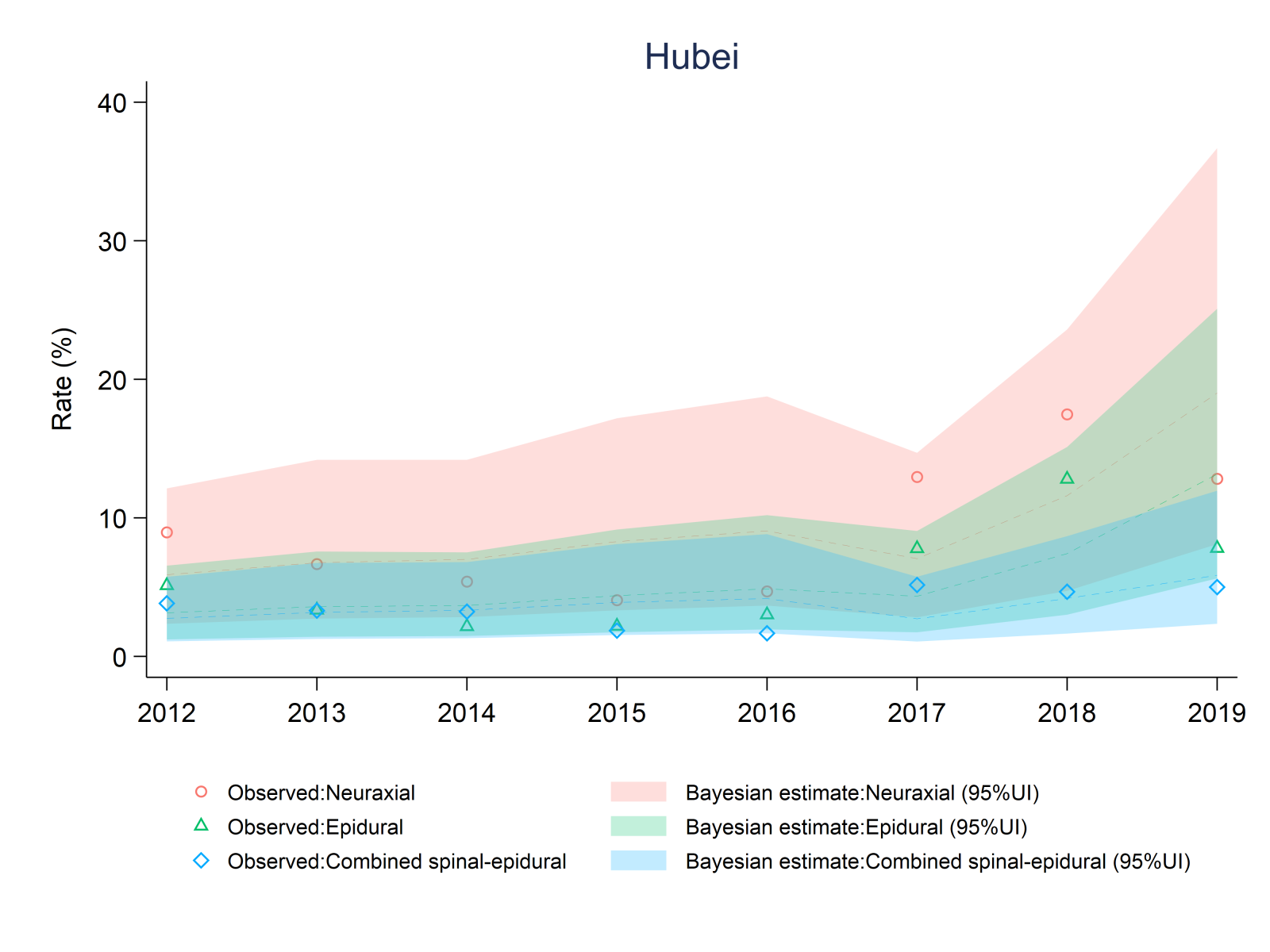


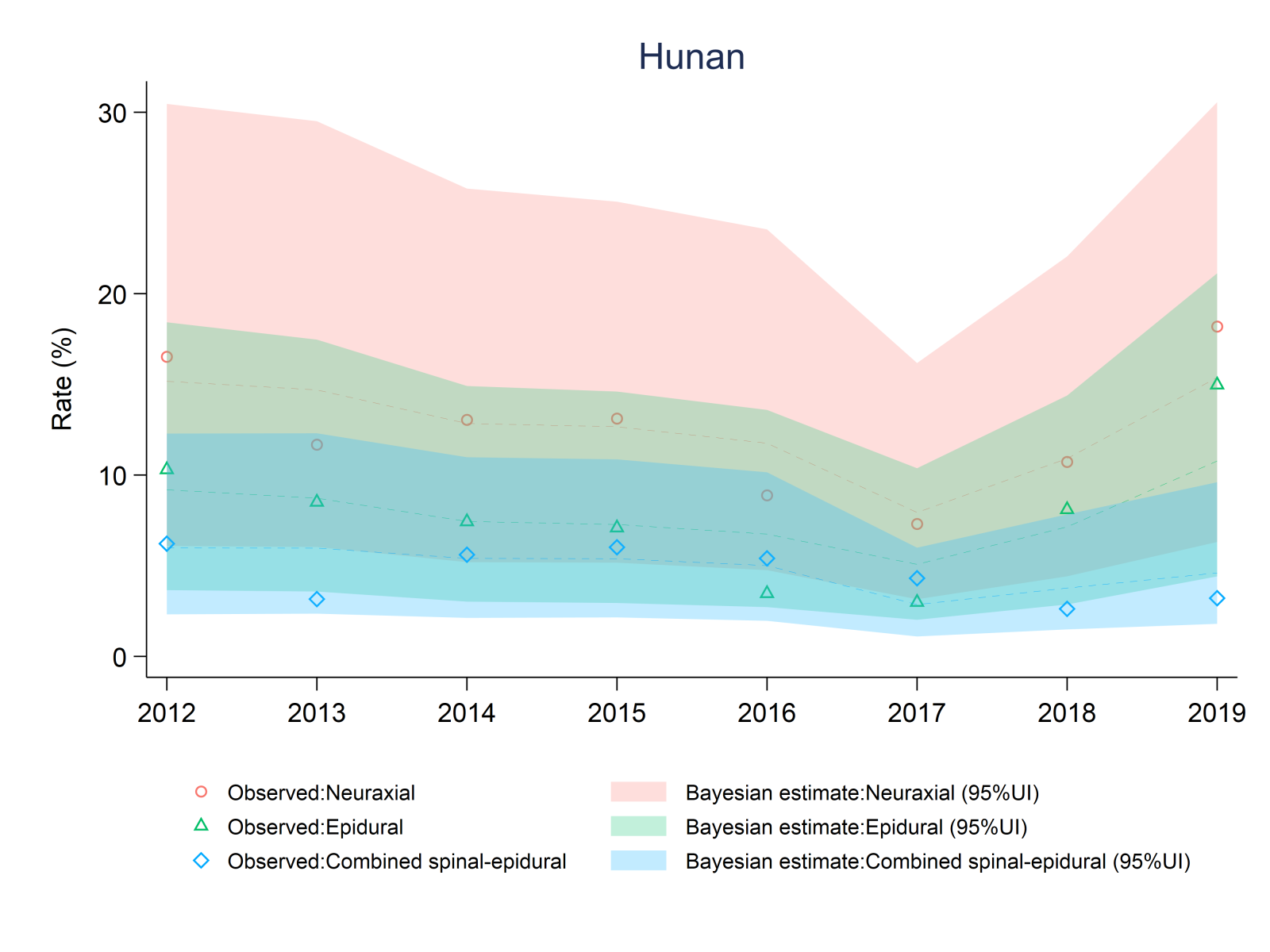


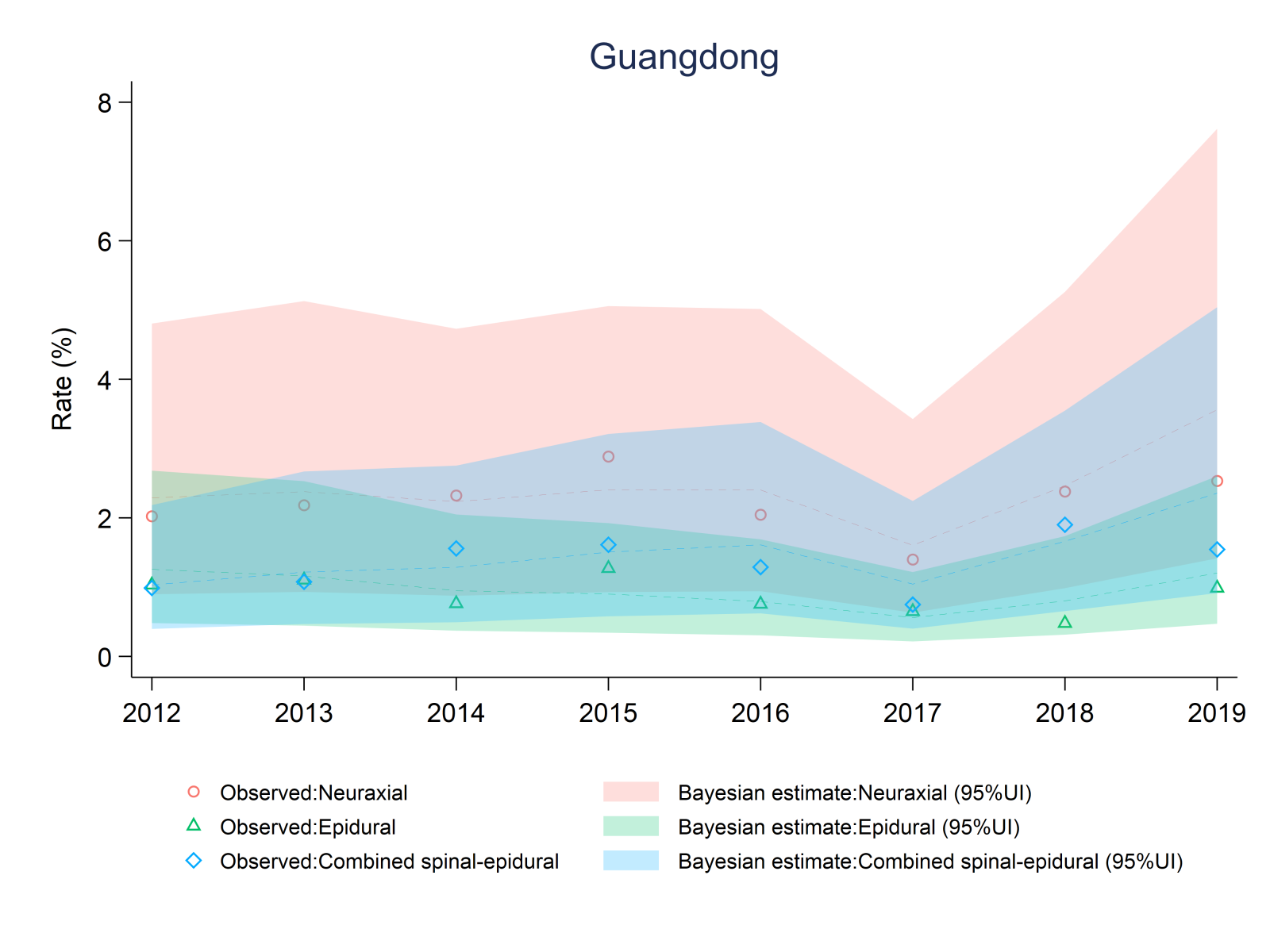


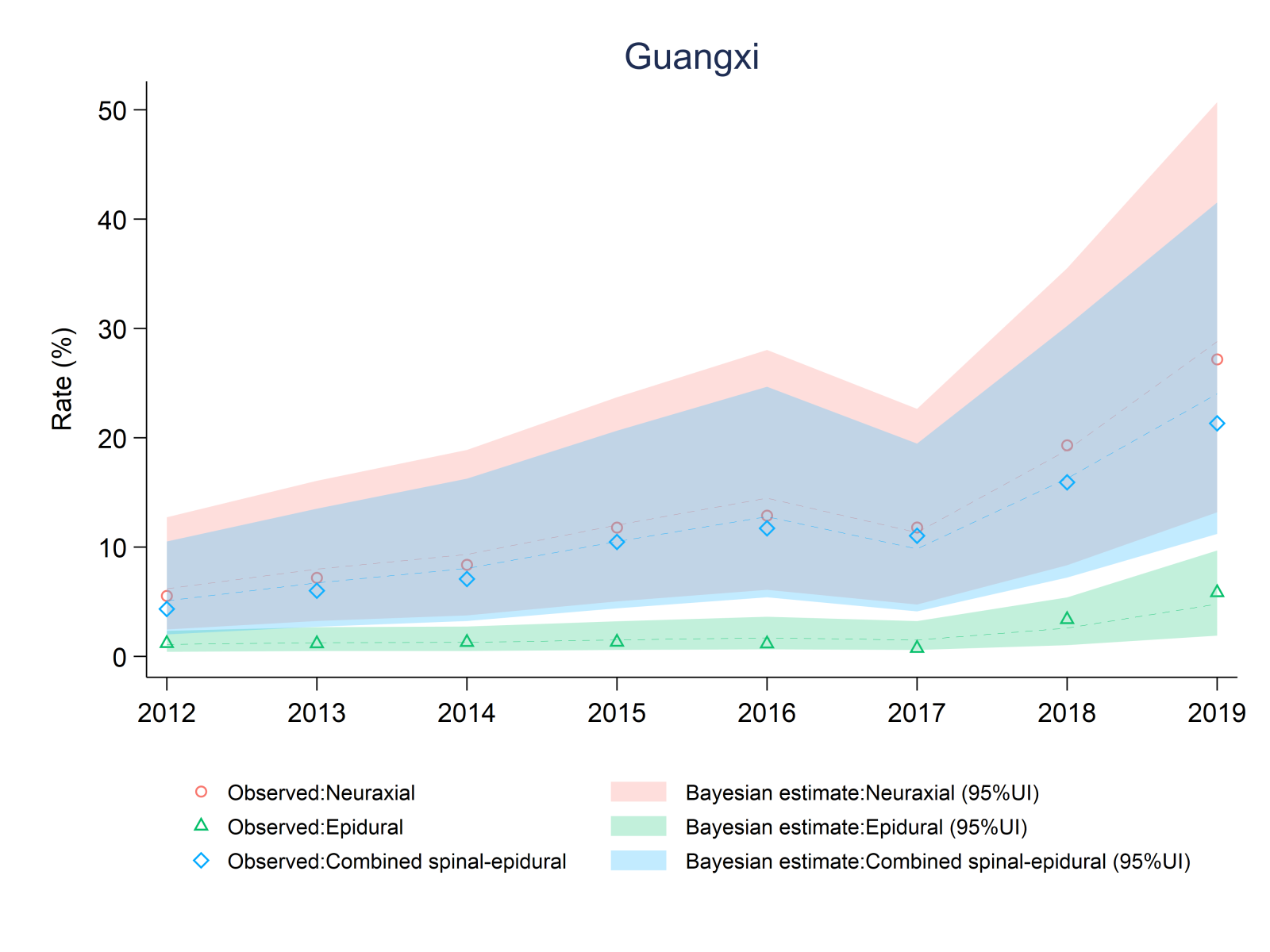


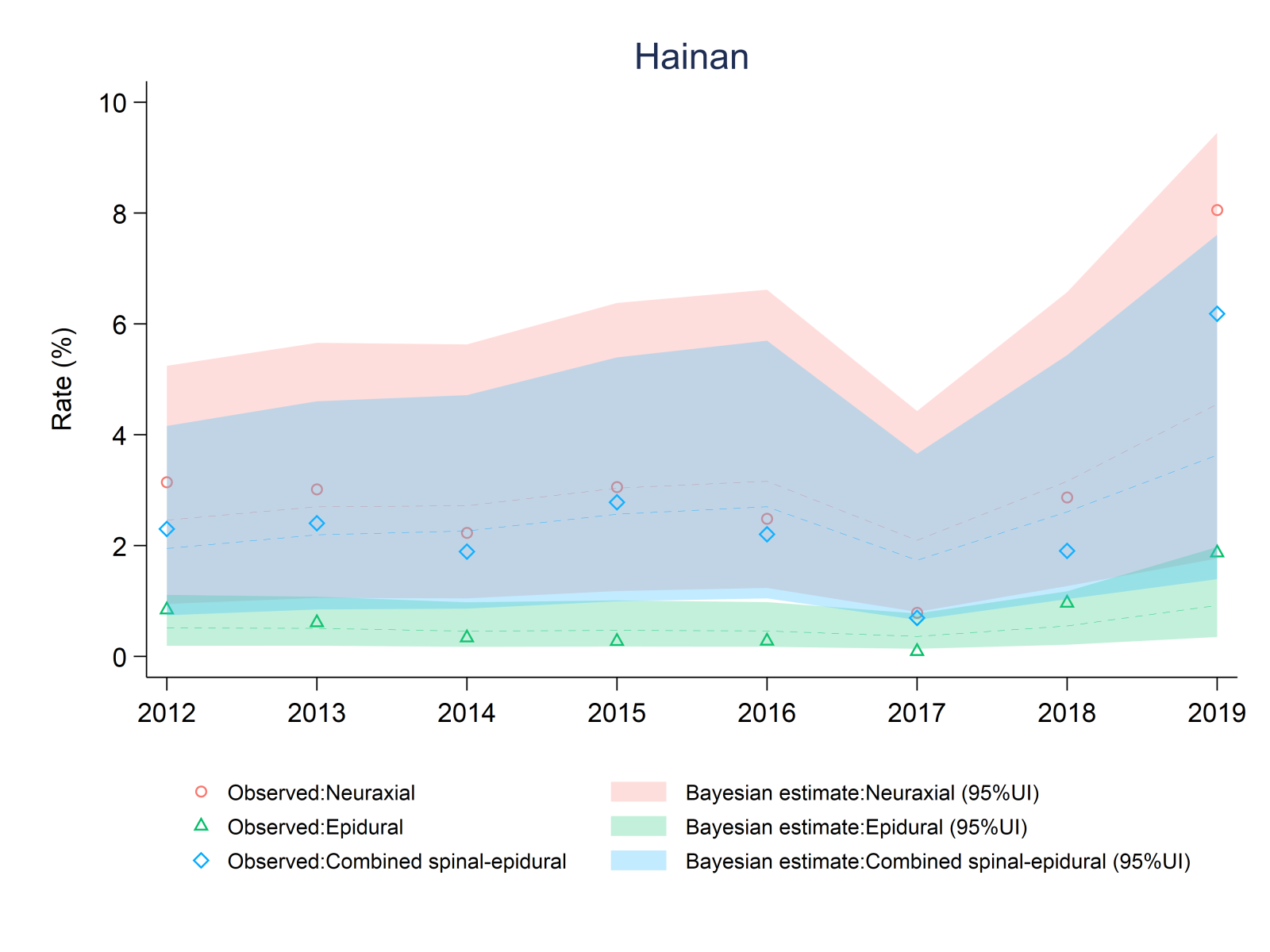


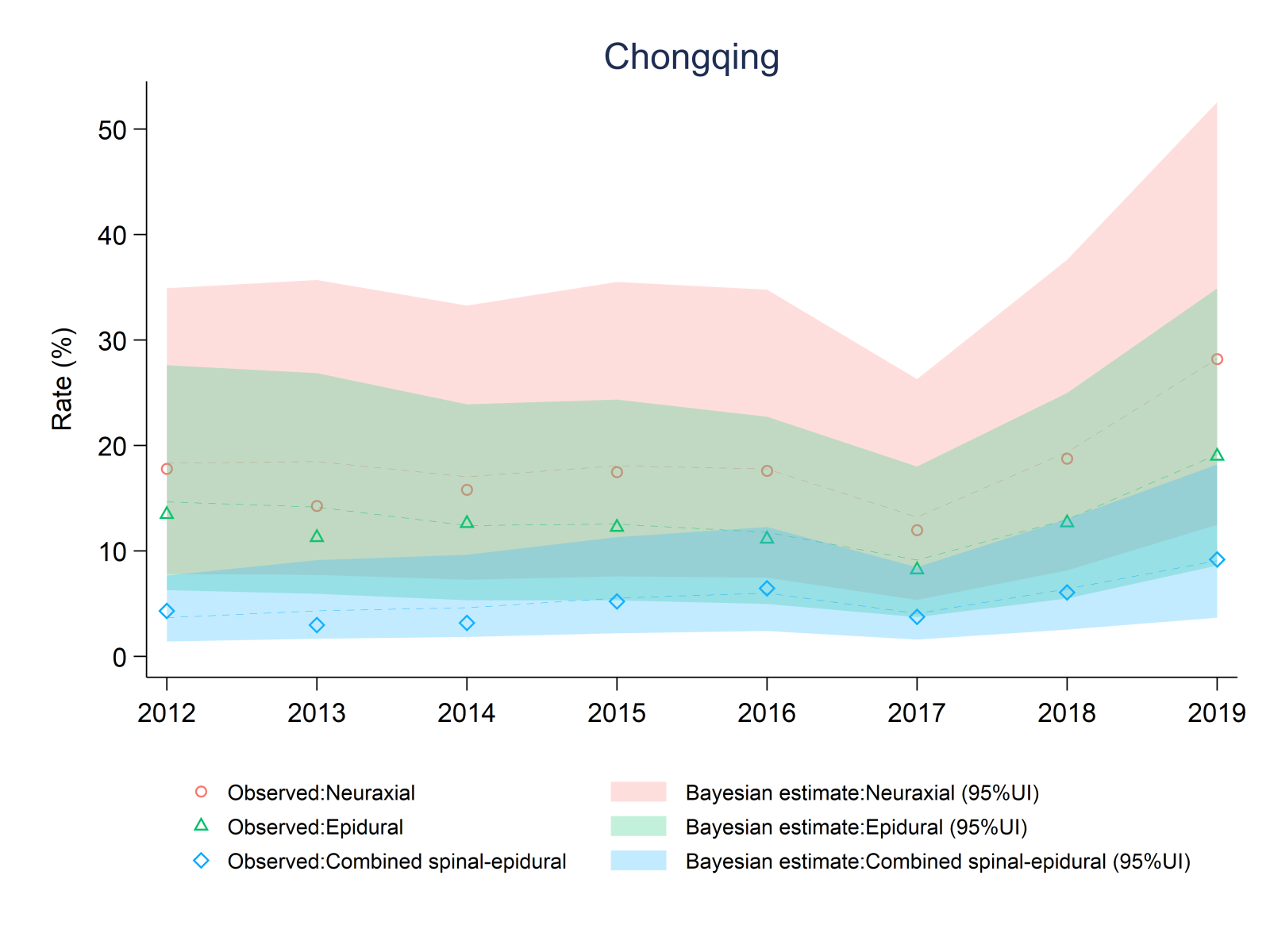


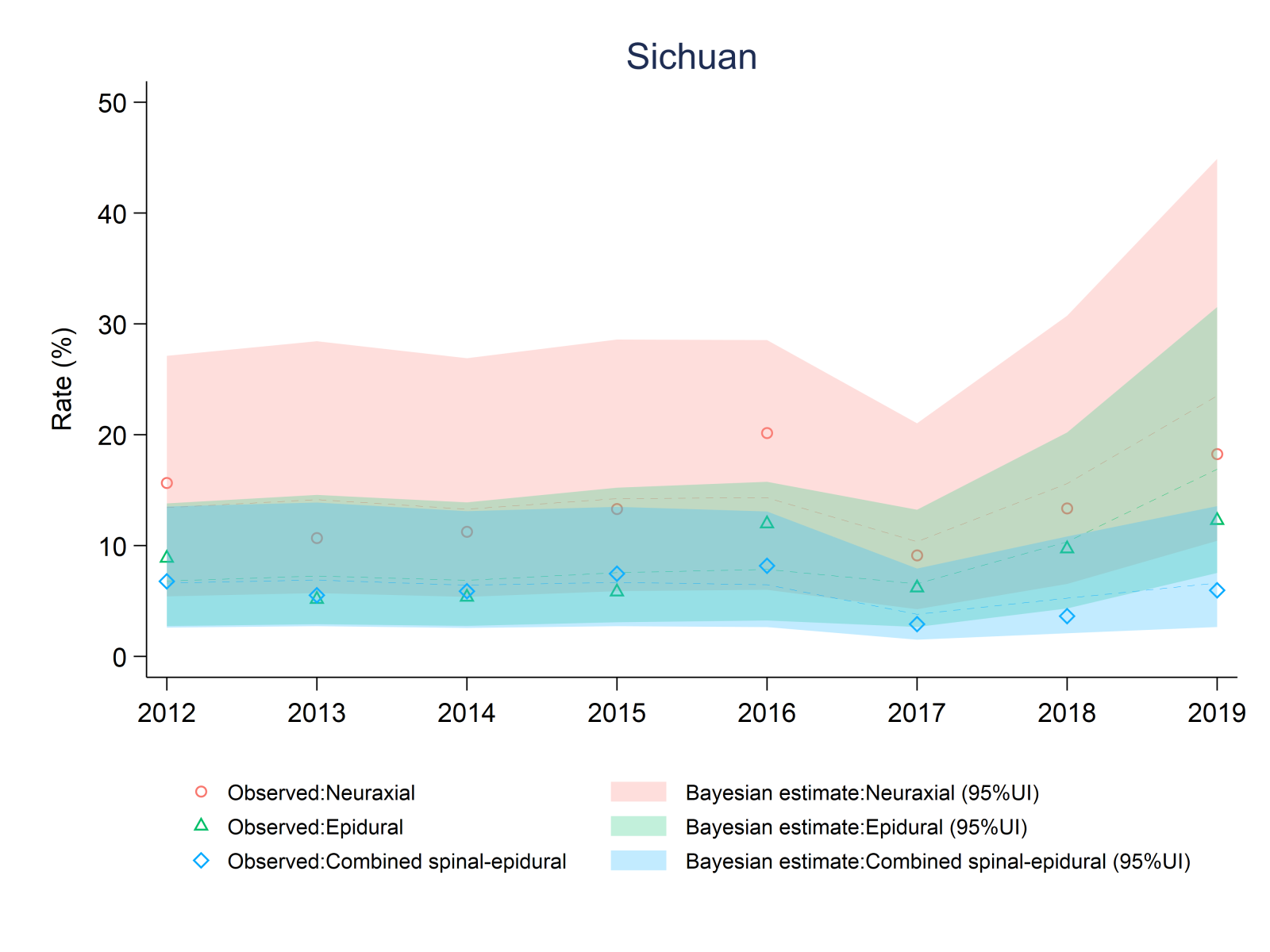


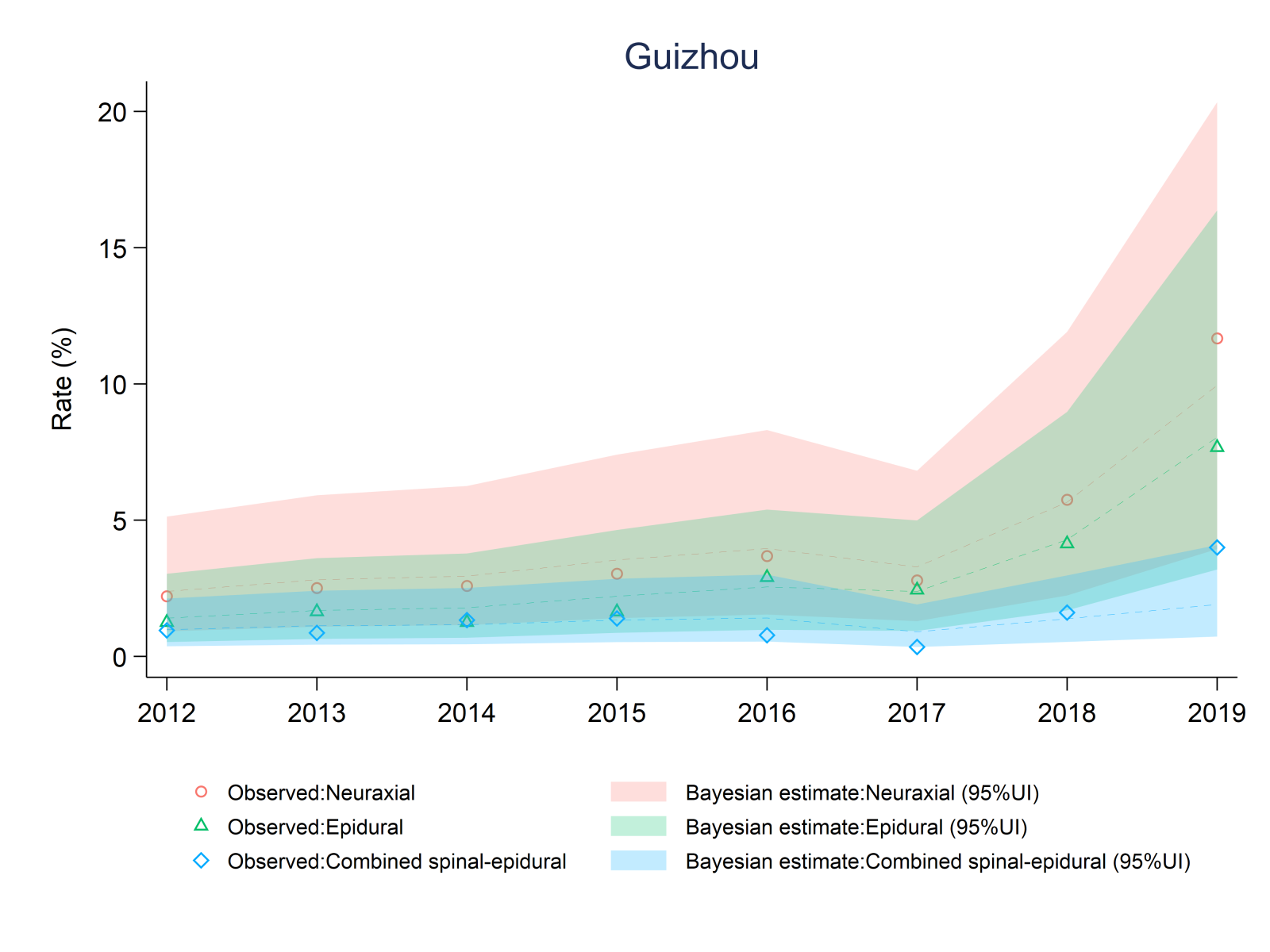


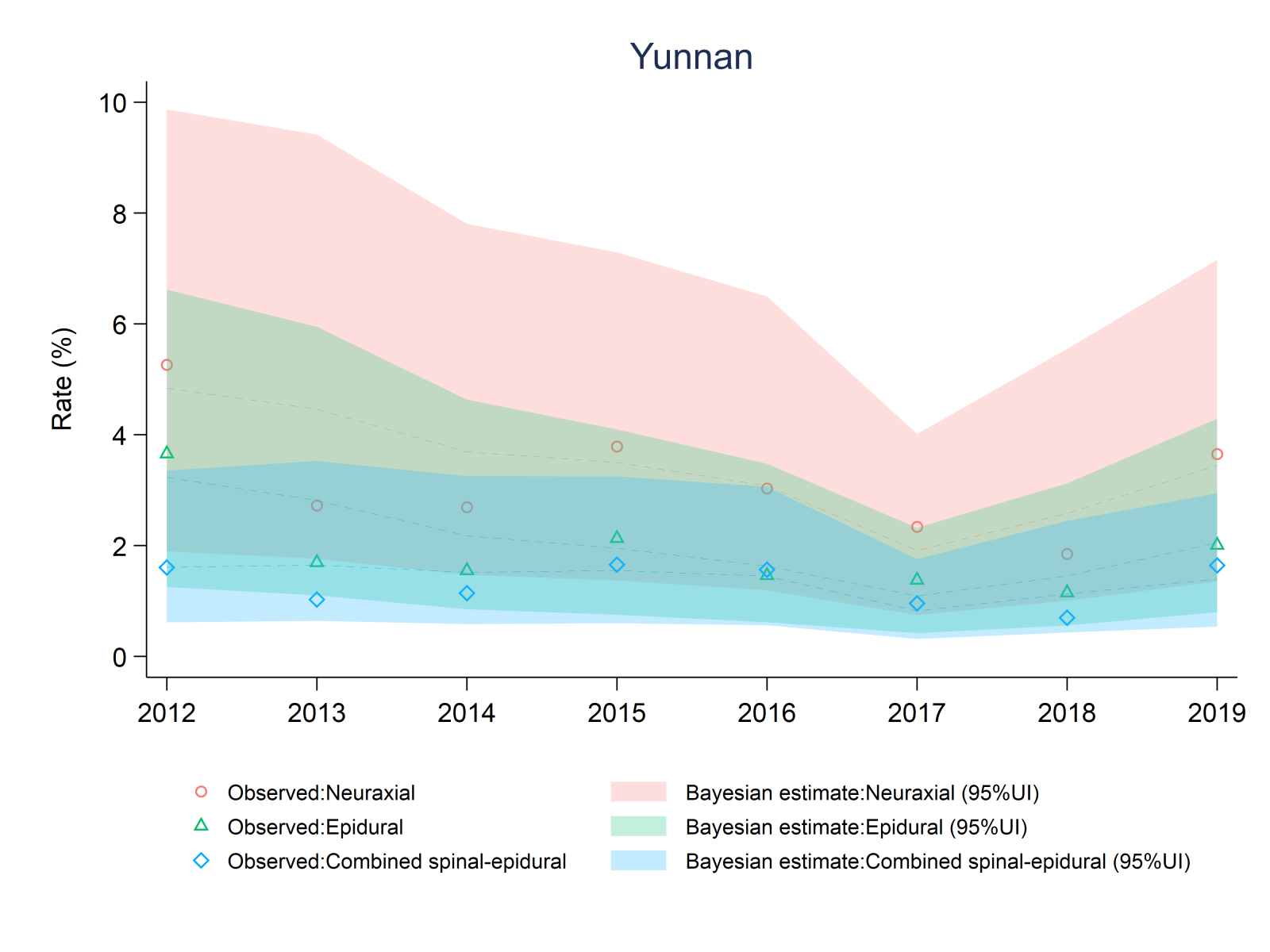


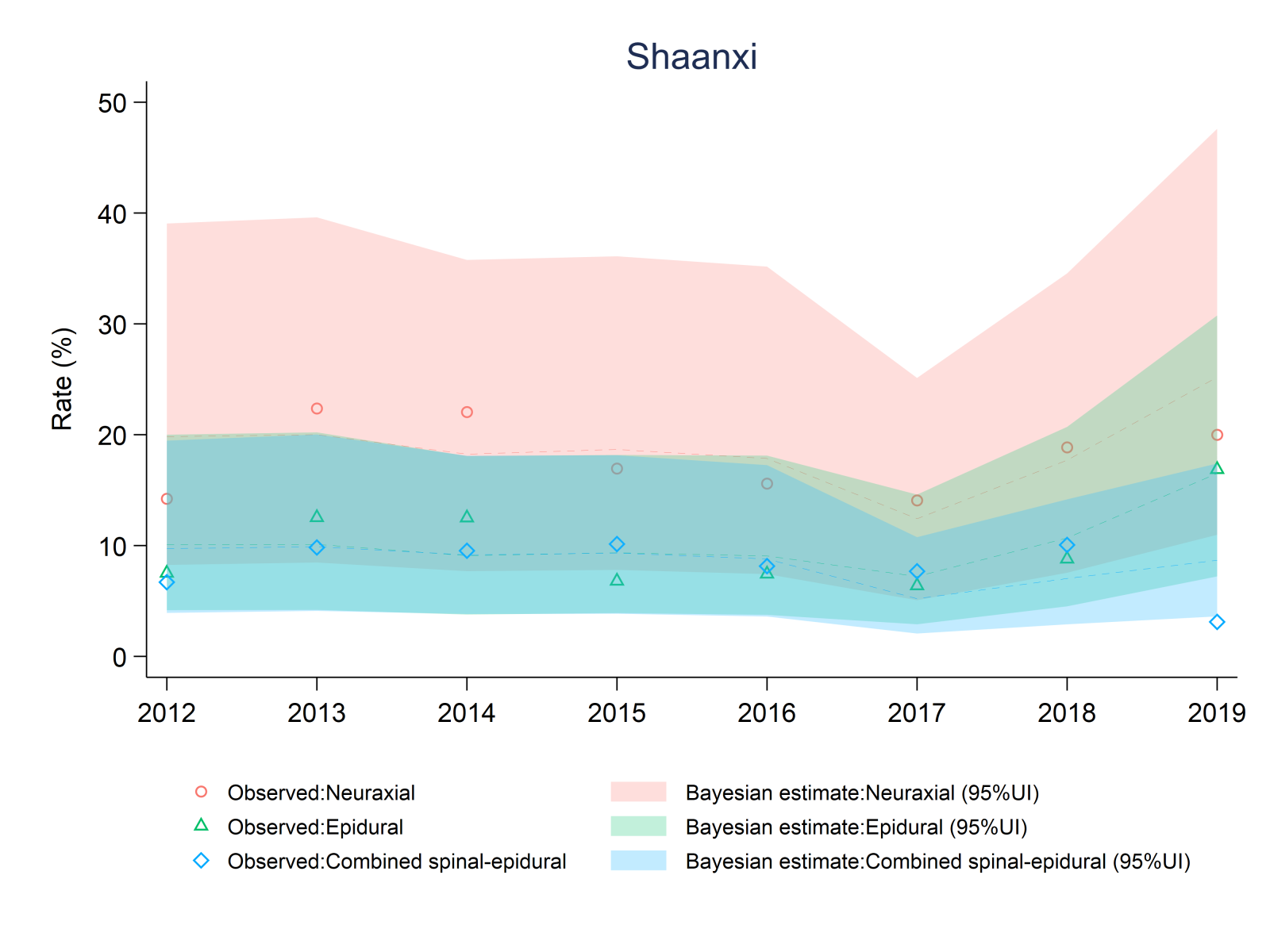


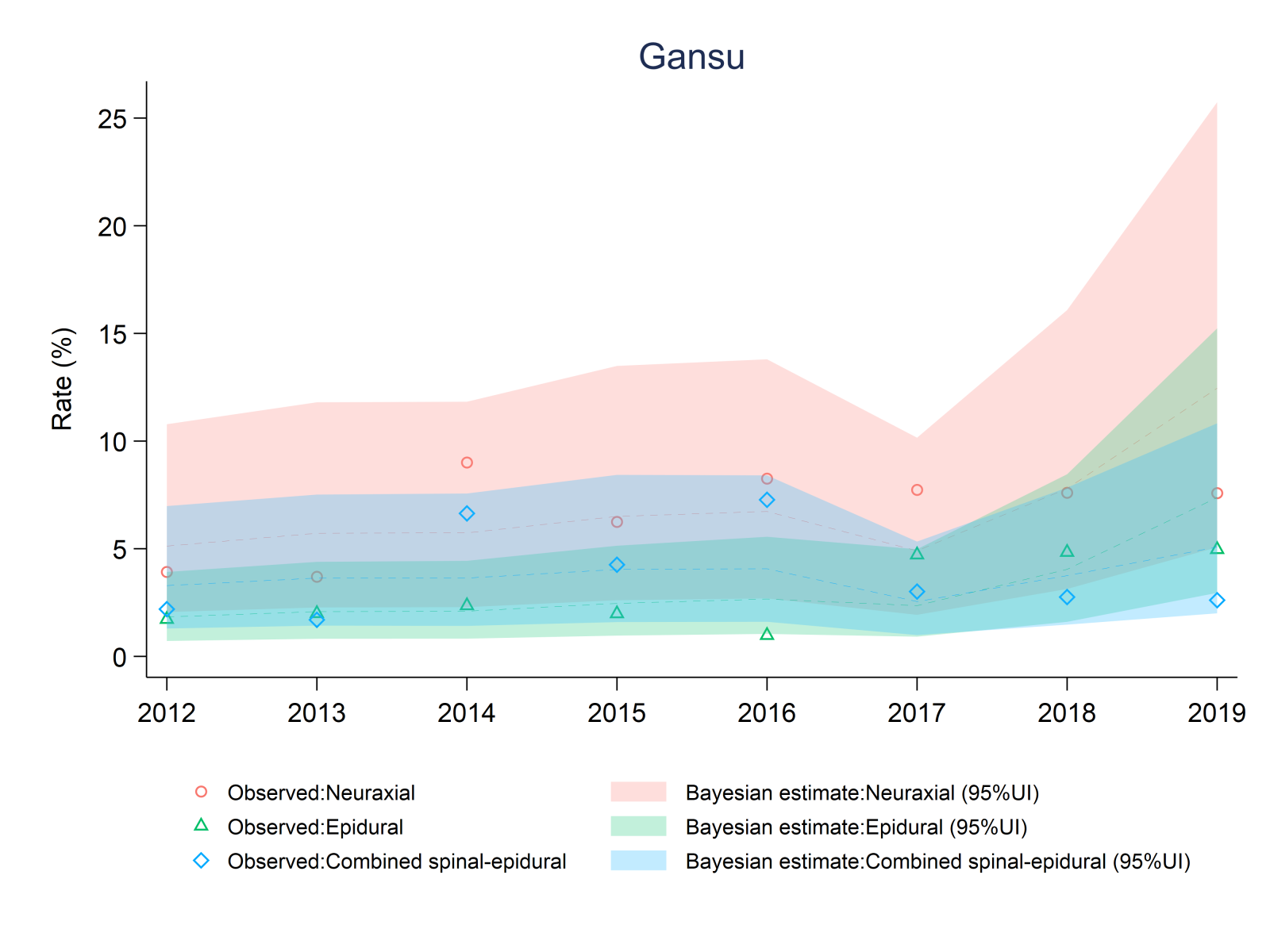


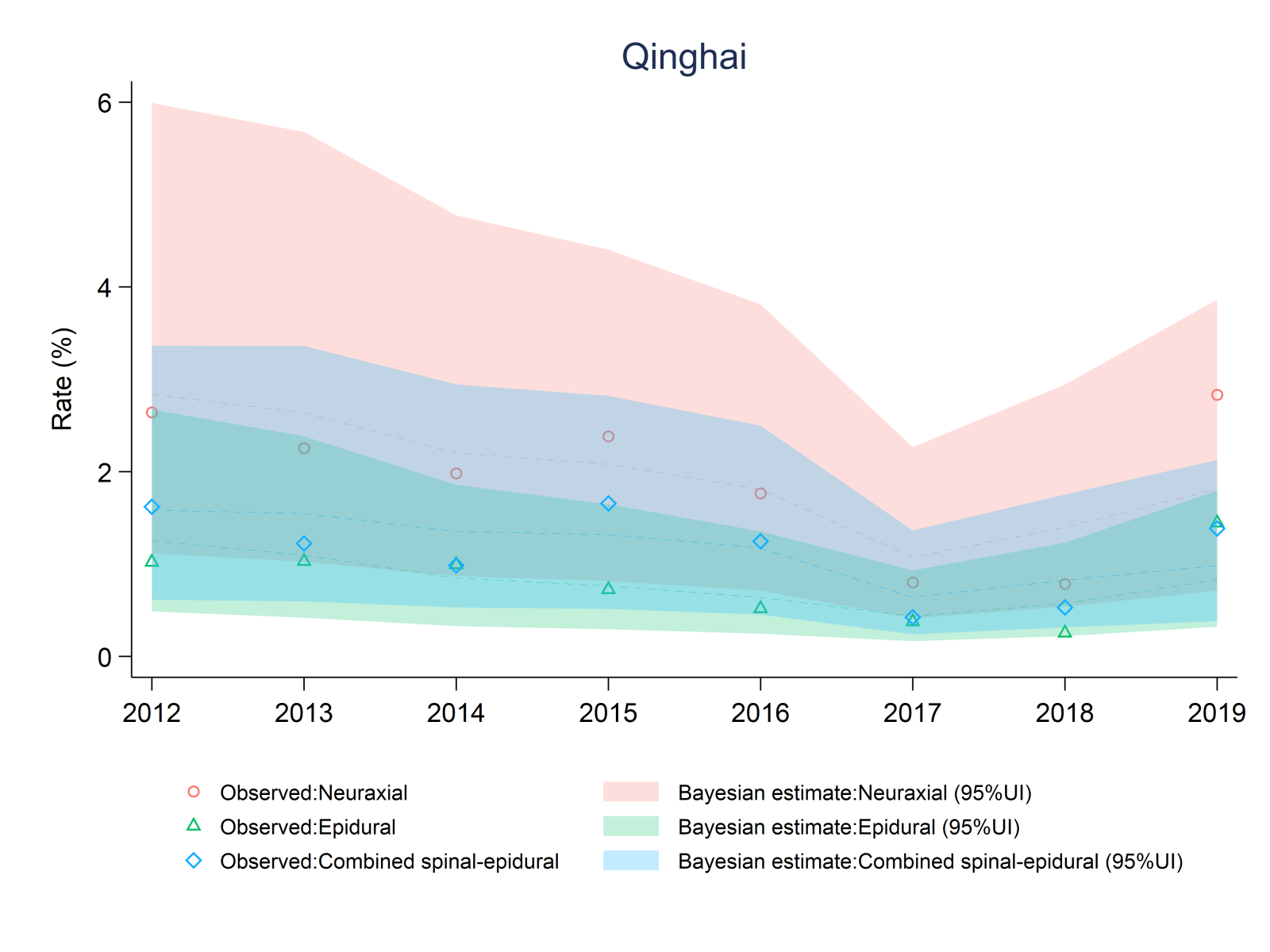


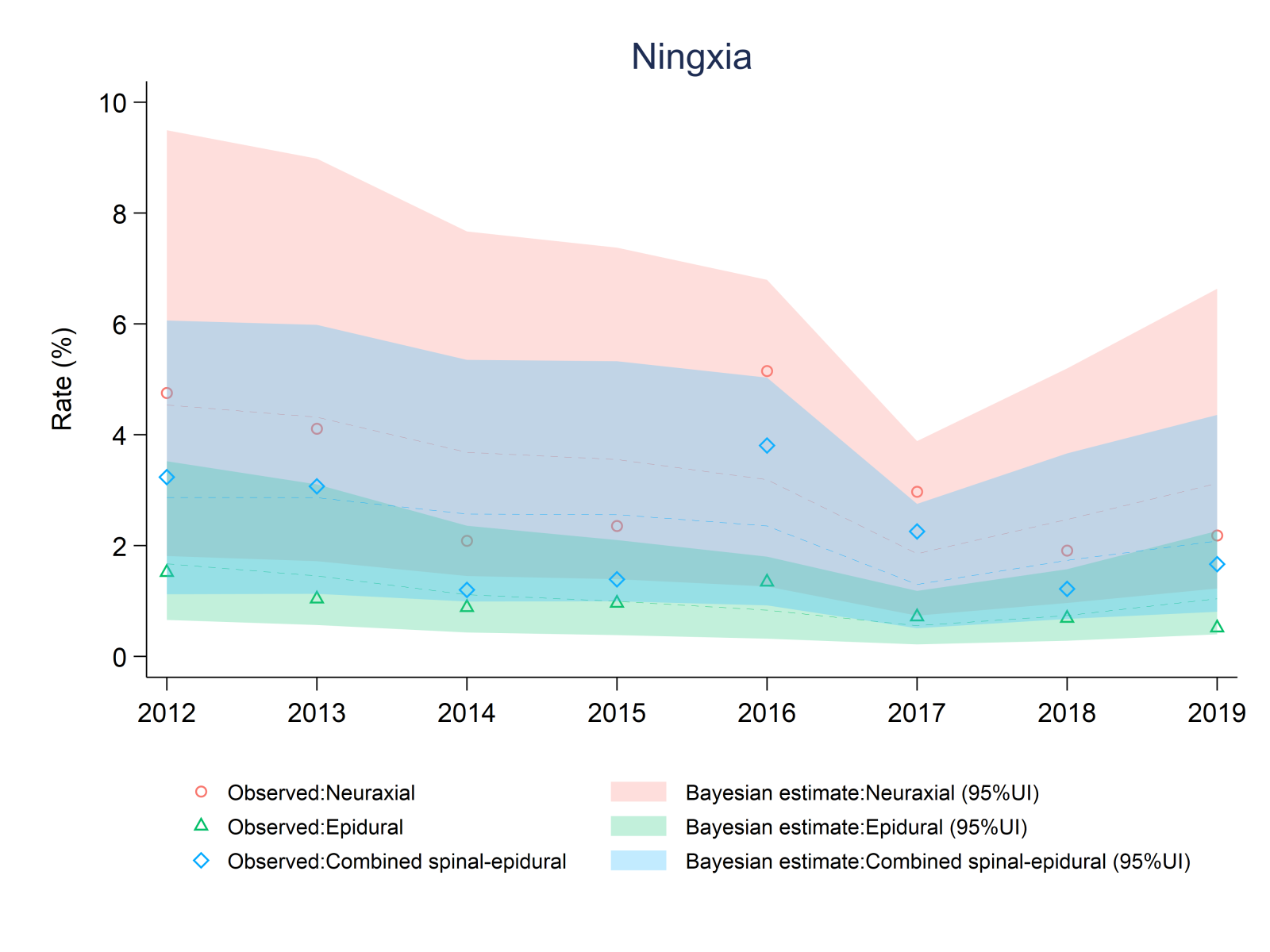


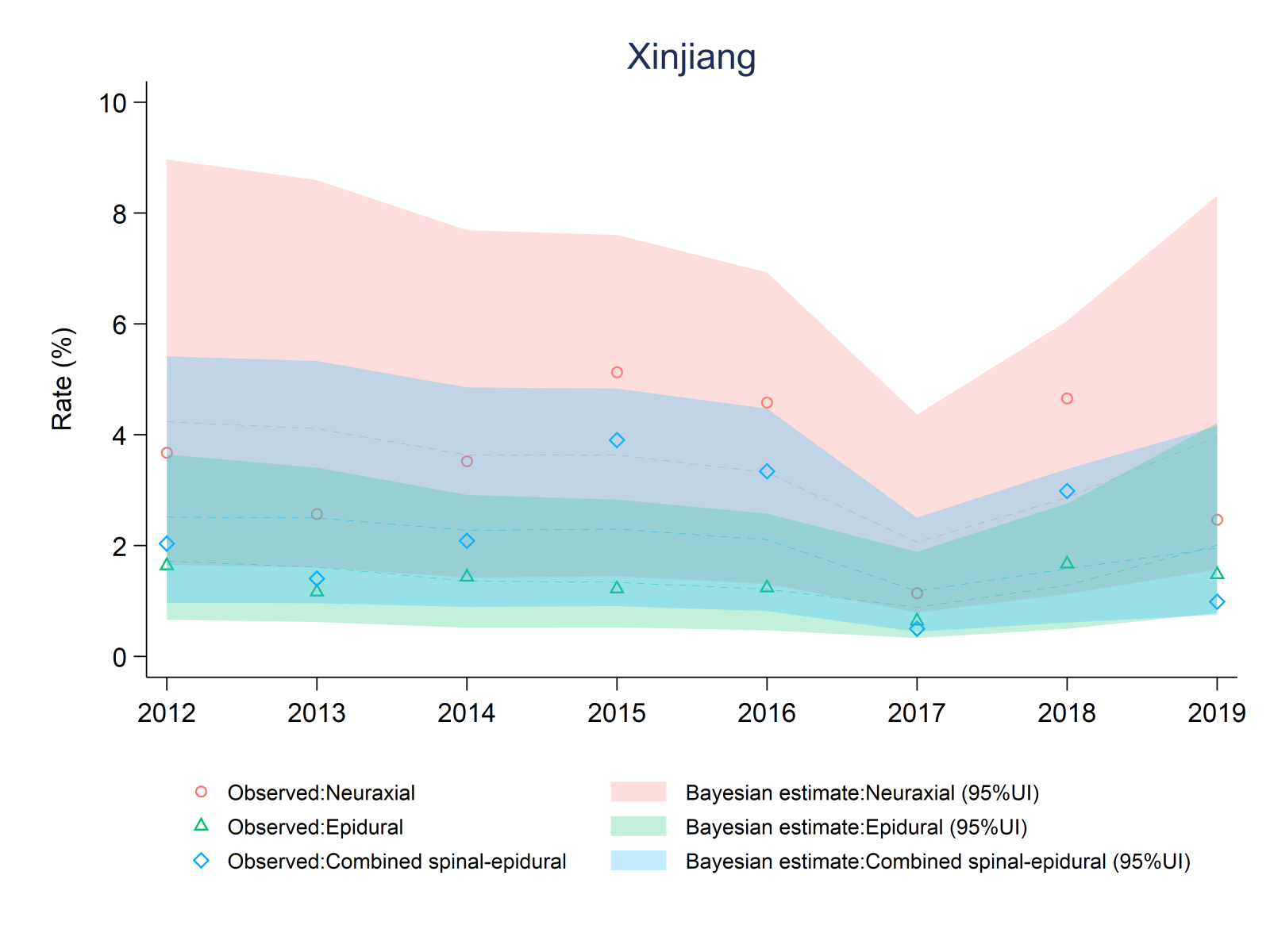


**Fig. S5**. Interrupted time-series analysis for single- and multiple-group comparisons on national monthly changes of labour neuraxial analgesia rates after the national policy

| Single-group without adjusting time-varying covariates (All hospitals) |
| --- |
| 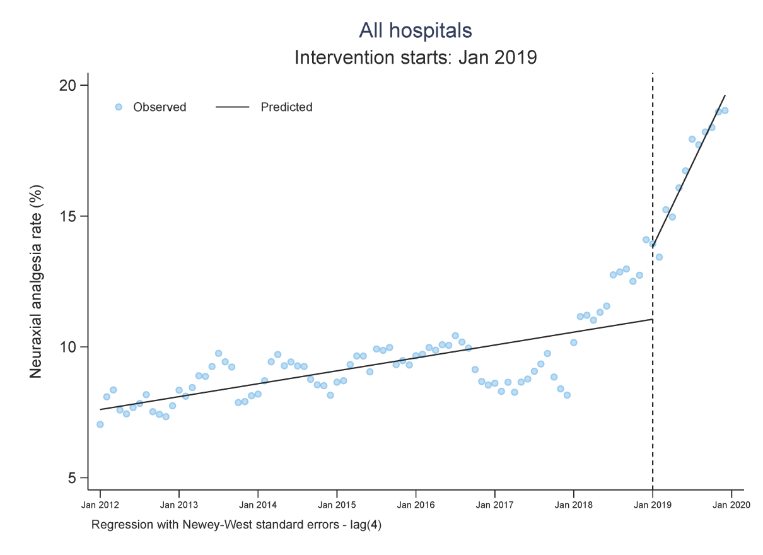 |
| Single-group adjusted for time-varying covariates (All hospitals) |
| 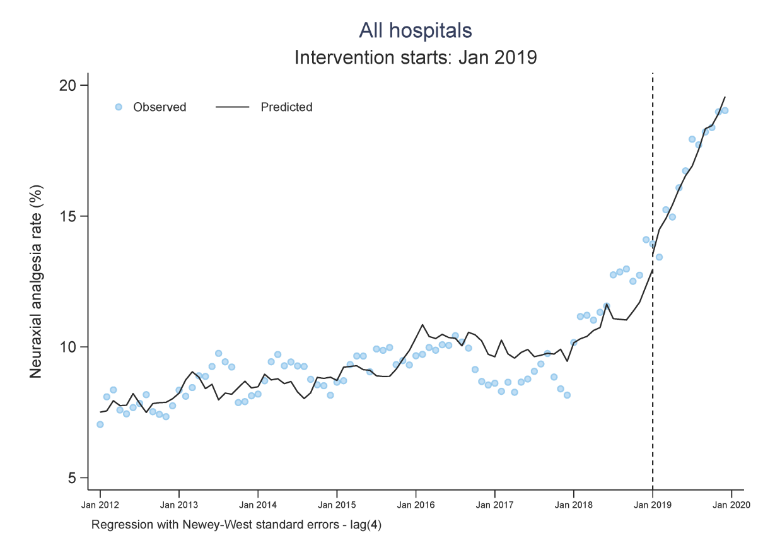 |
| Single-group without adjusting time-varying time vary covariates (Pilot hospitals) |
| 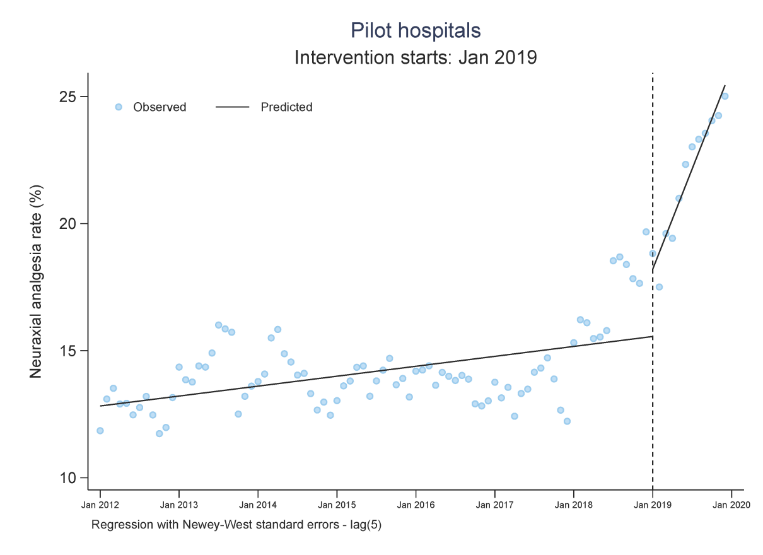 |
| Single-group adjusted for time-varying covariates (Pilot hospitals) |
| 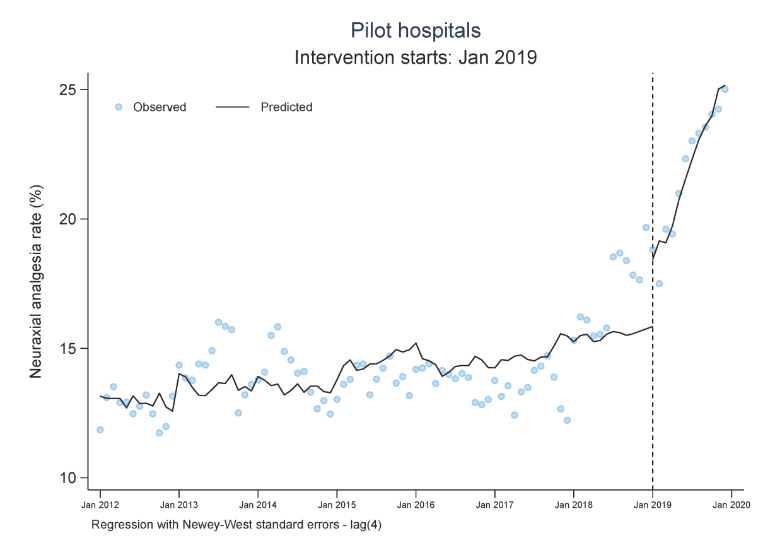 |
| Multiple-group without adjusting time-varying covariates |
| 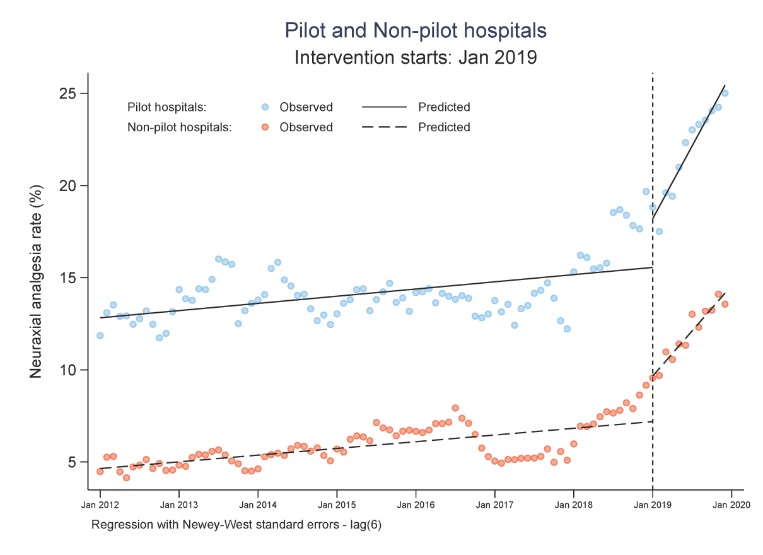 |

Note: Time-varying covariates: proportion of maternal age ≥35 years old, women with college education or above, number of antenatal visits ≥7, and women with antepartum complications or medical diseases.

**Fig. S6** Comparison of secondary maternal outcomes between women with labour neuraxial analgesia and without any analgesia

**
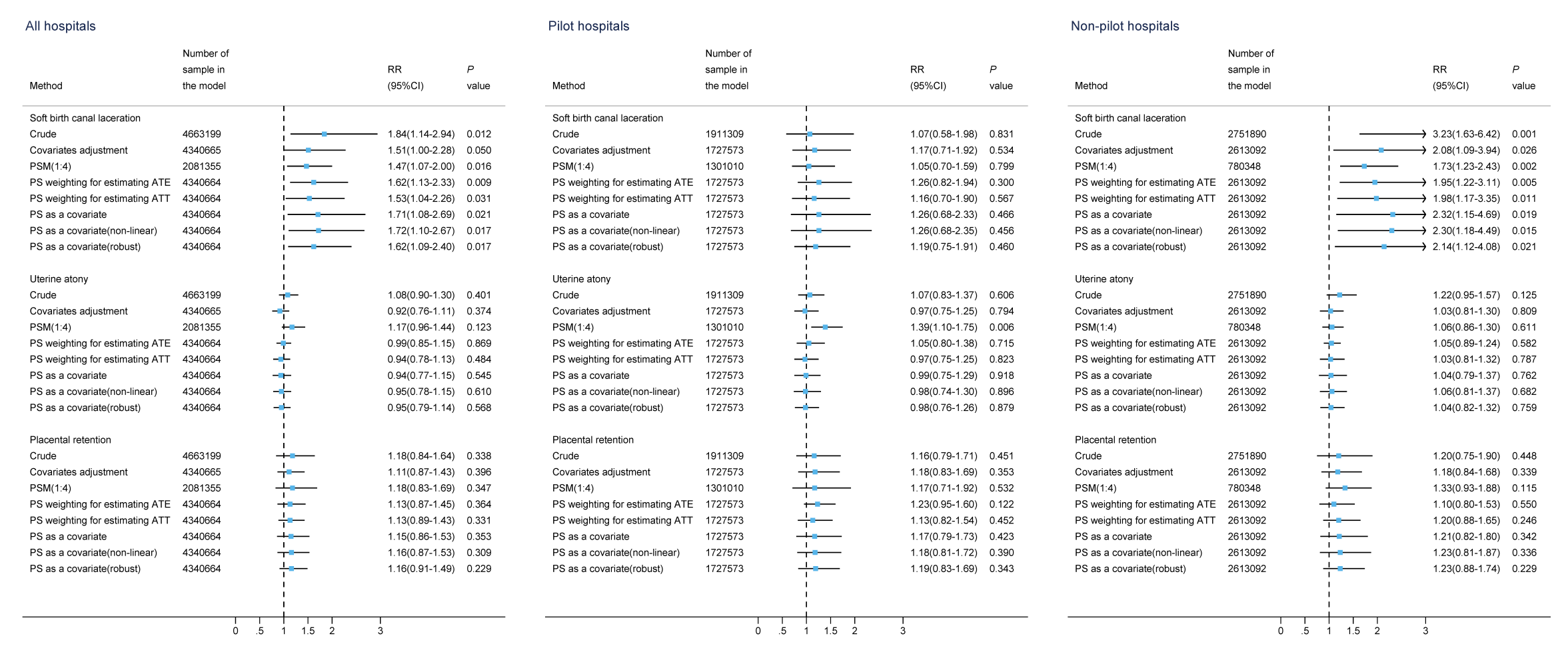
**

Notes: *PSM* propensity score matching, *PS* Propensity Score, *ATE* average treatment effect where weight is 1/*PS* for a treated case and 1/(1-*PS*) for a comparison case, *ATT* average treatment effect for the treated cases where weight is 1 for a treated case and *PS*/(1-*PS*) for a comparison case.

The left side of the reference line (short dash line in the figure) means lower risk, and the right side means higher risk.

**Fig. S7** Comparison of secondary perinatal outcomes between women with labour neuraxial analgesia and without any analgesia

**
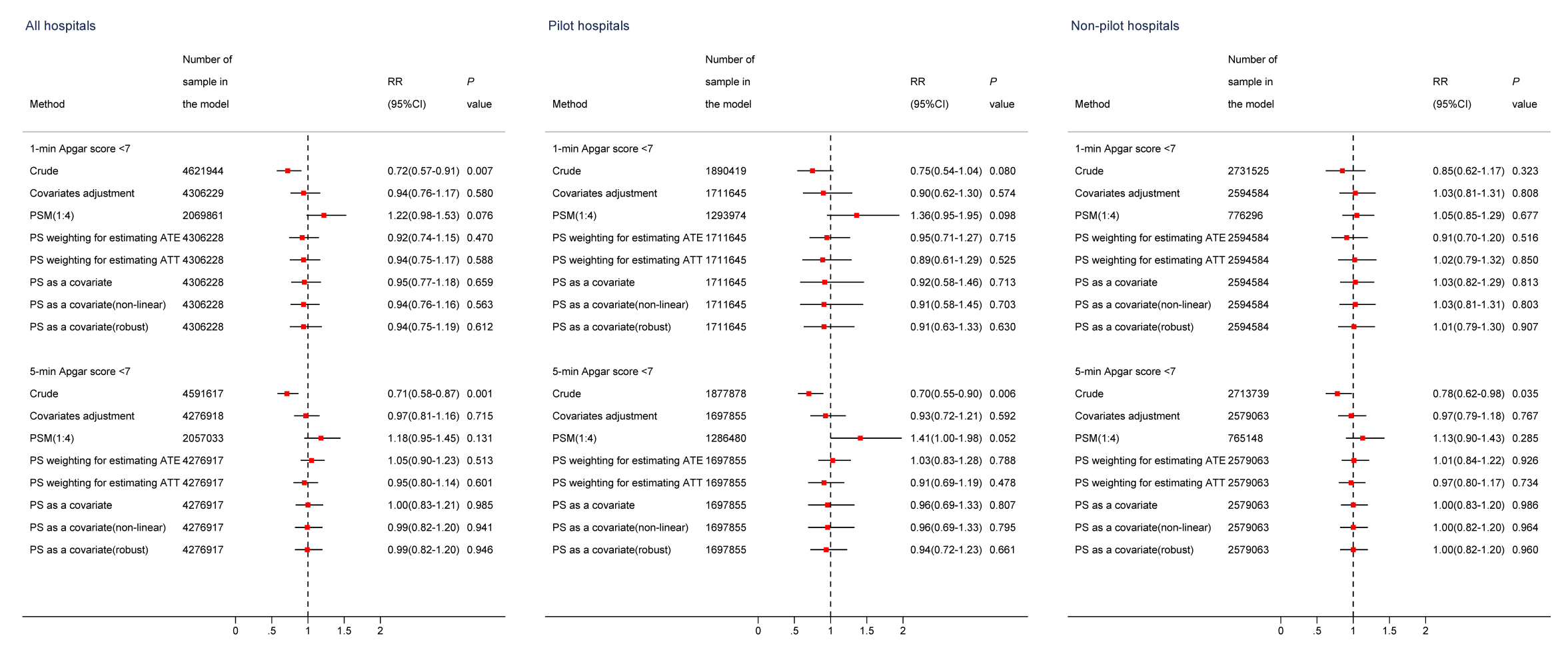
**

Notes: *PSM* propensity score matching, *PS* Propensity Score, *ATE* average treatment effect where weight is 1/*PS* for a treated case and 1/(1-*PS*) for a comparison case, *ATT* average treatment effect for the treated cases where weight is 1 for a treated case and *PS*/(1-*PS*) for a comparison case.

The left side of the reference line (short dash line in the figure) means lower risk, and the right side means higher risk.

**Fig. S8** The sensitivity analysis in all pregnant women for comparison of maternal and perinatal outcomes between women with labour neuraxial analgesia and without any analgesia

**
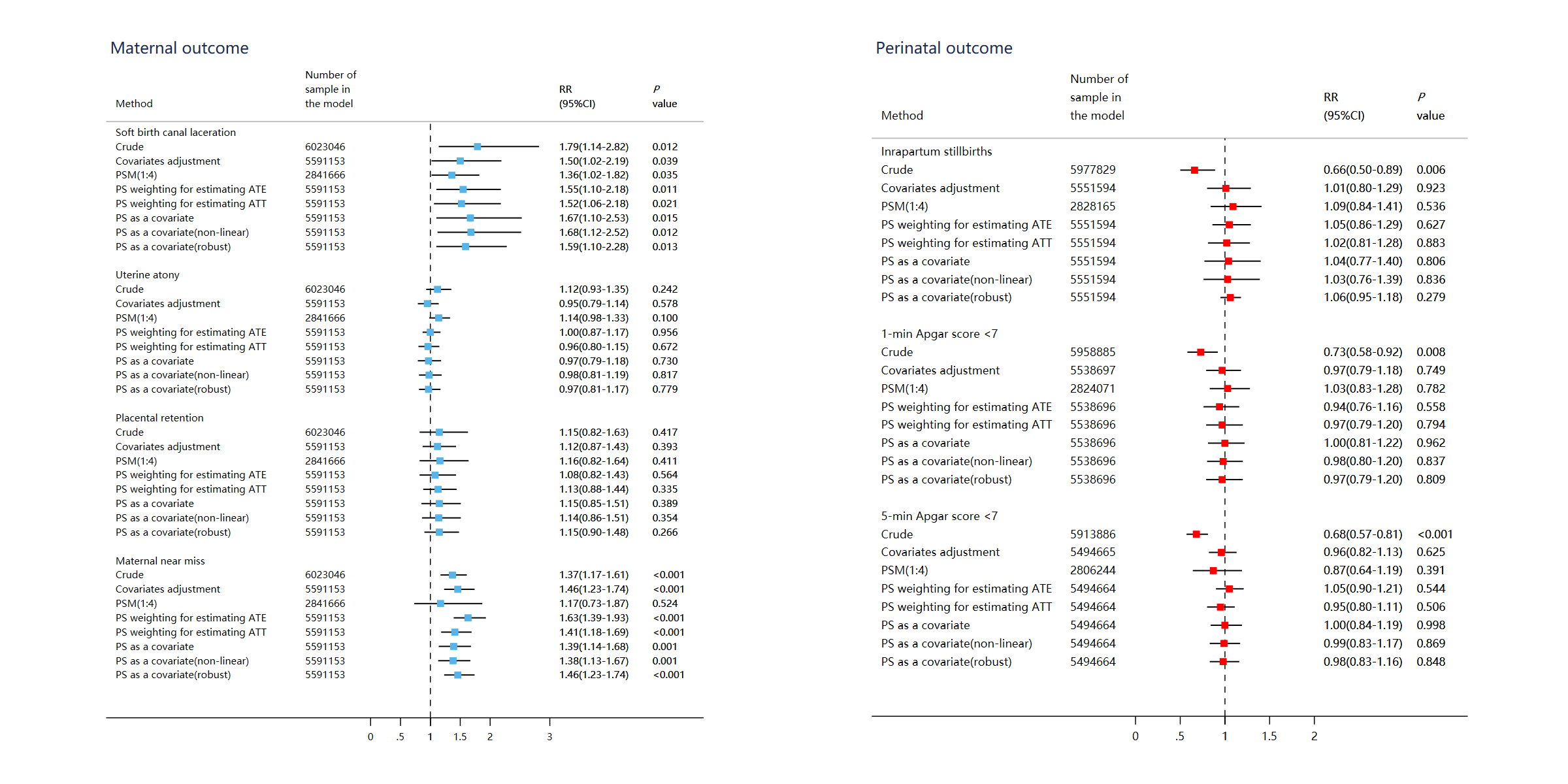
**

Notes: *PSM* propensity score matching, *PS* Propensity Score, *ATE* average treatment effect where weight is 1/*PS* for a treated case and 1/(1-*PS*) for a comparison case, *ATT* average treatment effect for the treated cases where weight is 1 for a treated case and *PS*/(1-*PS*) for a comparison case.

The left side of the reference line (short dash line in the figure) means lower risk, and the right side means higher risk.

**Fig. S9** Change of bias across covariates after propensity score matching

| All cases |
| --- |
|  |
| Live births |
|  |

**Fig. S10** The common support region of propensity scores

| All cases |
| --- |
| 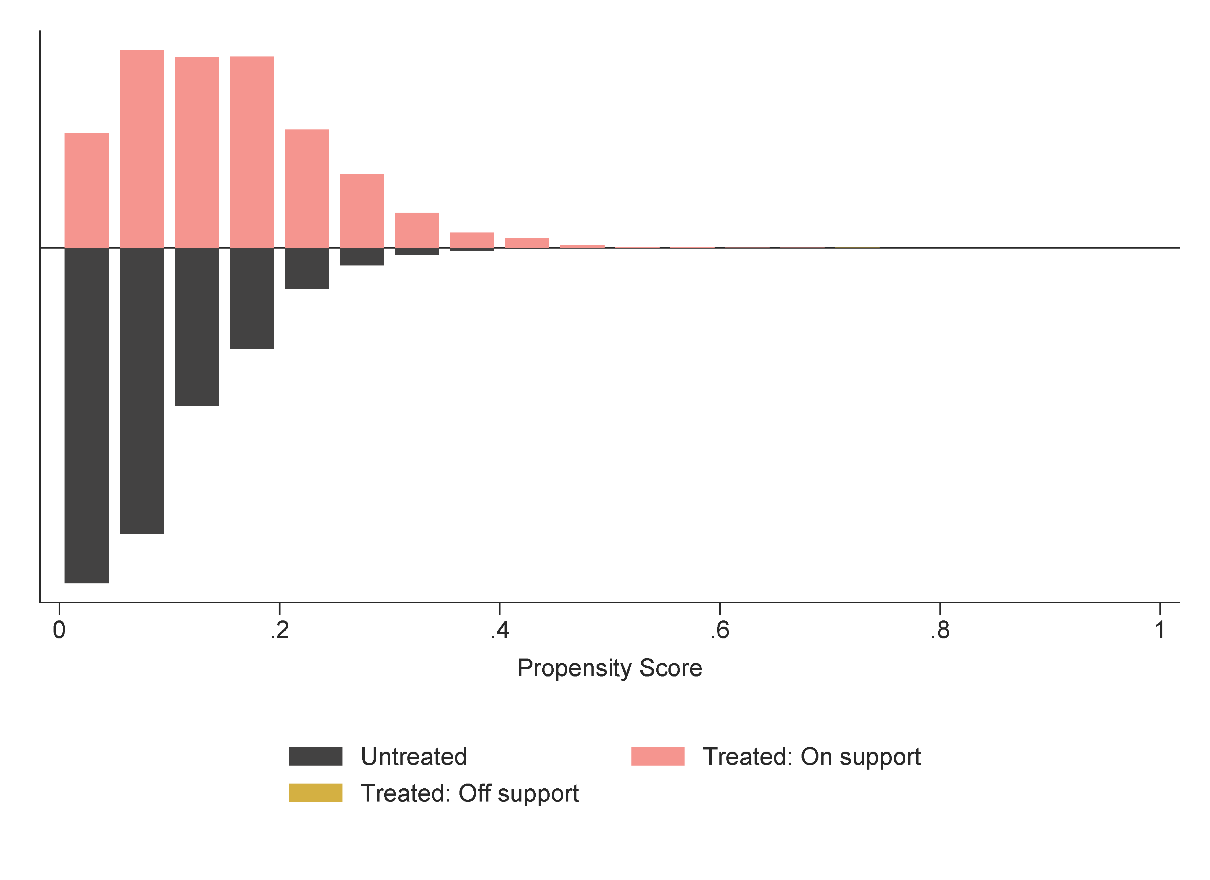 |
| Live births |
| 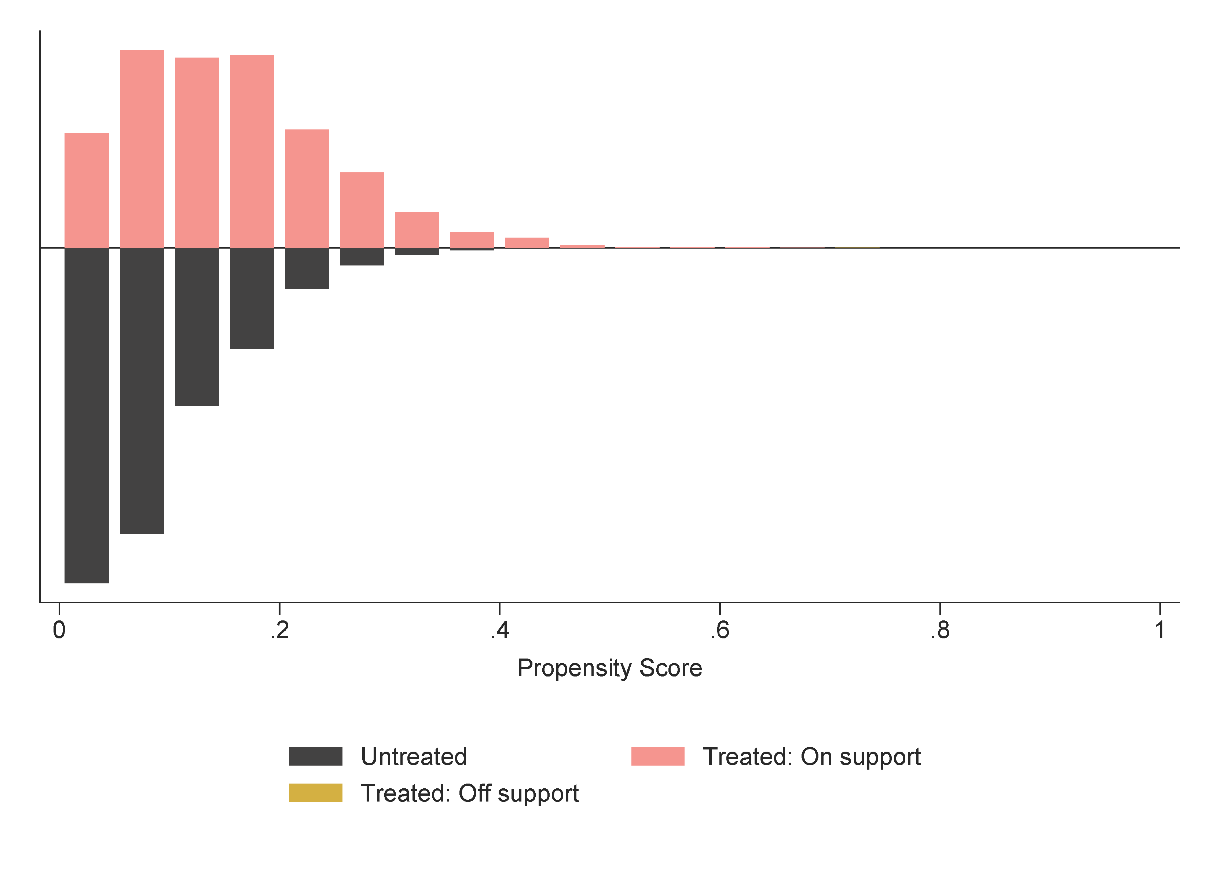 |

**Fig. S11** The E-Values for sensitivity analyses on unobserved confounding factors

| Genital tract trauma in all hospitals | Genital tract trauma in non-pilot hospitals |
| --- | --- |
| 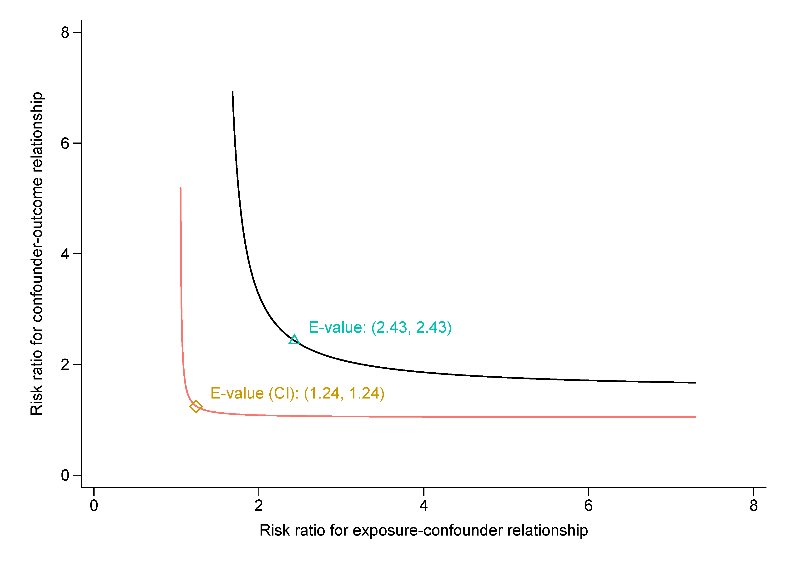 | 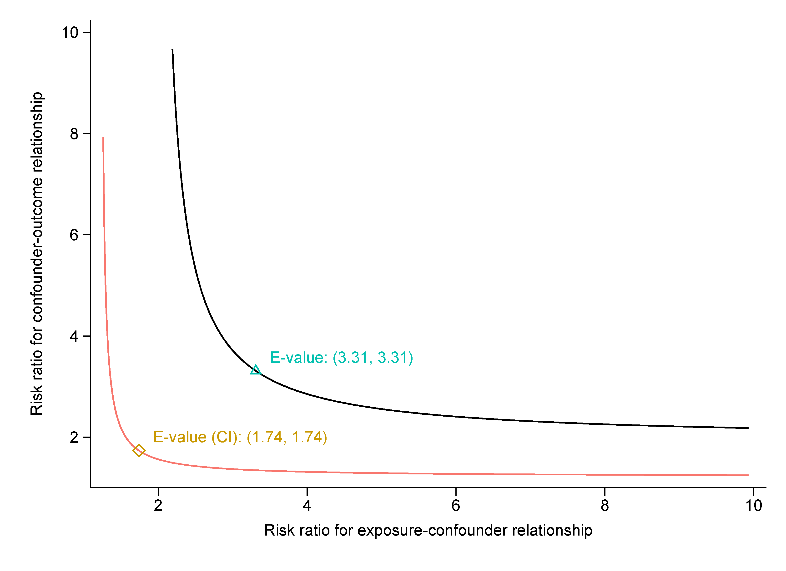 |
| Maternal near miss in all hospitals | Maternal near miss in non-pilot hospitals |
| 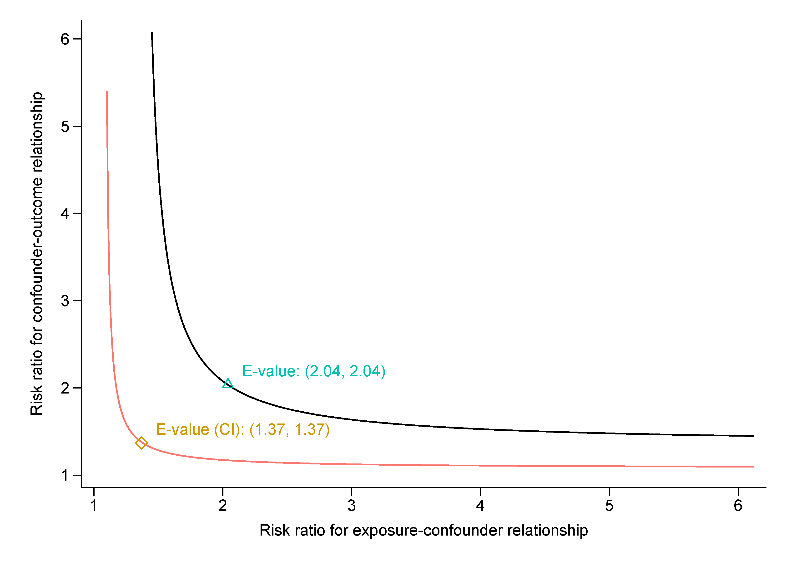 | 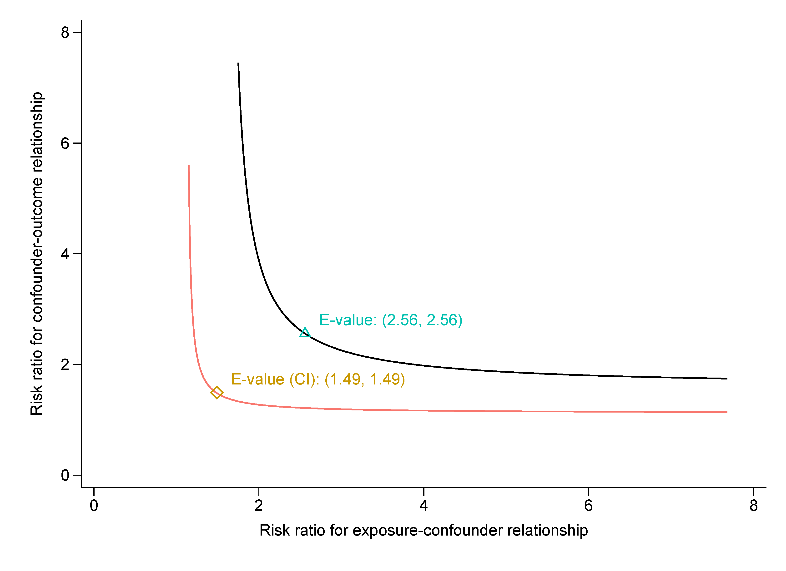 |

**Fig. S12** Conditional Marginal Effects of labour neuraxial analgesia with 95% CIs

| Genital tract trauma |
| --- |
| 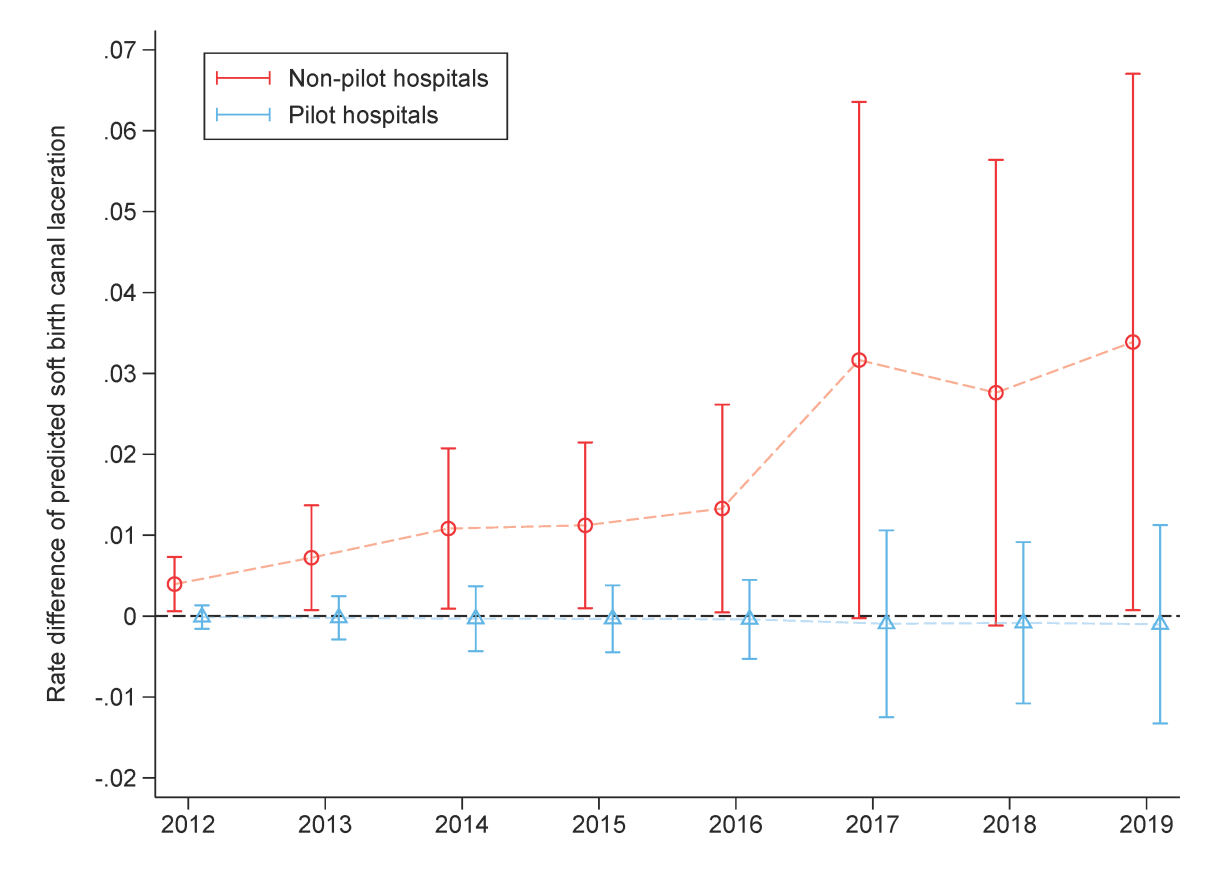 |
| Maternal near miss |
| 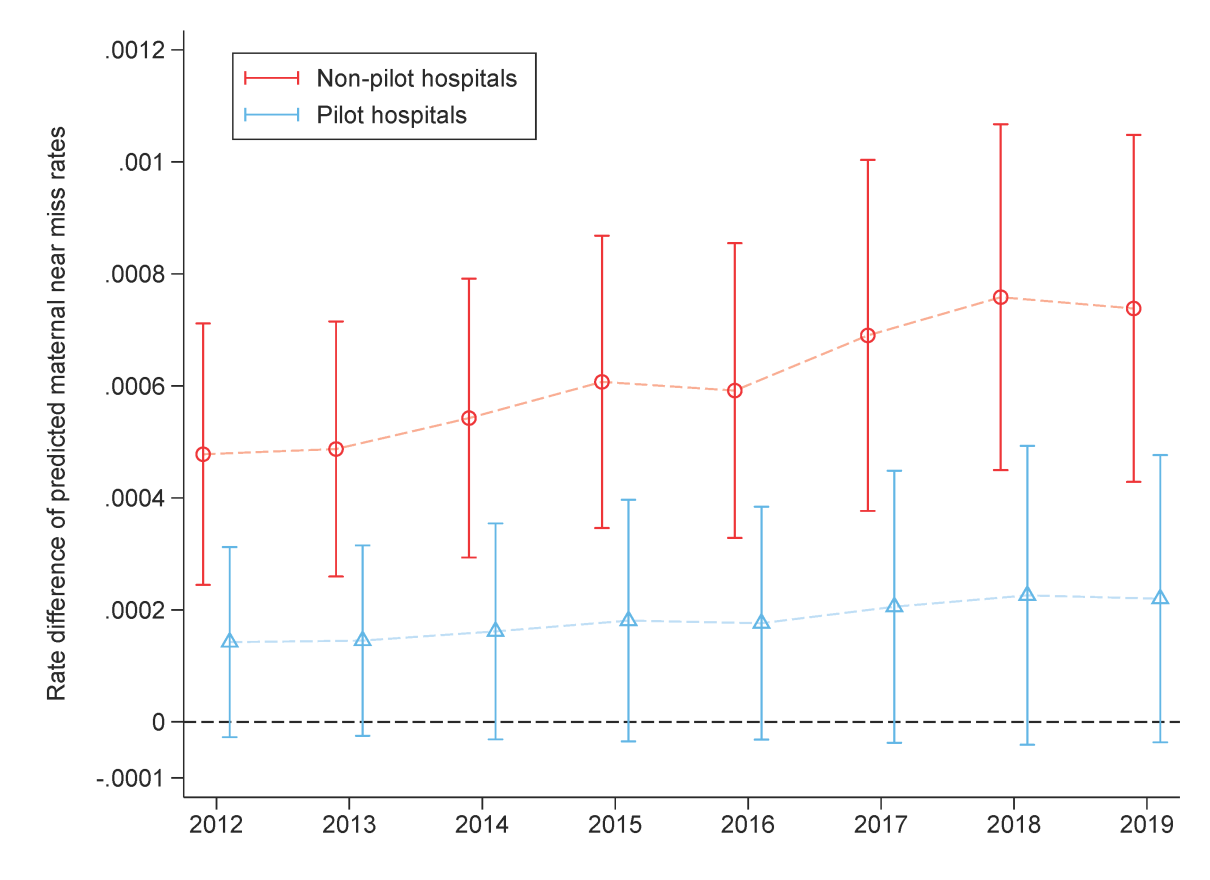 |
